# Limonoids and triterpenoids from the twigs and leaves of *Dysoxylum hainanense*

**DOI:** 10.1007/s13659-011-0030-8

**Published:** 2012-02-22

**Authors:** Wen-Xing Liu, Gui-Hua Tang, Hong-Ping He, Yu Zhang, Shun-Lin Li, Xiao-Jiang Hao

**Affiliations:** 1 State Key Laboratory of Phytochemistry and Plant Resources in West China, Kunming Institute of Botany, Chinese Academy of Sciences, Kunming, 650201 China; 2 Graduate University of Chinese Academy of Sciences, Beijing, 100049 China

**Keywords:** *Dysoxylum hainanense*, Meliaceae, limonoids, triterpenoids, cytotoxicity

## Abstract

Four new limonoids, dysohainanins A–D (**1–4**), and two new triterpenoids, dysohainanins E and F (**5** and **6**), together with seven known ones were isolated from the twigs and leaves of *Dysoxylum hainanense* Merr. The structures of the new compounds were determined by a variety of spectroscopic methods. The cytotoxic activities of these compounds were evaluated, and the known compound ent-19-nor-4,16,18-trihydroxy-8(14)-pomaren-15-one (**13**) showed *in vitro* cytotoxicity against HL-60, A-549, MCF-7, and SW480 cells, with IC_50_ values of 24.3, 28.1, 30.7, and 22.5 µM, respectively. Compounds **2** and **3** were tested their insecticidal activities using brine shrimp and both of them were inactive.

**Electronic Supplementary Material:**

Supplementary material is available for this article at 10.1007/s13659-011-0030-8 and is accessible for authorized users.

## Introduction

The genus *Dysoxylum* has a number of structurally diverse limonoids with variety of bioactivities, such as anti-feeding, cardiac, cytotoxic, and anti-microbial activities[1–7]. *D. hainanense* is a tall tree distributed mainly in Guangxi Zhuang Autonomous Region, Hainan province, and southern part of Yunnan province in China. Previous efforts on *D. hainanense* have reported a series of antifeedant limonoids, antibacterial triterpenoids, and *ent*-pimarane diterpenoids[6–14]. As a part of our ongoing phytochemical investigation on plants of Meliaceae family, four new limonoids, dysohainanins A–D (**1–4**), and two new triterpenoids, dysohainanins E and F (**5** and **6**), together with seven known ones (protoxylocarpin G (**7**)[15], 22,23-epoxy-7-tirucalla-7-ene-3$\beta$,24,25-triol (**8**)[9], 20*S*,24-epoxy-25,26,27-trisnor-24-oxo-3,4-*seco*-4(28)-dammaren-3-oicacid (**9**)[16], dysoxylumolide A (**10**)[2], dysoxylumolide C (**11**)[2], dysoxylumin C (**12**)[2], ent-19-nor-4,16,18-trihydroxy-8(14)-pomaren-15-one (**13**)[9]) were isolated from the twigs and leaves of *D. hainanense*. The structures of the new compounds were established on the basis of extensive 1D and 2D NMR experiments including $^1$H-$^1$H COSY, HSQC, HMBC, and ROESY. The cytotoxic and insecticidal activities of these isolated compounds were also evaluated.

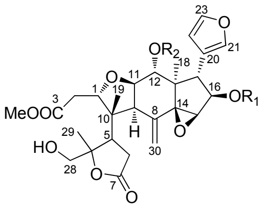

1

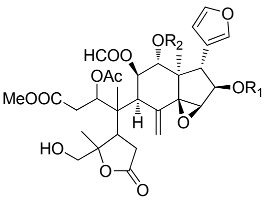

2

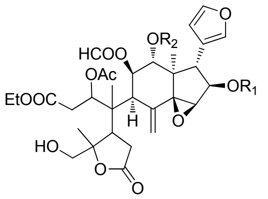

3

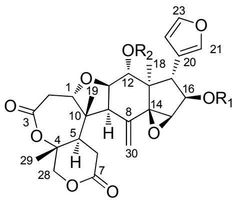

4

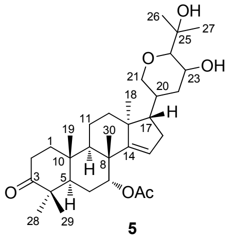

5

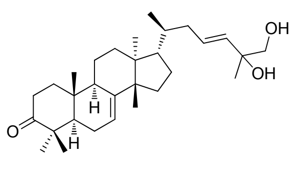

6

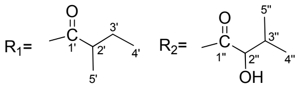

*To whom correspondence should be addressed. E-mail: haoxj@mail.kib.ac.cn; lisl@mail.kib.ac.cn

## Results and Discussion

Dysohainanin A (**1**) possessed the molecular formula of $C_{37}H_{50}O_{13}$ deduced by the positive ion HRESIMS [*m/z* 725.3133 $[M + Na]^+$ (calcd. for 725.3149)] with 13 degrees of unsaturation. Absorption bands in its IR spectrum suggested obviously the presence of hydroxyl and carbonyl groups. The 1D (see Tables 1 and 2) and 2D NMR data of **1** exhibited a methoxy group [$\delta_C$ 52.4; $\delta_H$ 3.70 (3H, s)], a 2-methyl-butyrate group [$\delta_C$ 177.2 (qC, C-1'), 40.9 (CH, C-2'), 27.0 ($CH_2$, C-3'), 11.2 ($CH_3$, C-4'), 16.5 ($CH_3$, C-5'); $\delta_H$ 2.36 (1H, m, H-2'), 1.50 (1H, m, H-3'a), 1.38 (1H, m, H-3'b), 0.65 (3H, t, $J$ = 7.4 Hz, H-4'), 1.07 (3H, d, $J$ = 6.9 Hz, H-5')], and a 2-hydroxy-3-methyl-butyrate group [$\delta_C$ 175.1 (qC, C-1″), 74.7 (CH, C-2″), 32.3 (CH, C-3″), 15.7 ($CH_3$, C-4″), 19.2 ($CH_3$, C-5″); $\delta_H$ 3.65 (1H, br. s, H-2″), 1.95 (1H, m, H-3″), 0.79 (3H, d, $J$ = 6.9 Hz, H-4″), 0.96 (3H, d, $J$ = 6.9 Hz, H-5″)]. Except of the above mentioned substituents, compound **1** contained 26 carbons (see table 1) consisting of three tertiary methyls, three methylenes (including an oxygenated one), eight methines (including five oxygenated ones), four carbons indicated a typical $\beta$-substituted furan ring, two olefinic carbons, and other six quaternary carbons (including two carboyl one). These data suggested that **1** and dysoxylumic acid C [6] had the identical core structure, which was elucidated extensively by the 2D NMR data (see Fig. 1) of **1**. The linkages of the methoxy group to C-3, the 2-methyl-butyrate group to C-16, and the 2-hydroxy-3-methyl-butyrate group to C-12 were determined by the HMBC correlations of protons at $\delta_H$ 3.70 (s, 3-OC$H_3$) to C-3 at $\delta_C$ 170.9, proton at $\delta_H$ 5.29 (dd, $J$ = 9.4, and 0.5 Hz, H-16) to C-1' at $\delta_C$ 177.2, and proton at $\delta_H$ 5.65 (d, $J$ = 7.5 Hz, H-12) to C-1″at $\delta_C$ 175.1, respectively.

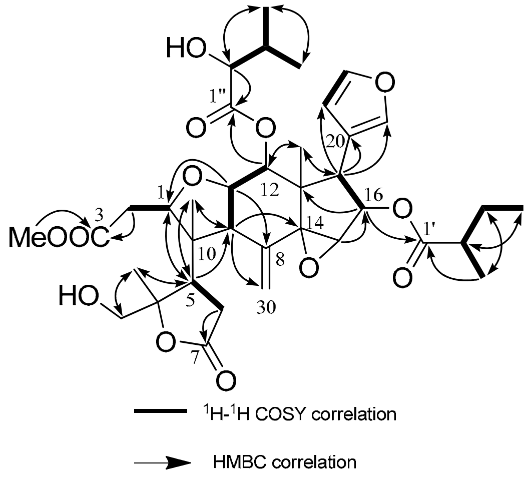

**Fig. 1.** Selected HMBC and $^1$H-$^1$H COSY correlations of **1**

The relative stereochemistry of **1** was determined by its ROESY experiment. Correlations of H-11/Me-18, Me-18/H-16, and H-15/H-16, Me-18/H-22, and Me-18/H-23 indicated that H-11, H-16, H-15, Me-18, and the $\beta$-substituted furan ring were co-facial, and were arbitrarily assigned these groups a $\alpha$-orientation, while 16-OR$_1$, and H-17 was $\beta$-oriented. The correlations of H-1/H-12, H-17/H-12, and Me-19/H-12 were deduced H-1, H-12, and Me-19 to be $\beta$-orientation. Although there were no obvious cross-peaks of H-9 with other key protons in the ROESY spectrum, the $\alpha$-orientation of H-9 was determined by comparison of the NMR data with those of dysoxylumic acid C. So the structure of **1** was determined as shown and named as dysohainanin A.

**Table 1.** $^{13}$**C NMR data (1 in 150 MHz, 2–4 in 125 MHz) of 1–4 (in CDCl$_3$)**

| poition | 1 | 2 | 3 | 4 |
|---|---|---|---|---|
| 1 | 84.0, CH | 73.1, CH | 73.2, CH | 79.7, CH |
| 2 | 35.2, $CH_2$ | 35.3, $CH_2$ | 35.7, $CH_2$ | 37.5, $CH_2$ |
| 3 | 170.9, C | 171.0, C | 170.5, C | 167.3, C |
| 4 | 89.5, C | 91.3, C | 91.4, C | 79.0, C |
| 5 | 42.6, CH | 42.7, CH | 42.7, CH | 44.5, CH |
| 6 | 33.0, $CH_2$ | 34.2, $CH_2$ | 34.2, $CH_2$ | 31.0, $CH_2$ |
| 7 | 175.3, C | 175.1, C | 175.3, C | 170.9, C |
| 8 | 136.6, C | 135.9, C | 135.8, C | 135.6, C |
| 9 | 50.0, CH | 53.3, CH | 53.2, CH | 54.9, CH |
| 10 | 49.0, C | 47.8, C | 47.8, C | 51.0, C |
| 11 | 79.4, CH | 69.1, CH | 68.9, CH | 78.9, CH |
| 12 | 79.3, CH | 74.5, CH | 74.6, CH | 74.0, CH |
| 13 | 44.0, C | 45.0, C | 45.0, C | 43.6, C |
| 14 | 70.5, C | 68.9, C | 68.9, C | 69.6, C |
| 15 | 59.4, CH | 58.8, CH | 58.8, CH | 58.9, CH |
| 16 | 76.9, CH | 76.2, CH | 76.2, CH | 76.4, CH |
| 17 | 42.6, CH | 42.2, CH | 42.2, CH | 42.1, CH |
| 18 | 15.7, $CH_3$ | 14.9, $CH_3$ | 14.9, $CH_3$ | 14.7, $CH_3$ |
| 19 | 19.2, $CH_3$ | 16.2, $CH_3$ | 16.2, $CH_3$ | 17.2, $CH_3$ |
| 20 | 120.0, C | 119.1, C | 119.1, C | 119.6, C |
| 21 | 141.2, CH | 141.3, CH | 141.3, CH | 140.9, CH |
| 22 | 111.0, CH | 110.8, CH | 110.8, CH | 110.9, CH |
| 23 | 143.3, CH | 143.2, CH | 143.2, CH | 143.1, CH |
| 28 | 68.4, $CH_2$ | 66.0, $CH_2$ | 66.0, $CH_2$ | 71.9, $CH_2$ |
| 29 | 20.3, $CH_3$ | 19.0, $CH_3$ | 19.0, $CH_3$ | 26.5, $CH_3$ |
| 30 | 121.7, $CH_2$ | 123.6, $CH_2$ | 123.7, $CH_2$ | 121.7, $CH_2$ |
| 1′ | 177.2, C | 176.7, C | 176.7, C | 176.9, C |
| 2′ | 40.9, CH | 40.6, CH | 40.6, CH | 40.7, CH |
| 3′ | 27.0, $CH_2$ | 26.8, $CH_2$ | 26.8, $CH_2$ | 26.7, $CH_2$ |
| 4′ | 11.2, $CH_3$ | 10.9, $CH_3$ | 10.9, $CH_3$ | 10.9, $CH_3$ |
| 5′ | 16.5, $CH_3$ | 16.2, $CH_3$ | 16.2, $CH_3$ | 16.3, $CH_3$ |
| 1″ | 175.1, C | 174.6, C | 174.6, C | 175.0, C |
| 2″ | 74.7, CH | 74.6, CH | 74.5, CH | 74.7, CH |
| 3″ | 32.3, CH | 31.2, CH | 31.2, CH | 32.1, CH |
| 4″ | 15.7, $CH_3$ | 19.0, $CH_3$ | 19.0, $CH_3$ | 15.6, $CH_3$ |
| 5″ | 19.2, $CH_3$ | 15.1, $CH_3$ | 15.1, $CH_3$ | 18.5, $CH_3$ |
| 11-OOCH | | 161.0, CH | 160.9, CH | |
| 3-OMe | 52.4, $CH_3$ | 52.2, $CH_3$ | | |
| 1-OAc | | 170.1, C | 170.2, C | |
| | | 20.9, $CH_3$ | 20.9, $CH_3$ | |
| 3-OEt | | | 61.1, $CH_2$ | |
| | | | 14.0, $CH_3$ | |

**Table 2.** $^{1}$**H NMR data (1 in 600 MHz, 2–4 in 500 MHz) of 1–4 in CDCl$_3$**

| position | 1 | 2 | 3 | 4 |
|---|---|---|---|---|
| 1 | 4.18, dd (10.6, 2.1) | 5.60, d (11.4) | 5.61, d (11.2) | 4.05, dd (10.2. 5.6) |
| 2a | 2.58, dd (14.3, 2.1) | 2.95, d (15.5) | 2.96, d (15.5) | 2.95, dd (13.1, 5.6) |
| 2b | 2.43, dd (14.3, 10.6) | 2.55, dd (15.5, 11.4) | 2.54, dd (15.5, 11.2) | 2.75, dd (13.1, 10.2) |
| 5 | 2.82, d (10.0) | 2.80, m | 2.83, d (5.9) | 2.37, t (9.2) |
| 6a | 2.75, d (8.7) | 2.77, m | 2.79, d (16.9) | 2.70, d (9.2) |
| 6b | 2.60, m | 2.44, dd (16.9, 5.6) | 2.45, dd (16.9, 5.9) | 2.63, d (9.2) |
| 9 | 3.51, d (9.6) | 3.31, d (7.4) | 3.32, d (7.3) | 3.16, d (8.7) |
| 11 | 4.20, dd (9.6, 7.5) | 5.45, m | 5.46, m | 4.15, m |
| 12 | 5.65, d (7.5) | 5.87, d (11.0) | 5.88, d (11.0) | 5.56, d, (9.2) |
| 15 | 4.08, br. s | 4.11, s | 4.12, br. s | 4.04, br. s |
| 16 | 5.29, dd (9.4, 0.5) | 5.34, d (9.2) | 5.35, d (9.2) | 5.20, d (9.2) |
| 17 | 3.19, d (9.4) | 3.14, d (9.2) | 3.16, d (9.2) | 3.03, d (9.2) |
| 18 | 0.87, s | 1.06, s | 1.08, s | 0.84, s |
| 19 | 1.15, s | 1.50, br. s | 1.52, s | 1.19, s |
| 21 | 7.12, s | 7.14, s | 7.16, s | 7.06, s |
| 22 | 6.14, s | 6.18, s | 6.20, s | 6.07, s |
| 23 | 7.32, s | 7.33, br. s | 7.35, s | 7.27, s |
| 28a | 3.53, d (12.6) | 3.82, s | 3.84, s | 4.16, m |
| 28b | 3.73, d (12.6) | | | 3.92, d (11.2) |
| 29 | 1.47, s | 1.61, s | 1.63, s | 1.57, s |
| 30a | 5.42, s | 5.55, s | 5.56, s | 5.48, s |
| 30b | 5.38, s | 5.37, s | 5.39, s | 5.33, s |
| 2′ | 2.36, m | 2.37, m | 2.37, m | 2.29, m |
| 3′a | 1.50, m | 1.48, m | 1.49, m | 1.42, m |
| 3′b | 1.38, m | 1.37, m | 1.38, m | 1.30, m |
| 4′ | 0.65, t (7.4) | 0.63, t (7.4) | 0.62, t (7.4) | 0.59, t (7.4) |
| 5′ | 1.07, d (6.9) | 1.10, d (6.9 ) | 1.11, d (6.9) | 1.00, d (6.9) |
| 2″ | 3.65, br. s | 3.45, br. s | 3.47, s | 3.61, s |
| 3″ | 1.95, m | 1.76, m | 1.78, m | 1.87, m |
| 4″ | 0.79, d (6.9) | 0.94, d (6.9) | 0.96, d (6.9) | 0.75, d (6.9) |
| 5″ | 0.96, d (6.9 ) | 0.73, d (6.9) | 0.74, d (6.9) | 0.91, d (6.9) |
| 11-OOCH | | 8.04, s | 8.06, s | |
| 3-OMe | 3.70, s | 3.67, s | | |
| 1-OAc | | 2.03, s | 2.05, s | |
| 3-OEt | | | 4.13, m | |
| | | | 1.25, t (7.2) | |

Dysohainanin B (**2**) was isolated as colorless crystal. The molecular formula of **2** was established as $C_{40}H_{54}O_{16}$ by the positive HRESIMS ion at *m/z* 813.3309 $[M + Na]^+$ (calcd. for 813.3310) with 14 degrees of unsaturation. Comparison the $^{1}$H and $^{13}$C NMR data of **2** with those of **1** (see Tables 1 and 2) showed that **2** was very similar to dysohainanin A (**1**). The differences between them were that the five-membered oxygen ring formed through C-1 and C-11 was disappeared in **2** instead of an acetyl group and a formyl group located at C-1 ($\delta_C$ 73.1) and C-11 ($\delta_C$ 69.1), respectively. The two additional substituents supposed to link at C-1 and C-11 were further confirmed by the detail analysis of its 2D NMR spectra.

The relative stereochemistry at the chiral centers of carbons C-9–C-17 in **2** was determined as the same with **1** by the cross peaks observed in its ROESY spectrum. However, the stereochemistry at the other chiral centers in **2** could not be determined by the ROESY experiment. Thus, the structure of **2** was elucidated to be dysohainanin B as shown.

Dysohainanin (**3**), a colorless crystal, had the molecular formula of $C_{41}H_{56}O_{16}$ deduced by the positive ion HRESIMS. Detail analysis of its 1D NMR (see Tables 1 and 2) and MS data with those of **2** showed that an ethoxy group [$\delta_C$ 61.1 ($CH_2$), 14.0 ($CH_3$); $\delta_H$ 4.13 (2H, m), 1.25 (3H, t, $J$ = 7.2 Hz)] in **3** replaced the methoxy group located at C-1 in **2**. The HMBC correlation of protons at $\delta_H$ 4.13 (2H, m, -OC$H_2$CH$_3$) to carbon at $\delta_C$ 170.5 (C-3) strongly suggested that the presence of the ethoxy group was linked to C-3. The relative stereochemistry of **3** was deduced as the same with **2** by analysis of its ROSEY spectrum. Therefore, the structure of **3** was established.

**Table 3. $^{1}$H NMR (500 MHz) and $^{13}$C NMR (125 MHz) data of 5 and 6 in CDCl$_3$**

| position | 5 | | 6 | |
|---|---|---|---|---|
| | $\delta_H$ | $\delta_C$ | $\delta_H$ | $\delta_C$ |
| 1a | 1.90, m | 38.8, CH$_2$ | 1.99, m | 38.5, CH$_2$ |
| 1b | 1.49, m | | 1.45, m | |
| 2a | 2.57, m | 33.9, CH$_2$ | 2.75, m | 34.9, CH$_2$ |
| 2b | 2.41, m | | 2.25, m | |
| 3 | | 216.9, C | | 217.0, C |
| 4 | | 46.9, C | | 47.8, C |
| 5 | 1.83, m | 48.2, CH | 1.72, m | 52.3, CH |
| 6a | 1.87, m | 24.3, CH$_2$ | 1.27, m | 24.3, CH$_2$ |
| 6b | 1.70, m | | 2.07, m | |
| 7 | 5.20, dd, (1.9, 3.5) | 75.3, CH | 5.30, d (3.0) | 117.9, CH |
| 8 | | 42.0, C | | 145.8, C |
| 9 | 2.00, m | 43.0, CH | 2.28, m | 48.3, CH |
| 10 | | 36.9, C | | 34.9, C |
| 11a | 1.67, m | 16.7, CH$_2$ | 1.55, m | 18.2, CH$_2$ |
| 11b | 1.55, m | | | |
| 12a | 2.26, m | 34.8, CH$_2$ | 1.82, m | 33.6, CH$_2$ |
| 12b | 1.55, m | | 1.65, m | |
| 13 | | 46.4, C | | 43.5, C |
| 14 | | 159.2, C | | 51.2, C |
| 15a | 5.29, d (2.7) | 119.2, CH | 1.48, m | 33.9, CH$_2$ |
| 15b | | | 1.55, m | |
| 16a | 2.26, m | 34.8, CH$_2$ | 1.29, m | 28.2, CH$_2$ |
| 16b | 1.93, m | | 1.93, m | |
| 17 | 1.97, m | 52.2, CH | 1.52, m | 53.0, CH |
| 18 | 0.94, s | 19.9, CH$_3$ | 0.83, s | 22.2, CH$_3$ |
| 19 | 1.01, s | 15.2, CH$_3$ | 1.00, s | 12.7, CH$_3$ |
| 20 | 1.51, m | 35.8, CH | 1.48, m | 36.0, CH |
| 21a | 3.95, d (11.5) | 70.0, CH$_2$ | 0.82, d (5.4) | 18.8, CH$_3$ |
| 21b | 3.42, dd (11.5. 2.0) | | | |
| 22a | 2.00, m | 36.3, CH$_2$ | 2.36, m | 38.1, CH$_2$ |
| 22b | 1.51, m | | 1.69, m | |
| 23 | 3.86, m | 64.4, CH | 5.70, dd (15.6, 7.9) | 129.5, CH |
| 24 | 2.87, d (9.0) | 86.5, CH | 5.46, d (15.6) | 134.6,CH |
| 25 | | 74.1, C | | 73.1, C |
| 26a | 1.25, s | 24.0, CH$_3$ | 3.42, d (10.9) | 70.0, CH$_2$ |
| 26b | | | 3.49, d (10.9) | |
| 27 | 1.29, s | 28.5, CH$_3$ | 1.26, s | 24.4, CH$_3$ |
| 28 | 0.99, s | 25.8, CH$_3$ | 1.11, s | 21.6, CH$_3$ |
| 29 | 1.01, s | 20.9, CH$_3$ | 1.04, s | 24.5, CH$_3$ |
| 30 | 1.15, s | 26.9, CH$_3$ | 1.00, s | 27.4, CH$_3$ |
| 7-OAc | | 170.2, C | | |
| | 1.94, s | 21.2, CH$_3$ | | |

Dysohainanin D (**4**) had the molecular formula of $C_{36}H_{46}O_{12}$ on the basis of positive ion HRESIMS [*m/z* 693.2871 [M + Na]$^+$ (calcd. for 693.2887)]. Its IR spectrum showed the presence of hydroxyl (3448 cm$^{-1}$), and carbonyl (1743 cm$^{-1}$) groups. The $^{1}$H NMR and $^{13}$C NMR spectra of **4** showed very similarity to those of dysoxylumolide A[6], apart from a 2-hydroxy-3-methyl-butyrate in dysoxylumolide A was replaced by a 2-methyl-butyrate group [$^{1}$H-$^{1}$H COSY correlations of proton at $\delta_H$ 2.29 (m, H-2′) with proton at $\delta_H$ 1.30 (m, H-3′b) and protons at $\delta_H$ 1.00 (d, $J$ = 6.9, H-5′), protons at $\delta_H$ 0.59 (t, $J$ = 7.4, H-4′) with proton at $\delta_H$ 1.42 (m, H-3′a), together with the HMBC correlation of H-2′ to $\delta_C$ 176.9 (C-1′)] in **4**. The HMBC correlation of $\delta_H$ 5.20 (d, $J$ = 9.2, H-16) to C-1′ suggested the 2-methyl-butyrate group was located at C-16. In the ROESY spectrum, the correlations of H-5/H-9, H-9/H-11, H-11/Me-18, Me-18/H-16 and H-16/H-15 revealed that H-5, H-9, H-11, H-15, H-16 and Me-18 possession the same configuration and were attributed as $\alpha$ configuration; on the contrary, H-1, H-12, H-17, Me-19 and Me-29 determined as $\beta$ configuration on the basis of literature and the correlations of H-1/H-12, H-12/ H-17, and Me-19/H-1. Hence, the structure of **4** was well established and named as dysohainanin D.

The molecular formula of dysohainanin E (**5**) was deduced to be $C_{32}H_{50}O_6$ by its HREIMS [*m/z* 530.3594 [M]$^+$ (calcd. for 530.3607)]. Analysis the $^{1}$H and $^{13}$C NMR data of **5** with those of turrapubesol B[13] showed that **5** had the same skeleton type with the known compound with the exception of different substituents and number of double bonds. The $^{1}$H and $^{13}$C NMR data of **5** showed an acetyl group [$\delta_H$ 1.94 (3H, s); $\delta_C$ 170.2, 21.2) instead of the phenylacetyl in turrapubesol B. Additionally, a downshift ketone carbonyl [$\delta_C$ 216.9 (C-3)] together with two methylenes [$\delta_C$ 38.8 (C-1), 33.9 (C-2)] in its 1D NMR spectra suggested that the double bond between C-1 and C-2 in the skeleton of turrapubesol B has been hydrogenated. The locations of the acetyl group and the absent double bond were further established by observed HMBC and $^{1}$H-$^{1}$H COSY correlations. The relative stereochemistry of **5** was determined as the same with turrapubesol B by detailed analysis of its ROESY correlations together with comparison of their NMR data, and the $\beta$ configuration of H-7 was deduced by the correlation of H-7/Me-30 in the ROESY spectrum. Therefore, the structure of **5** was established as dysohainanin E.

Dysohainanin F (**6**), yellow powder, had the molecular formula of $C_{30}H_{48}O_3$ by the HREIMS [*m/z* 456.3616 [M]$^+$ (calcd. for 456.3603]. The $^{1}$H and $^{13}$C NMR data of **6** have a high similarity with 3$\beta$,25-dihydroxy-tirucalla-7,23-dien[7], only with the differences of C-3 and Me-26 in **6** oxidized to ketone carbonyl and hydroxymethyl, respectively, which was confirmed by the 2D NMR spectra. Compound **6** was determined and named as dysohainanin F.

## Experimental Section

**General Experimental Procedures**. Melting points were determined using an X-4 melting point apparatus (Yingyu Yuhua Apparatus Factory, Gongyi, China) and were not corrected. Optical rotations were determined with a Perkin-Elmer 241 polarimeter. IR spectra were recorded on a Bio-Rad FTS-135 spectrometer with a KBr disk. UV spectra were determined by Shimadzu UV2401PC. The $^{1}$H and $^{13}$C NMR spectra were recorded on a Bruker DRX-500 spectrometer, while 2D NMR spectra were recorded on Bruker Avance III

600 spectrometer. EIMS/ESIMS and HREIMS/HRESIMS spectra were measured with a Finnigan MAT 90 instrument and VG Auto Spec-3000 spectrometer, respectively. Preparative HPLC was performed on an Agilent column (i.d. 21.2 × 150 mm, XDB-C18, Agilent, USA), developed with $CH_3OH:H_2O$ or $CH_3CN:H_2O$ (flow rate: 25.0 mL/min, detection: UV 230 nm) at 25 °C. Column chromatography was performed on silica gel (90-150 $\mu$m; Qingdao Marine Chemical Inc.), MCI gel (CHP20P, 75-150 $\mu$m, Mitsubishi Chemical Industries Ltd.), C18 reversed-phase silica gel (20-45 $\mu$m; Merck, Darmstadt, Germany), and Sephadex LH-20 (40-70 $\mu$m; Amersham Pharmacia Biotech AB, Uppsala, Sweden). TLC plates were precoated with silica gel $GF_{254}$ and $HF_{254}$ (Qingdao Haiyang Chemical Plant, Qingdao, China).

**Plant Material.** The twigs and leaves of *D. hainanense* were collected in Xishuangbanna, Yunnan Province, People's Republic of China, and were identified by Prof. Xun Gong of Kunming Institute of Botany, Chinese Academy of Sciences (CAS). Voucher specimen (No. H20090901) was deposited in the State Key Laboratory of Phytochemistry and Plant Resources in West China, Kunming Institute of Botany, CAS, Kunming, China.

**Extraction and Isolation.** The powder of air-dried twigs and leaves of *D. hainanense* (24.0 kg) was extracted with 95% acetone (35 L × 3) under reflux. The extracts were combined and then suspended in water, which was extracted with petroleum ether (PE, 10 L ×3) and EtOAc (10 L × 3) respectively. The EtOAc part (450 g) were subjected to column chromatography over repeated silica gel, MCI gel, Sephadex LH-20 and preparative HPLC to afford **1** (1.5 mg), **2** (18.0 mg), **3** (17.0 mg), **4** (2.9 mg), **5** (17.0 mg), and **6** (11.0 mg). (see details in Electronic Supplementary Material)

**Dysohainanin A (1):** white powder; mp 145–147 °C; $[\alpha]_D^{11}$ –17.5 (*c* 0.12, $CHCl_3$); IR (KBr): $\nu_{max}$ 3439, 2957, 2924, 2853, 1738, 1462, 1138, 1072 $cm^{-1}$; for $^1H$ NMR and $^{13}C$ NMR, see Tables 1 and 2; positive ion HRESIMS *m/z* 725.3133 $[M + Na]^+$ (calcd. for $C_{37}H_{50}O_{13}Na$, 725.3149).

**Dysohainanin B (2):** colorless crystal; mp 159–161 °C; $[\alpha]_D^{17}$ +62.1 (*c* 0.32, $CHCl_3$); IR (KBr): $\nu_{max}$ 3447, 2968, 1739, 1177, 1143 $cm^{-1}$; for $^1H$ NMR and $^{13}C$ NMR, see Tables 1 and 2; positive ion HRESIMS: *m/z* 813.3309 $[M + Na]^+$ (calcd. for $C_{40}H_{54}O_{16}Na$, 813.3310).

**Dysohainanin C (3):** colorless crystal; mp 129–131 °C; $[\alpha]_D^{11}$ +24.1 (*c* 0.16, $CHCl_3$); IR (KBr): $\nu_{max}$ 3440, 2959, 2923, 1736, 1179, 1142 $cm^{-1}$; for $^1H$ NMR and $^{13}C$ NMR, see Tables 1 and 2; positive ion HRESIMS: *m/z* 827.3459 $[M + Na]^+$ (calcd. for $C_{41}H_{56}O_{16}Na$, 827.3466).

**Dysohainanin D (4):** colorless crystal; mp 174–175 °C; $[\alpha]_D^{11}$ –18.8 (*c* 0.08, $CHCl_3$); IR (KBr): $\nu_{max}$ 3448, 2957, 2923, 2853, 1743, 1462, 1137, 1077 $cm^{-1}$; for $^1H$ NMR and $^{13}C$ NMR, see Tables 1 and 2; positive ion HRESIMS: *m/z* 693.2871 $[M + Na]^+$ (calcd. for $C_{36}H_{46}O_{12}Na$, 693.2887).

**Dysohainanin E (5):** white powder; mp 88–90 °C; $[\alpha]_D^{17}$ –34.7 (*c* 0.26, $CHCl_3$); IR (KBr): $\nu_{max}$ 3433, 2932, 1707, 1378, 1247 $cm^{-1}$; for $^1H$ NMR and $^{13}C$ NMR, see Table 3; HREIMS *m/z* 530.3594 $[M]^+$ (calcd. for $C_{32}H_{50}O_6$, 530.3607).

**Dysohainanin F (6):** yellow powder; mp 82–84 °C; $[\alpha]_D^{17}$ –28.7 (*c* 0.38, $CHCl_3$); IR (KBr): $\nu_{max}$ 3438, 2966, 2931, 1708, 1460, 1385, 755 $cm^{-1}$; for $^1H$ NMR and $^{13}C$ NMR, see Table 3; HREIMS *m/z* 456.3616 $[M]^+$ (calcd. for $C_{30}H_{48}O_3$, 456.3603).

**Cytotoxicity Assay.** The anti-tumor activity of **1**, **5**, **6**, **10**, **11**, and **13** against HL-60, SMMC-7721, A-549, MCF-7 and SW480 cell lines was determined by the MTT method[18], compound **13** showed *in vitro* cytotoxicity against HL-60, SMMC-7721, A-549, MCF-7, and SW480 cell lines, with $IC_{50}$ values of 24.3, > 40, 28.1, 30.7, and 22.5 $\mu$M, and the acetone crude extraction of *D. hainanense* with $IC_{50}$ values of 36.9, 40.6, 60.7, 44.0, and 36.7 $\mu$g/mL, respectively, while compounds **1**, **5**, **6**, **10**, and **11** have not shown *in vitro* cytotoxicity against these cell lines. (see details in Table 4).

**Table 4. Cytotoxicities (for compounds and extract $IC_{50}$ in $\mu$M and $\mu$g/mL respectively) of compounds 1, 5, 6, 10, 11, 13, and the acetone extract of *D. hainanense*.**

| Samples | HL-60 | SMMC-7721 | A-549 | MCF-7 | SW480 |
|---|---|---|---|---|---|
| **1** | > 40 | > 40 | > 40 | > 40 | > 40 |
| **5** | > 40 | > 40 | > 40 | > 40 | > 40 |
| **6** | > 40 | > 40 | > 40 | > 40 | > 40 |
| **10** | > 40 | > 40 | > 40 | > 40 | > 40 |
| **11** | > 40 | > 40 | > 40 | > 40 | 23.5 |
| **13** | 24.3 | > 40 | 28.1 | 30.7 | 22.5 |
| extract | 36.9 | 40.6 | 60.7 | 44.0 | 36.7 |
| *cis*-platin | 2.51 | 15.0 | 13.6 | 10.6 | 12.3 |
| taxol | < 0.008 | < 0.008 | < 0.008 | < 0.008 | < 0.008 |

**Insecticidal Assay.** Compounds **2** and **3** have been test their insecticidal activity using brine shrimp at the concerntrations of 100, 50, 10 ppm. The result exbihited that they were inactive toward brine shrimp.

**Method for Insecticidal Test[19].** The test compounds were dissolved in DMSO or water and then diluted with artificial seawater to the final concentrations of 100, 50, 10 ppm (mg/L), which were added to 96-well plates with each well of 15–25 brine shrimps. After cultivation under 28 °C for 24 h, the numbers of the dead brine shrimps were counted with a microscope. Each concentration was repeated in triplicate, and the control group was treated in the same way without samples.

Mortality = (the mortality of the brine shrimps with sample – the mortality of the brine shrimps of control group) / (1 – the mortality of the brine shrimps of control group) × 100%

### Electronic Supplementary Material

Supplementary material is available in the online version of this article at http://dx.doi.org/10.1007/s13659-011-0030-8 and is accessible for authorized users.

**Acknowledgments**

This work was supported by grants from the Ministry of Science and Technology (2009CB522300 and 2009CB940900).

**Open Access** This article is distributed under the terms of the Creative Commons Attribution License which permits any use, distribution, and reproduction in any medium, provided the original author(s) and source are credited.

## Electronic supplementary material

Supplementary material, approximately 12.2 MB.
