# Supplementary material for: Limonoids and triterpenoids from the twigs and leaves of Dysoxylum hainanense
Source: Nat Prod Bioprospect. 2012 Feb 22;2(1):29–34. doi: 10.1007/s13659-011-0030-8 (PMC4131571; doi:10.1007/s13659-011-0030-8)
Supplement: Supplementary file 1 — Supplementary material, approximately 12.2 MB. [file 13659_2011_30_MOESM1_ESM.pdf]

Electronic Supplementary Material

Limonoids and triterpenoids from the twigs and leaves of *Dysoxylum hainanense*

Wen-Xing LIU,[a,b] Gui-Hua TANG,[a,b] Hong-Ping HE,[a] Yu ZHANG,[a] Shun-Lin LI,[a,]* and Xiao-Jiang HAO[a,]*

[a]State Key Laboratory of Phytochemistry and Plant Resources in West China, Kunming Institute of Botany, Chinese Academy of Sciences, Kunming 650201, China

[b]Graduate University of Chinese Academy of Sciences, Beijing 100049, China

Received 9 November 2011; Accepted 13 February 2011

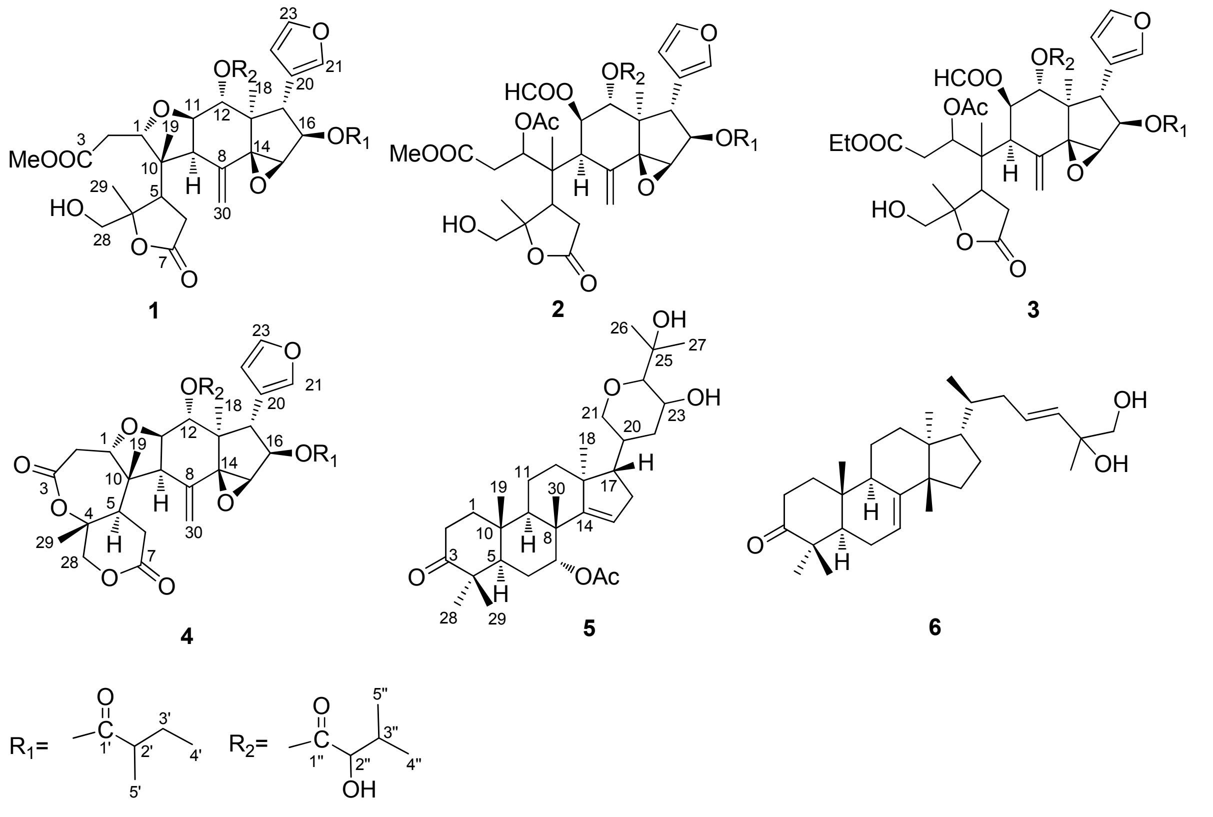

Structures of compounds **1**–**6**.

*To whom correspondence should be addressed. E-mail: haoxj@mail.kib.ac.cn; lisl@mail.kib.ac.cn

General experimental procedures

All the mps were obtained on an X-4 micromelting apparatus and were uncorrected. Optical rotations were determined with a Perkin-Elmer 241 polarimeter. IR spectra were recorded on a Bio-Rad FTS-135 spectrometer with a KBr disk. UV spectra were determined by Shimadzu UV2401PC. The ^{1}H and ^{13}C NMR spectra were recorded on a Bruker DRX-500 spectrometer, while 2D NMR spectra were recorded on Avance III 600 spectrometer. EIMS/ESIMS and HREIMS/HRESIMS spectra were measured with a Finnigan MAT 90 instrument and VG Auto Spec-3000 spectrometer, respectively. Preparative HPLC was performed on an Agilent column (i.d. 21.2 × 150 mm, XDB-C18, Agilent, USA), developed with CH_3OH: H_2O or CH_3CN:H_2O (flow rate: 25.0 mL/min, detection: UV 230 nm) at 25 °C. Column chromatography was performed on silica gel (90-150 μm; Qingdao Marine Chemical Inc.), MCI gel (CHP20P, 75-150 μm, Mitsubishi Chemical Industries Ltd.), C18 reversed-phase silica gel (20-45μm; Merck, Darmstadt, Germany), and Sephadex LH-20 (40-70 μm; Amersham Pharmacia Biotech AB, Uppsala, Sweden). TLC plates were precoated with silica gel GF_{254} and HF_{254} (Qingdao Haiyang Chemical Plant, Qingdao, People's Republic of China).

Plant material

The twigs and leaves of *Dysoxylum hainanense* were collected in Xishuangbanna, Yunnan Province, People's Republic of China, and were identified by Prof. Xun Gong of Kunming Institute of Botany, Chinese Academy of Sciences. Voucher specimen (No. H20090901) was deposited in State Key Laboratory of Phytochemistry and Plant Resources in West China, Kunming Institute of Botany, Chinese Academy of Sciences, Kunming, China.

Extraction and isolation

The powder of air-dried twigs and leaves of *D. hainanense* (24.0 kg) was extracted with 95% acetone (35 L × 3) under reflux. The extracts were combined and then suspended in water, which was extracted with petroleum ether (PE, 10 L ×3) and EtOAc (10 L × 3) respectively. The EtOAc extracts (450 g) were subjected to silica gel column chromatograph (dimension of the column: 20 cm; length: 50 cm), eluted with petroleum ether: Acetone [100: 0 (15 L×3), 80: 20 (15L×3), 60: 40 (25 L×3), 40: 60 (25 L×3), 20: 80 (15 L×3), 0: 100 (15 L×3)], yielding nine fractions Fr_1-Fr_9. Fr_5 (20.0 g) was eluted with CH_3OH: H_2O [50: 50 (4 L), 70: 30 (10 L), 85: 15 (4 L), 100: 0 (5 L), flow rate: 20 ml/min] in MCI (dimension of the column:5 cm; length: 45 cm) and then in RP18 [dimension of the column: 5 cm; length: 45 cm, mobile phase: CH_3OH: H_2O (40: 60 (6 L), 50: 50 (8L), 60: 40 (10 L), 70: 30 (10 L), 75: 25 (5 L), 85: 15 (4 L), 100: 0 (8 L)], to obtain five fractions A_1-A_5, fraction A_1 (2.0 g) was subjected by Sephadex LH-20 (dimension of column: 2 cm; length: 150 cm) with the solvent of MeOH (1.0 L) to yield four fractions B_1-B_4, B_1 (430 mg) subjected by silica gel column (dimension: 3.5 cm; length: 30 cm) eluted with $CHCl_3$: MeOH (100:1, 3 L) to afford **2** (18 mg); A_5 (2.5 g) was subjected to Sephadex LH-20 (dimension of column: 2 cm;

length: 150 cm) eluted with MeOH (1.0 L) to get three fractions C_1-C_3, C_1 (1.5 g) was eluted by silica gel column (dimension: 3 cm; length: 30 cm) with chloroform: acetone (11:1, 3 L) and give rise to seven fractions D_1- D_7, D_4 (70 mg) was subjected by silica gel column (dimension: 1.5 cm; length: 16 cm) with chloroform: acetone (100:4, 1.5 L) and obtained **4** (2.9 mg), respectively, D_5 (40 mg) and D_6 (60 mg) were subjected by HPLC eluted with 70 % and 60% MeOH, afford **3** (17 mg, retention time: 8.5 min) and **1** (1.5 mg, retention time: 21.0 min), respectively. Fr_4 (29.8 g) was subjected by CH_3OH: H_2O [50: 50 (4 L), 70: 30 (10 L), 85: 15 (4 L), 100: 0 (5 L), flow rate: 20 ml/min] in MCI (dimension of column:5 cm; length: 45 cm) and then in RP18 [dimension of column:5 cm; length: 45 cm, mobile phase: CH_3OH: H_2O (40: 60 (6 L), 50: 50 (8L), 60: 40 (10 L), 70: 30 (10 L), 75: 25 (5 L), 85: 15 (4 L), 100: 0 (8 L)], to afford seven fractions E_1- E_7, E_1 (1.8 g) was eluted with MeOH in Sephadex LH-20 (dimension of column: 2 cm; length: 150 cm) with the solvent of MeOH (1.0 L) to afford F_1 –F_5, F_5 (800 mg) was subjected by RP18 [dimension of column:1 cm; length: 45 cm, mobile phase: CH_3OH: H_2O (40 : 60 (2 L), 50 : 50 (2 L), 60 : 40 (3 L), 70 : 30 (3 L), 75 : 25 (3 L), 85 : 15 (2 L), 100 : 0 (3 L)], three fractions were obtained as G_1-G_3. G_3 (290 mg) was subjected by chloroform: acetone (45:1, 1.0 L) in silica gel column (dimension: 3 cm, length 20 cm) to afford two fractions H_1, H_2, and then H_2 (50 mg) was eluted by silica gel column (dimension: 1 cm, length 8 cm) with chloroform: acetone (100:1, 0.3 L) and obtained **6** (11 mg). E_3 (1.1g) was subjected with Sephadex LH-20 (dimension: 2 cm; length: 150 cm) with the solvent of MeOH (1.0 L) to afford two fractions I_1 and I_2, I_2 (800 mg) was subjected by silica gel column (dimension: 4 cm, length 20 cm) with chloroform: acetone (35:1, 2.0 L) to afford J_1 and J_2. J_2 (50 mg) was finally subjected by HPLC with 50% of acetonitrile (retention time: 8.0 min), 17.0 mg of **5** was obtained.

Purity of compounds

Purity of compounds **1–13**: >95% (based on their ^{1}H-NMR spectra)

Figure Legends

Fig. 1S ^{1}H NMR spectra of 1 6
Fig. 2S ^{13}C NMR spectra of 1 7
Fig. 3S HSQC spectra of 1 8
Fig. 4S HMBC spectra of 1 9
Fig. 5S COSY spectra of 1 10
Fig. 6S ROESY spectra of 1 11
Fig. 7S HRMS spectra of 1 12
Fig. 8S IR spectra of 1 13
Fig. 9S Optical rotation spectra of 1 14
Fig. 10S ^{1}H NMR spectra of 2 15
Fig. 11S ^{13}C NMR spectra of 2 16
Fig. 12S HSQC spectra of 2 17
Fig. 13S HMBC spectra of 2 18
Fig. 14S COSY spectra of 2 19
Fig. 15S ROESY spectra of 2 20
Fig. 16S HRMS spectra of 2 21
Fig. 17S IR spectra of 2 22
Fig. 18S Optical rotation spectra of 2 23
Fig. 19S ^{1}H NMR spectra of 3 24
Fig. 20S ^{13}C NMR spectra of 3 25
Fig. 21S HSQC spectra of 3 26
Fig. 22S HMBC spectra of 3 27
Fig. 23S COSY spectra of 3 28
Fig. 24S HRMS spectra of 3 29
Fig. 25S IR spectra of 3 30
Fig. 26S Optical rotation spectra of 3 31
Fig. 27S ^{1}H NMR spectra of 4 32
Fig. 28S ^{13}C NMR spectra of 4 33
Fig. 29S HSQC spectra of 4 34
Fig. 30S HMBC spectra of 4 35
Fig. 31S COSY spectra of 4 36
Fig. 32S COSY spectra of 4 37
Fig. 33S HRMS spectra of 4 38
Fig. 34S IR spectra of 4 39
Fig. 35S Optical rotation spectra of 4 40
Fig. 36S ^{1}H NMR spectra of 5 41
Fig. 37S ^{13}C NMR spectra of 5 42
Fig. 38S HSQC spectra of 5 43
Fig. 39S HMBC spectra of 5 44
Fig. 40S COSY spectra of 5 45
Fig. 41S HRMS spectra of 5 46
Fig. 42S IR spectra of 5 47
Fig. 43S Optical rotation spectra of 5 48
Fig. 44S ^{1}H NMR spectra of 6 49
Fig. 45S ^{13}C NMR spectra of 6 50
Fig. 46S HSQC spectra of 6 51

Fig. 47S HMBC spectra of 6 52
Fig. 48S COSY spectra of 6 53
Fig. 49S HRMS spectra of 6 54
Fig. 50S IR spectra of 6 55
Fig. 51S Optical rotation spectra of 6 56
Fig. 52S Selected COSY correlations and HMBC correlations of 1-6: 56

Fig. 1S ^{1}H NMR spectra of 1

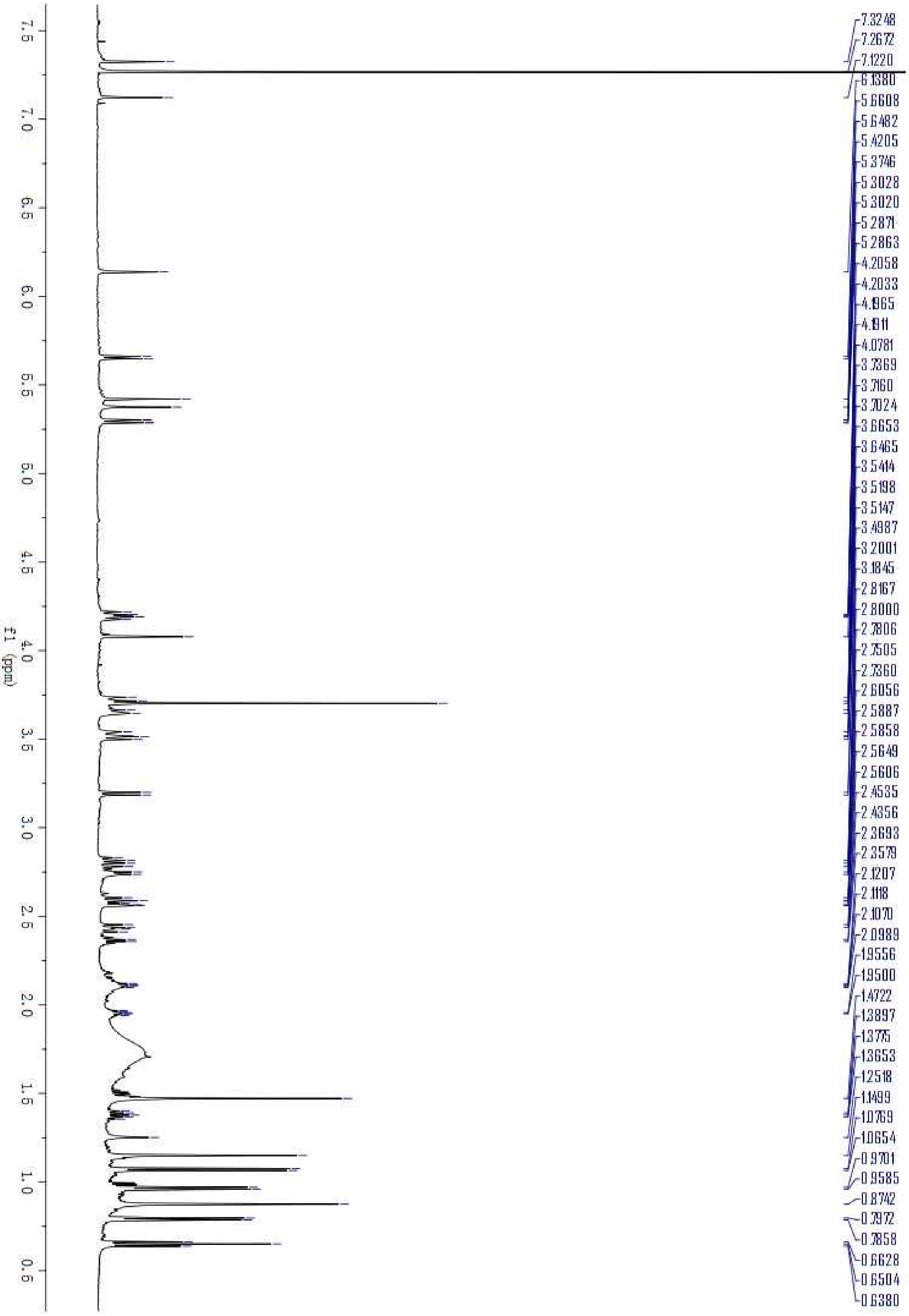

Fig. 2S ^{13}C NMR spectra of 1

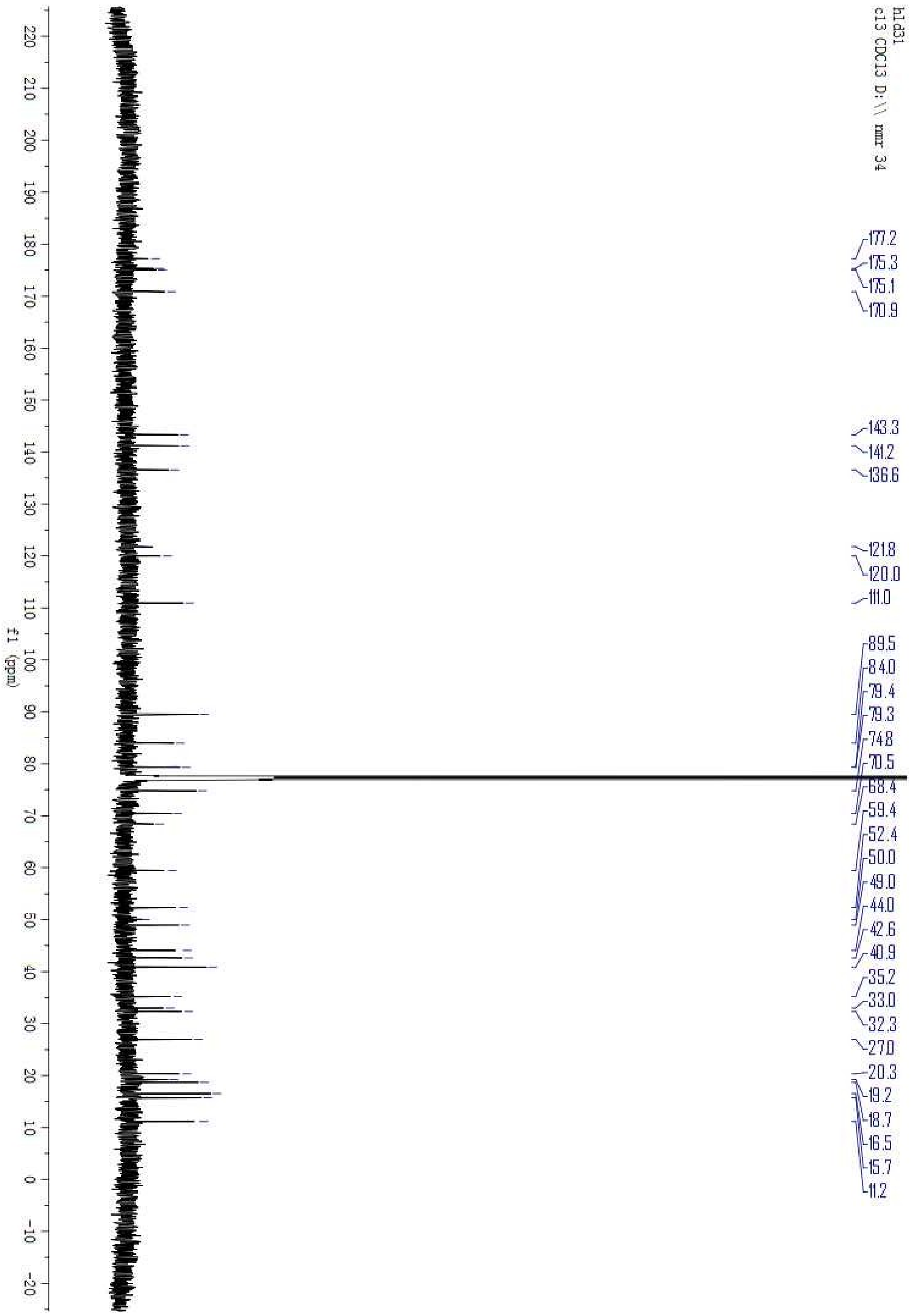

Fig. 3S HSQC spectra of 1

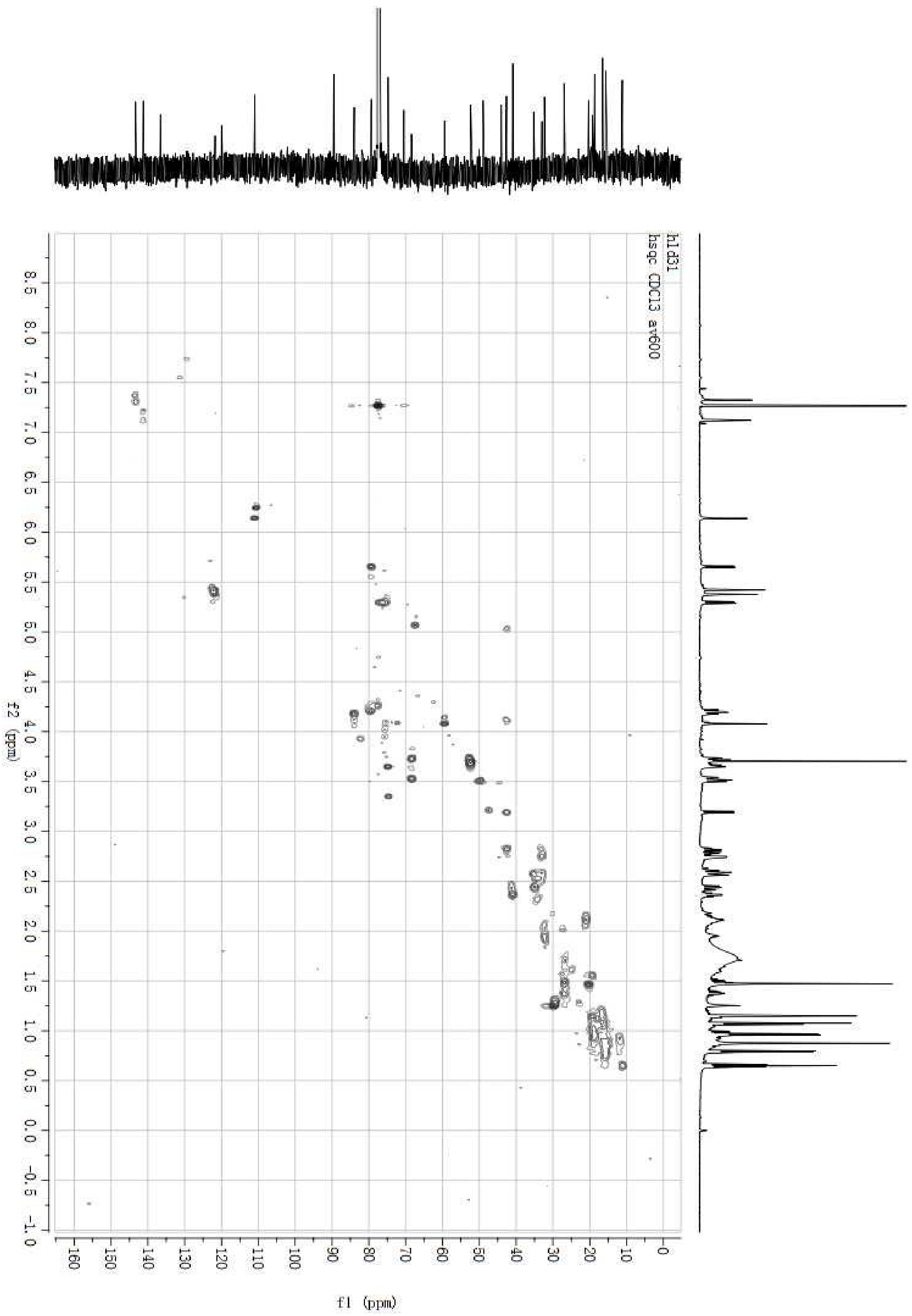

Fig. 4S HMBC spectra of 1

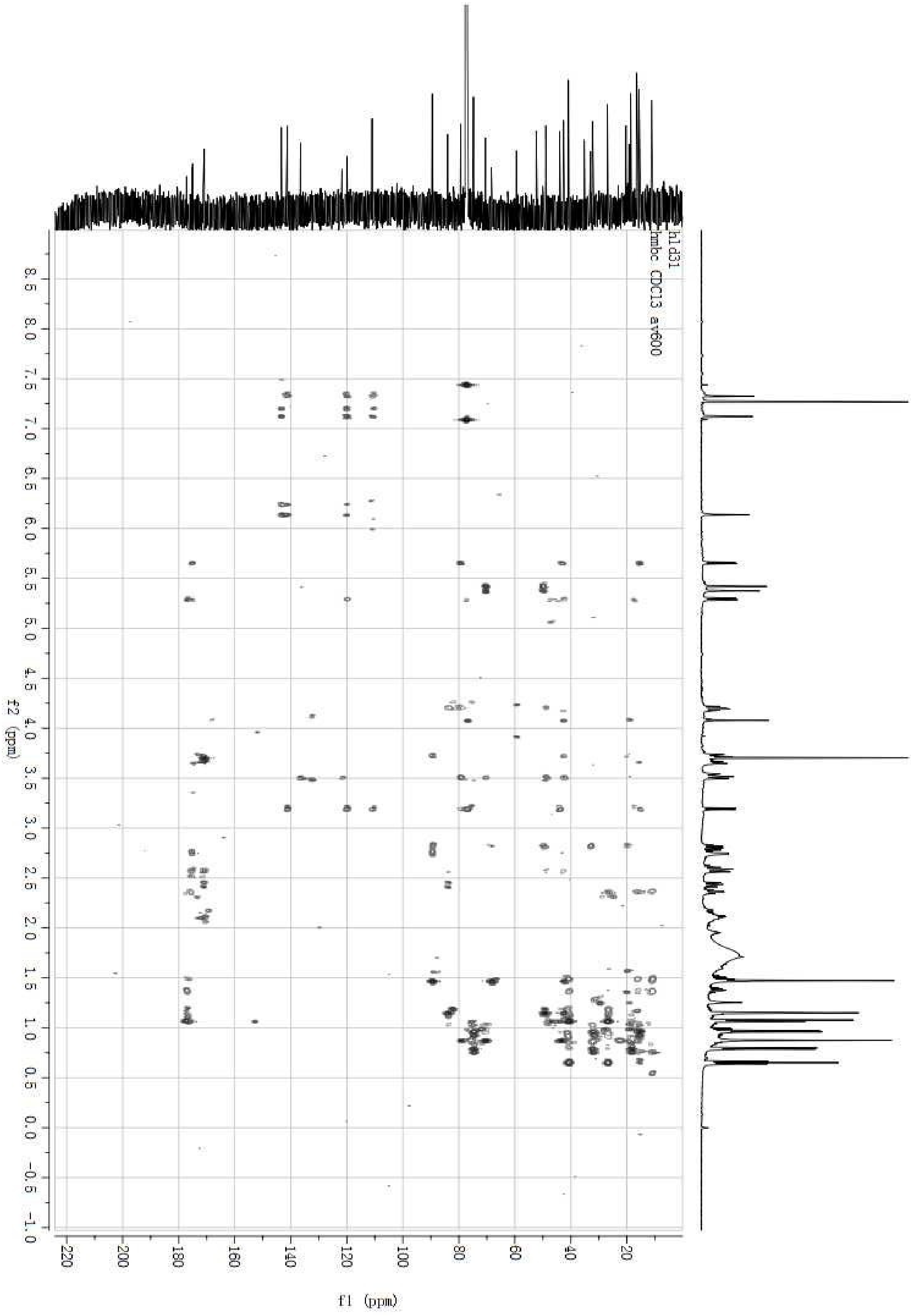

Fig. 5S COSY spectra of 1

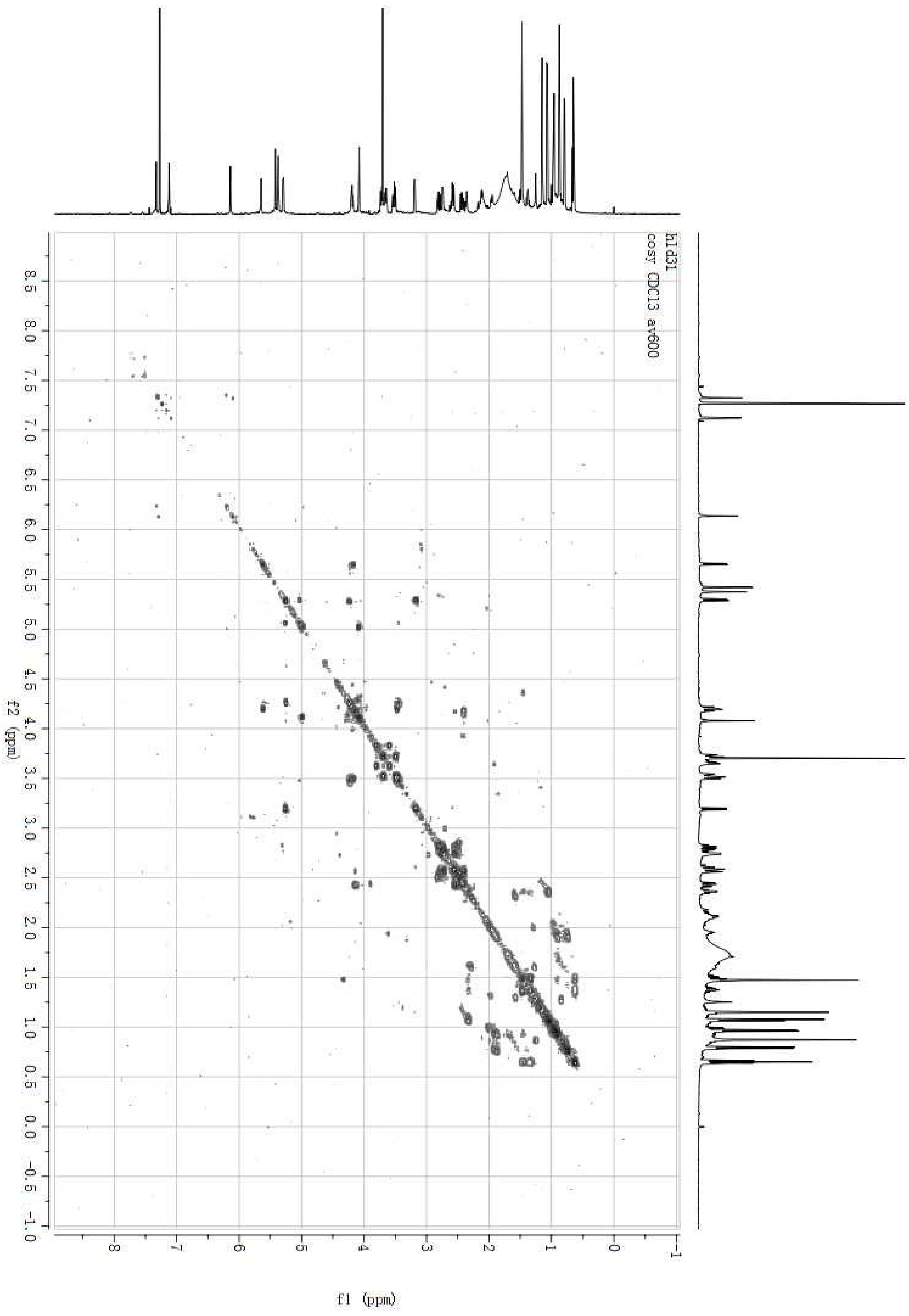

Fig. 6S ROESY spectra of 1

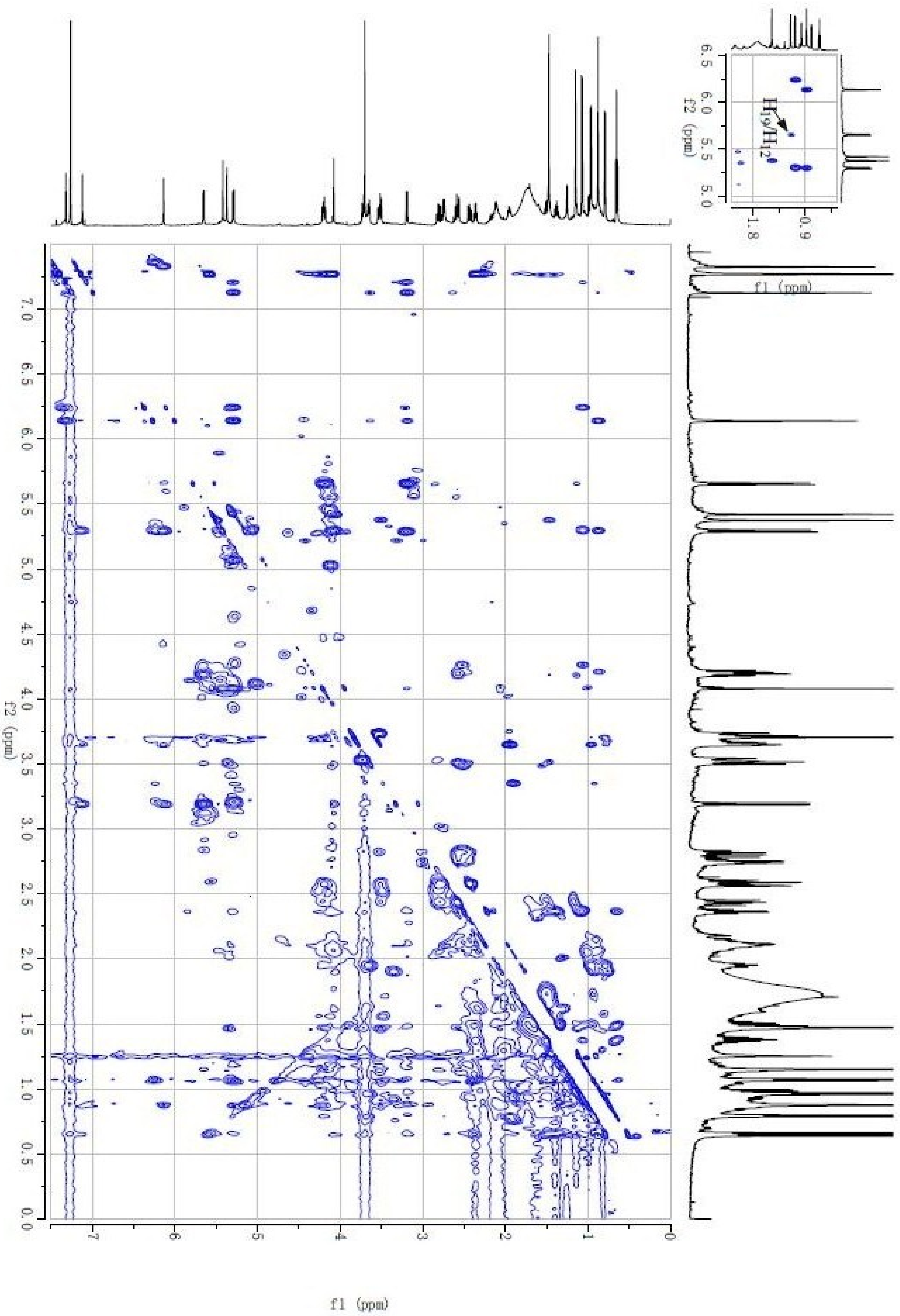

Fig. 7S HRMS spectra of 1

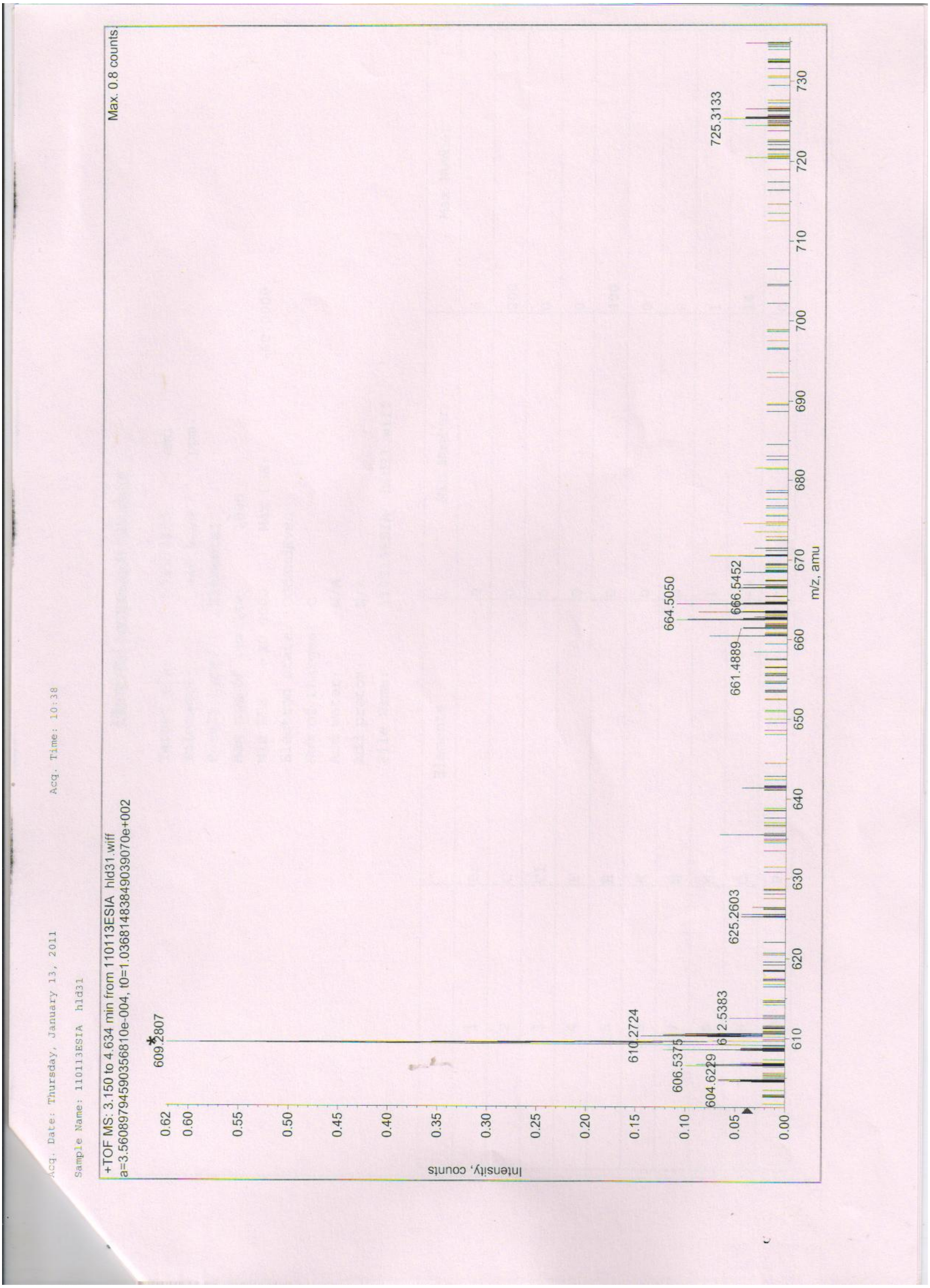

Fig. 8S IR spectra of 1

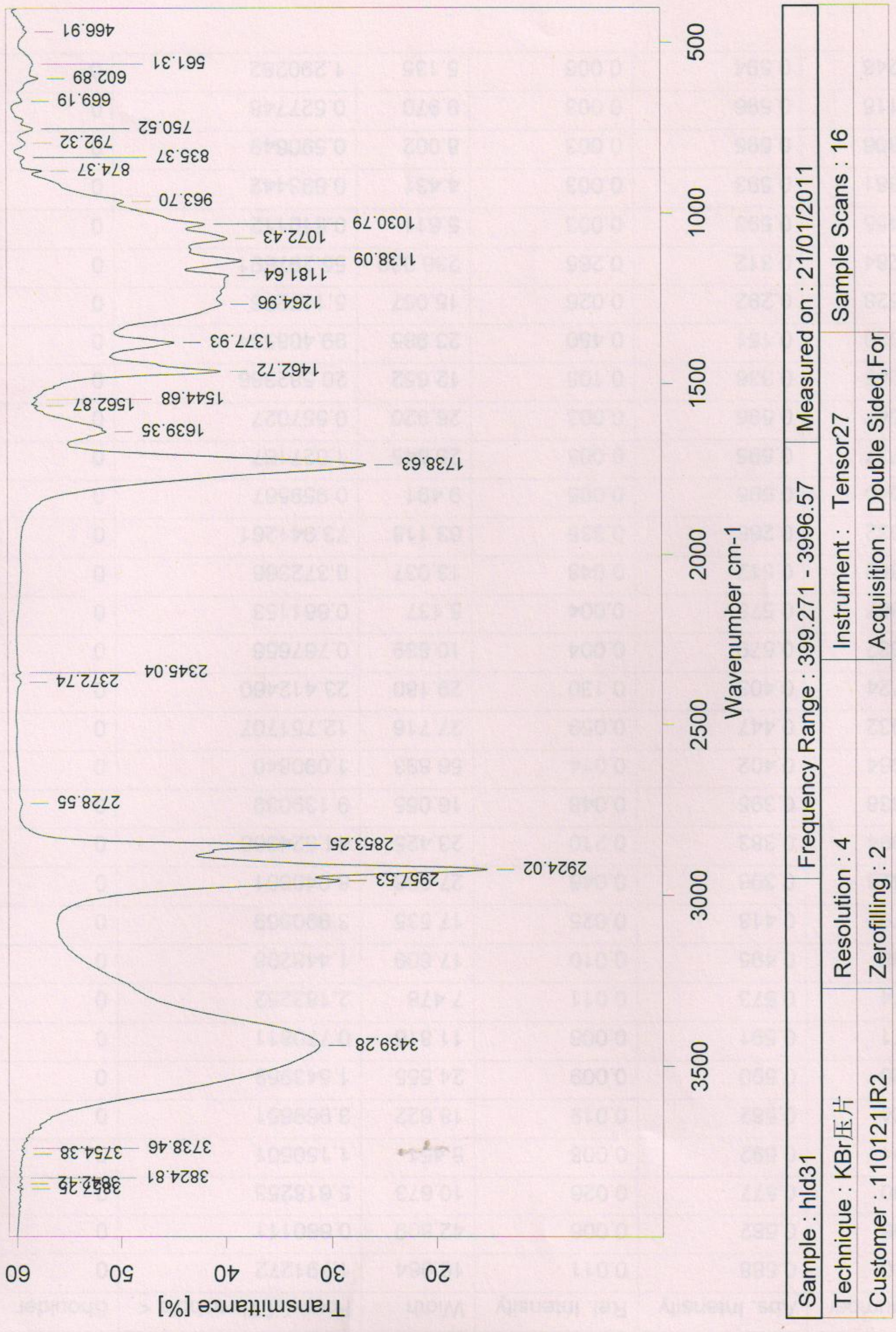

Fig. 9S Optical rotation spectra of 1

Optical rotation measurement

Model : P-1020 (A060460638)

No.	Sample	Mode	Data	Monitor<br>Blank	Temp.<br>Cell<br>Temp Point	Date<br>Comment<br>Sample Name	Light<br>Filter<br>Operator	Cycle Time<br>Integ Time
No.1	18 (1/3)	Sp.Rot	-17.8330	-0.0107<br>0.0000	11.7<br>50.00<br>Cell	Fri Jan 14 10:39:37 2011<br>0.00120g/mlCHCl3<br>HLD31	Na<br>589nm	2 sec<br>10 sec
No.2	18 (2/3)	Sp.Rot	-19.5000	-0.0117<br>0.0000	11.7<br>50.00<br>Cell	Fri Jan 14 10:39:50 2011<br>0.00120g/mlCHCl3<br>HLD31	Na<br>589nm	2 sec<br>10 sec
No.3	18 (3/3)	Sp.Rot	-15.3330	-0.0092<br>0.0000	11.7<br>50.00<br>Cell	Fri Jan 14 10:40:04 2011<br>0.00120g/mlCHCl3<br>HLD31	Na<br>589nm	2 sec<br>10 sec

-17.5556°

Fig. 10S ^{1}H NMR spectra of 2

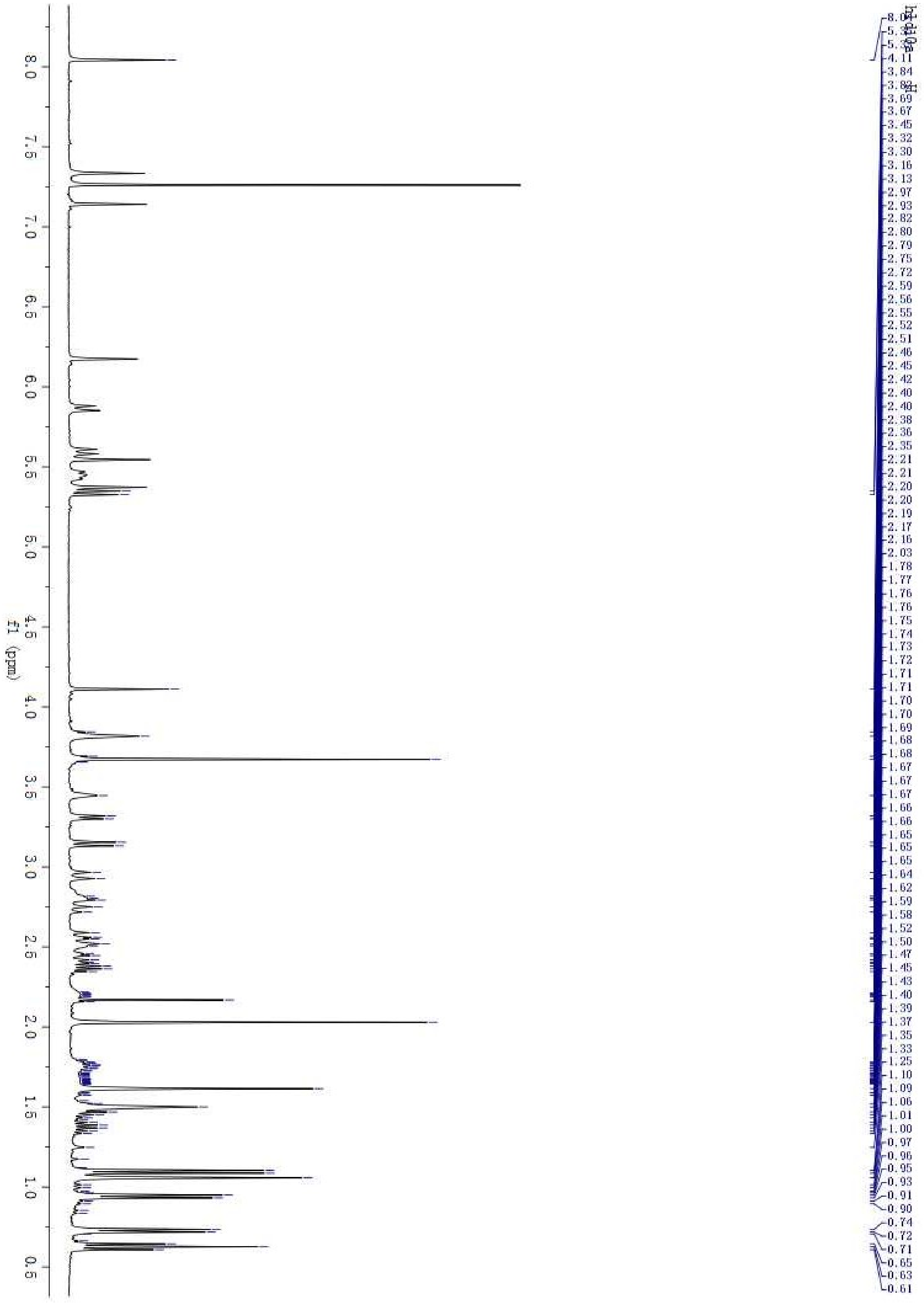

Fig. 11S ^{13}C NMR spectra of 2

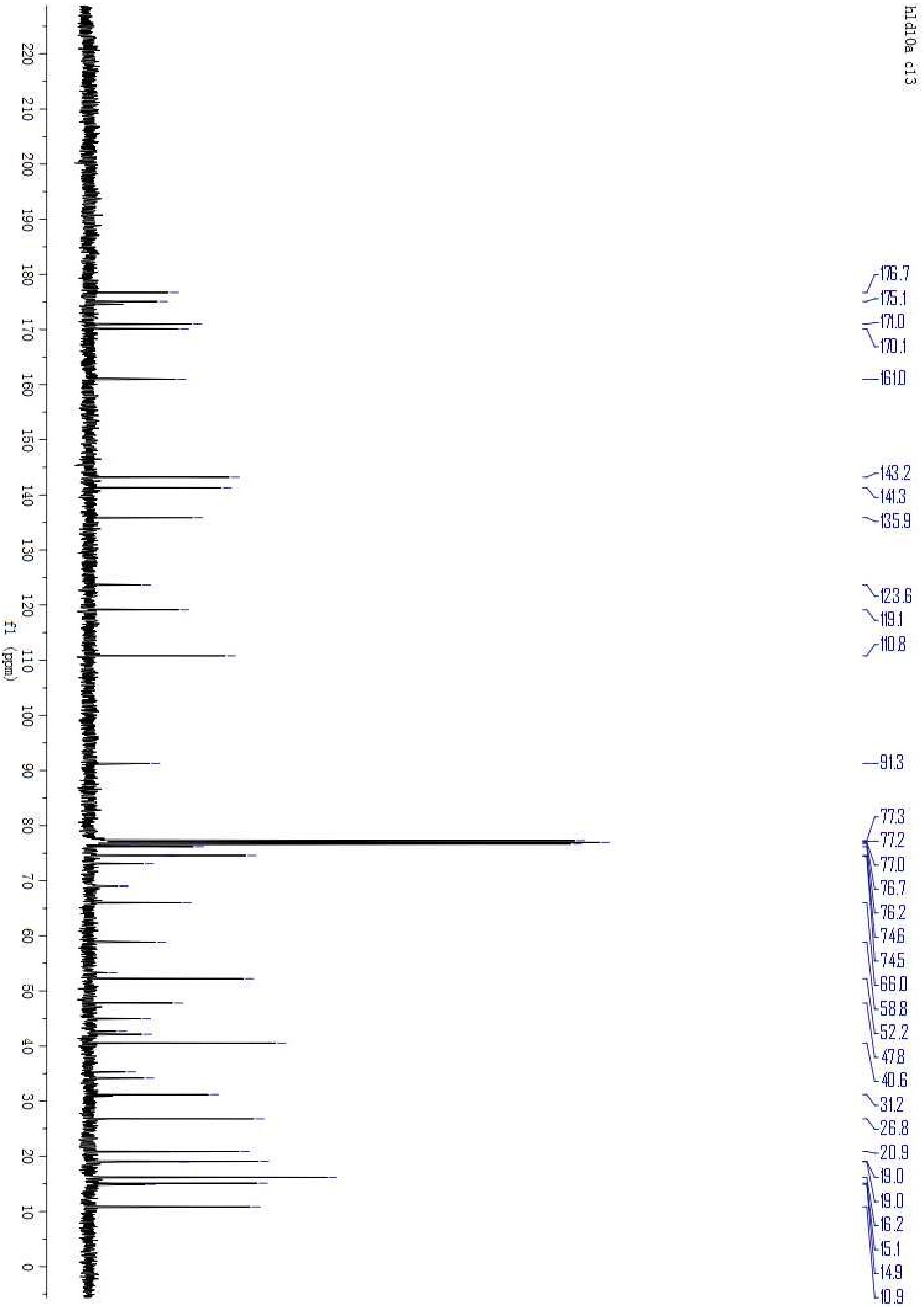

Fig. 12S HSQC spectra of 2

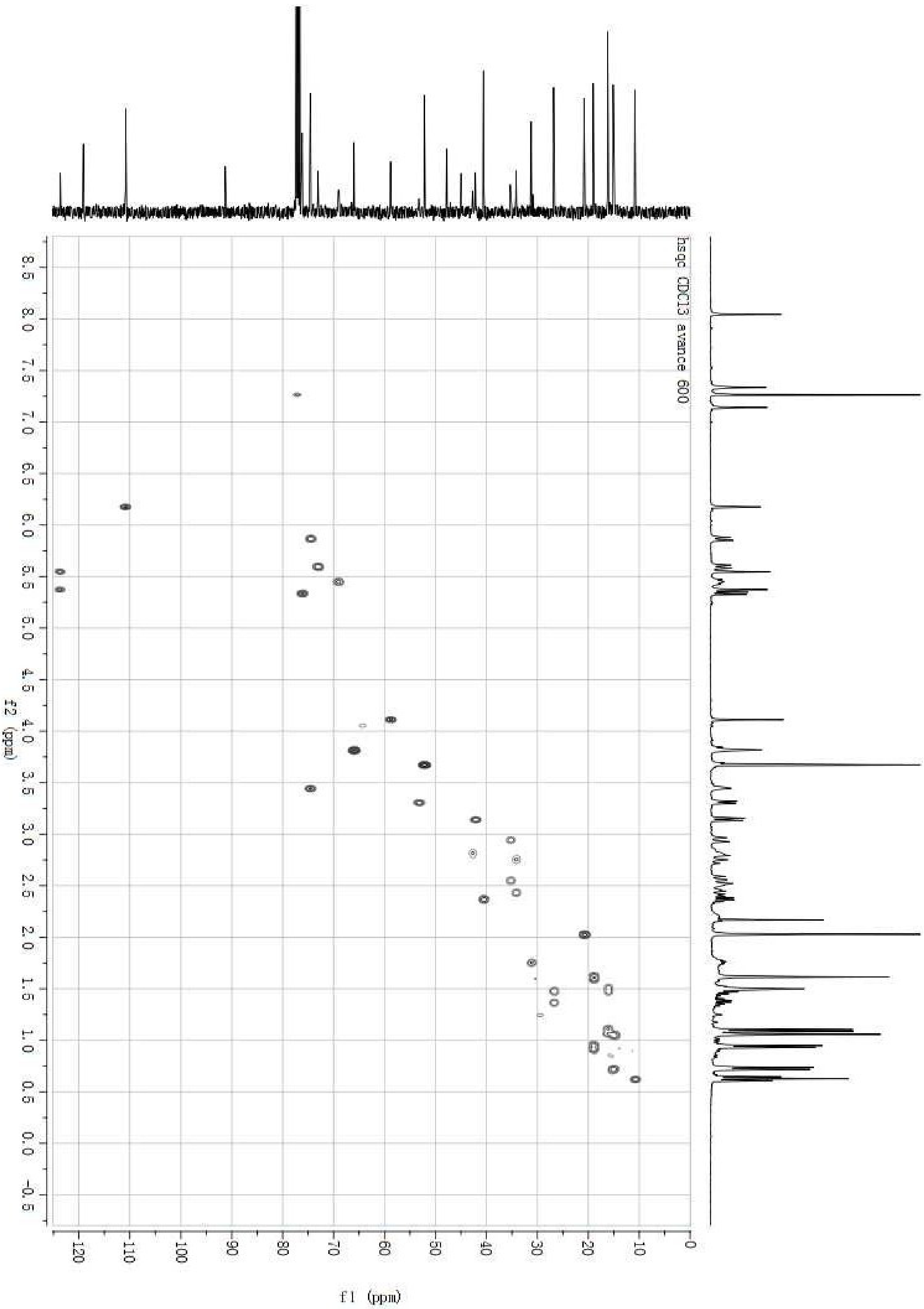

Fig. 13S HMBC spectra of 2

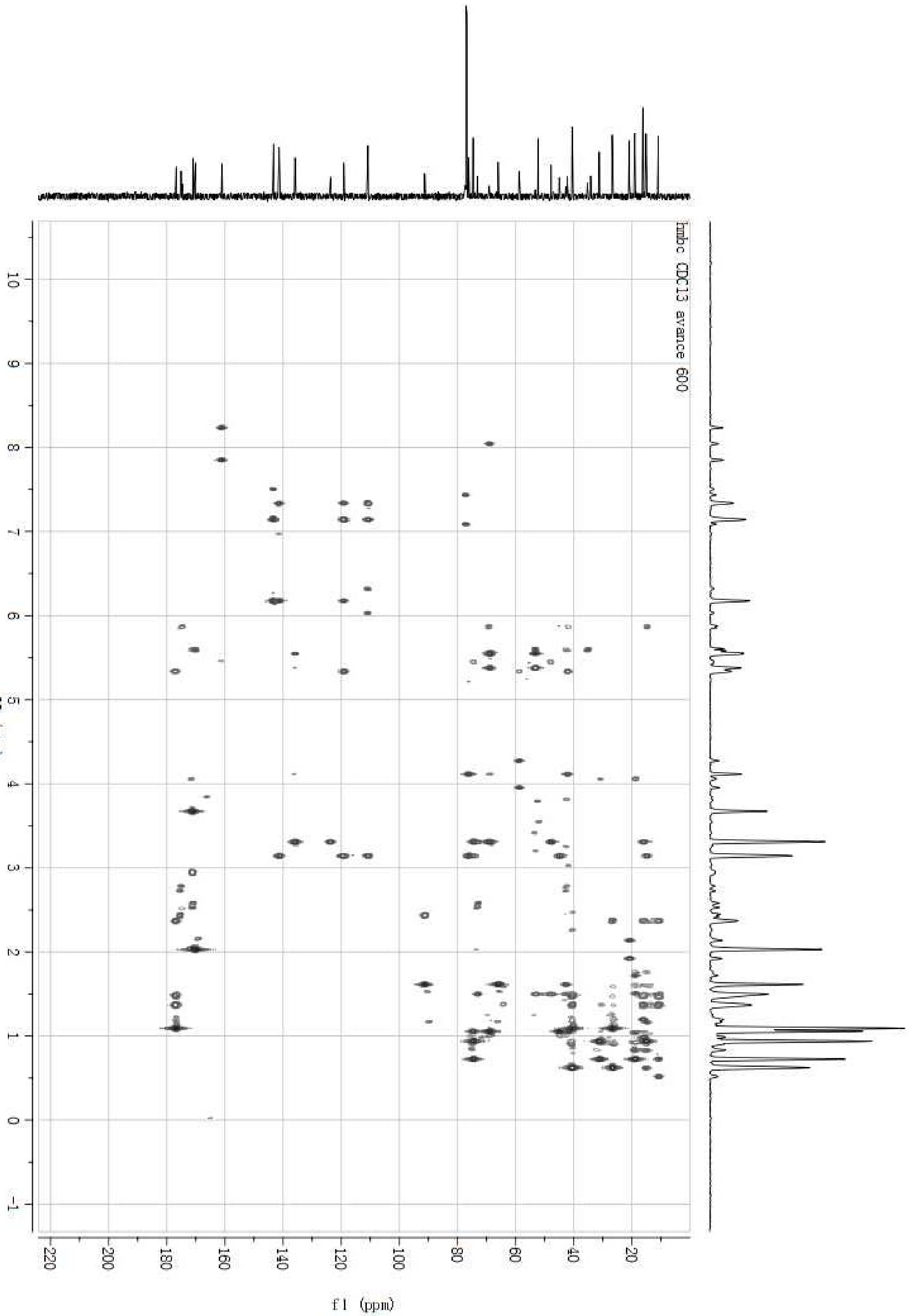

Fig. 14S COSY spectra of 2

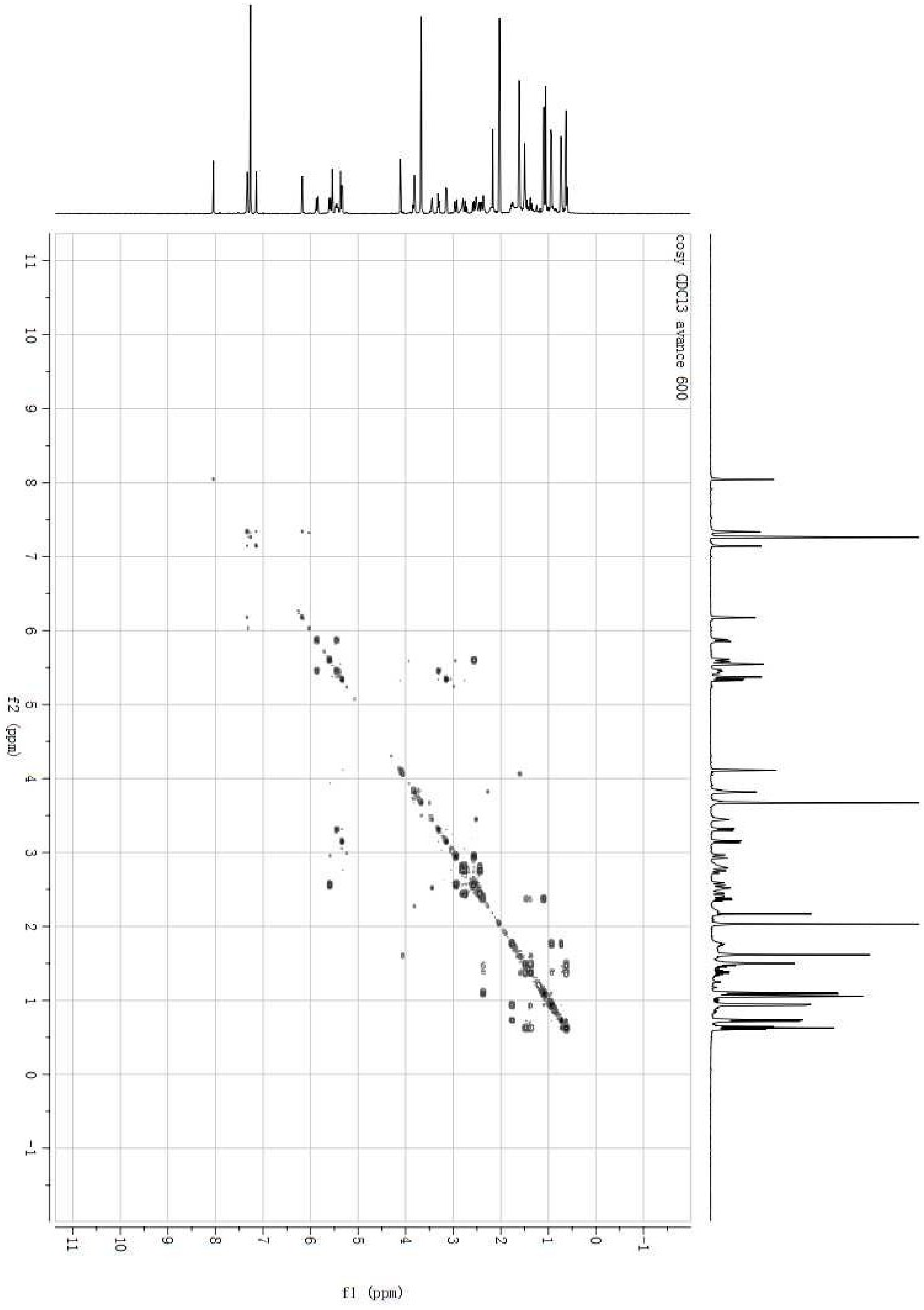

Fig. 15S ROESY spectra of 2

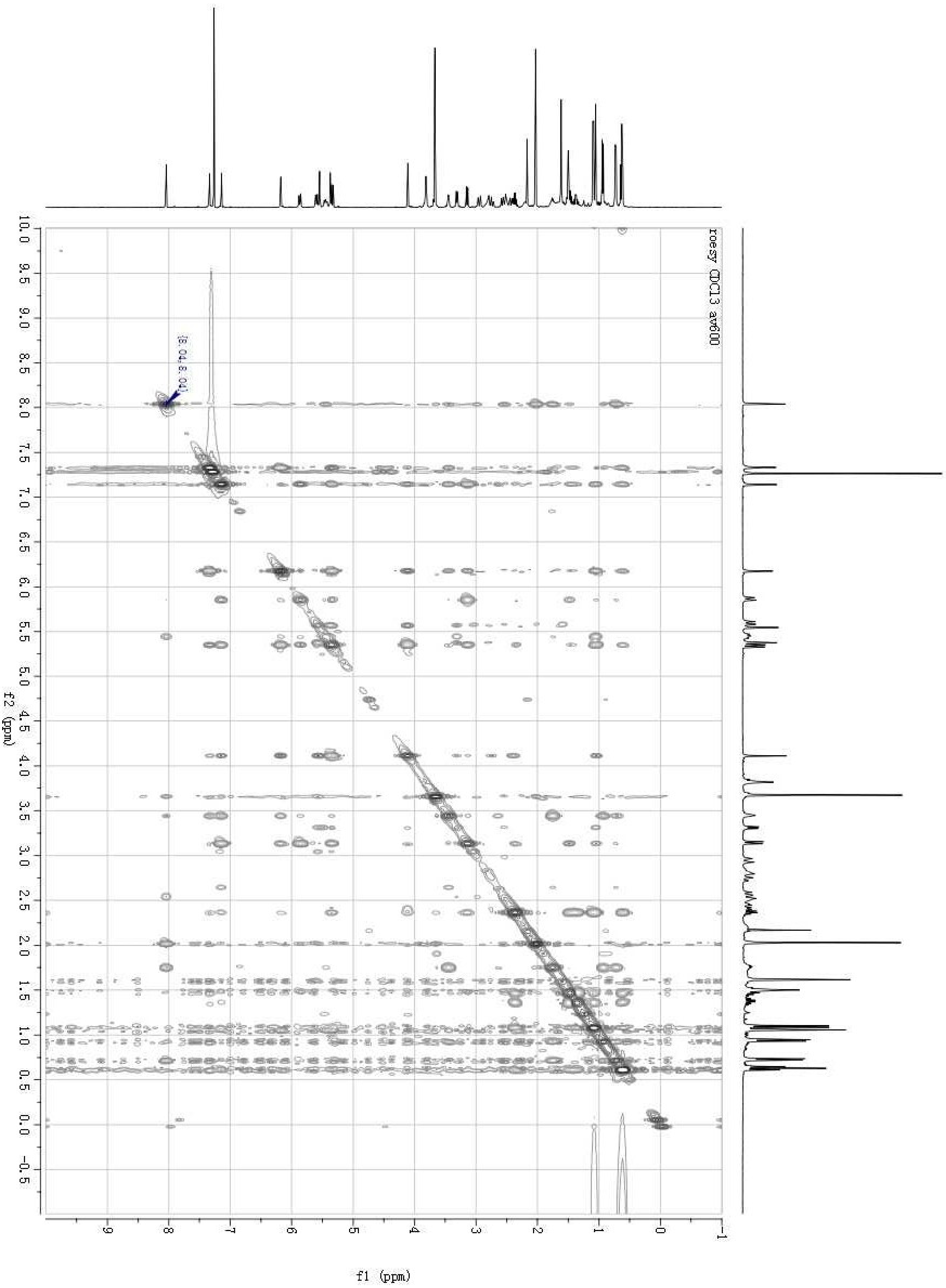

Fig. 16S HRMS spectra of 2

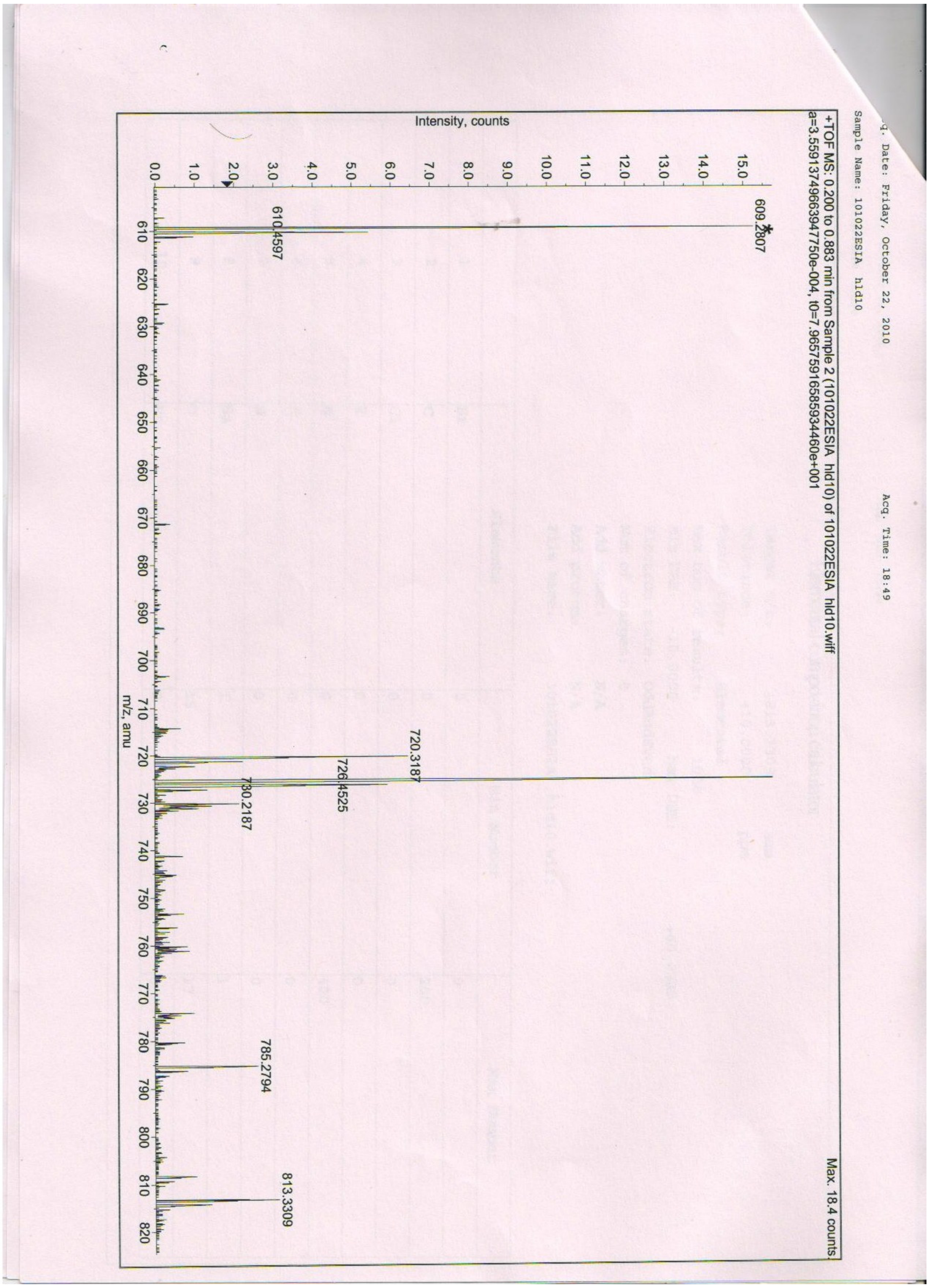

Fig. 17S IR spectra of 2

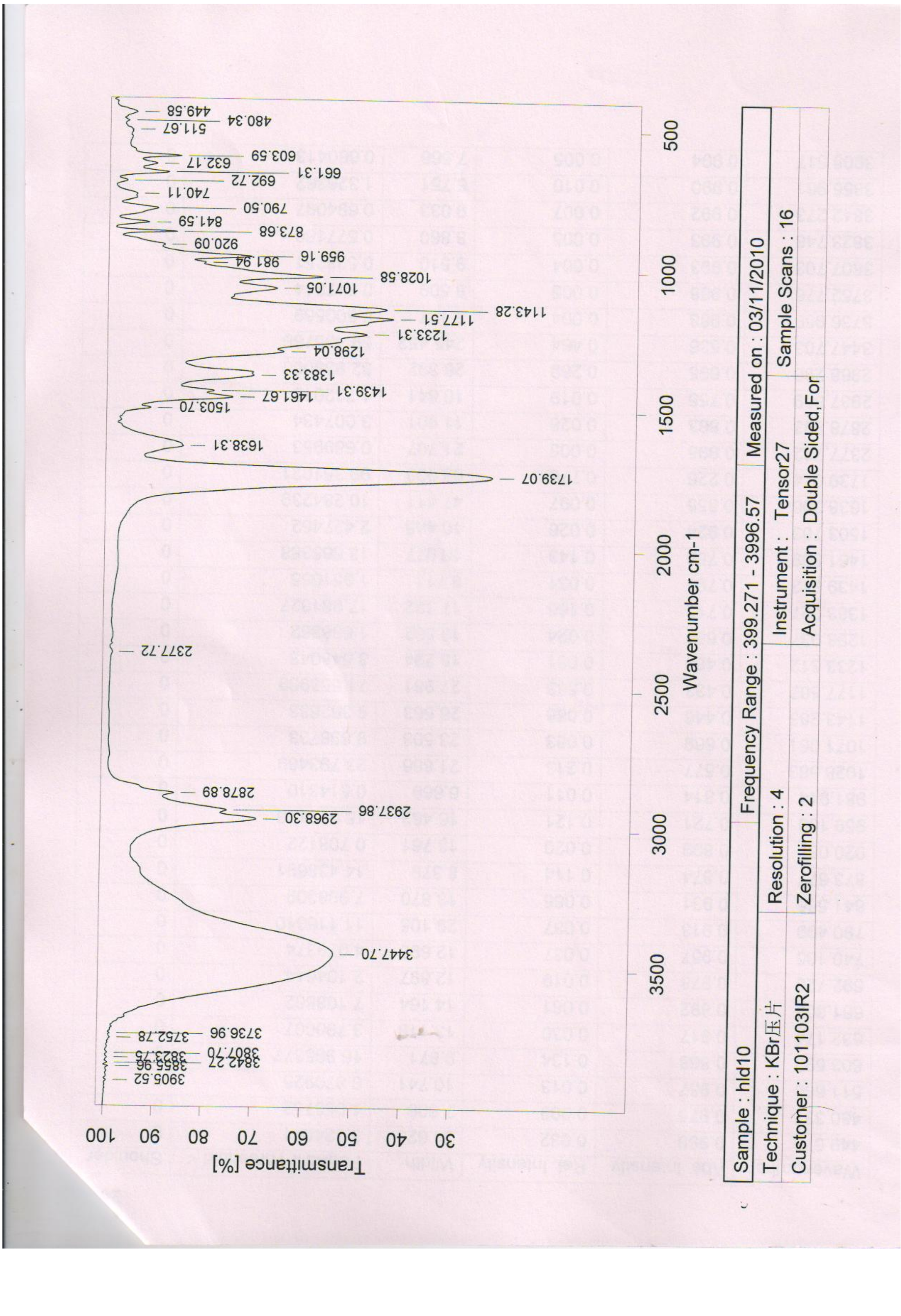

Fig. 18S Optical rotation spectra of 2

Optical rotation measurement

Model : P-1020 (A060460638)

No.	Sample	Mode	Data	Monitor<br>Blank	Temp.<br>Cell<br>Temp Point	Date<br>Comment<br>Sample Name	Light<br>Filter<br>Operator	Cycle Time<br>Integ Time
No.1	11 (1/3)	Sp.Rot	62.4620	0.1015<br>0.0000	17.3<br>50.00<br>Cell	Tue Nov 02 14:14:03 2010<br>0.00325g/mlCHCl3<br>HLD10	Na<br>589nm	2 sec<br>10 sec
No.2	11 (2/3)	Sp.Rot	61.9690	0.1007<br>0.0000	17.3<br>50.00<br>Cell	Tue Nov 02 14:14:16 2010<br>0.00325g/mlCHCl3<br>HLD10	Na<br>589nm	2 sec<br>10 sec
No.3	11 (3/3)	Sp.Rot	62.0310	0.1008<br>0.0000	17.3<br>50.00<br>Cell	Tue Nov 02 14:14:30 2010<br>0.00325g/mlCHCl3<br>HLD10	Na<br>589nm	2 sec<br>10 sec

+62.1538°

Fig. 19S ^{1}H NMR spectra of 3

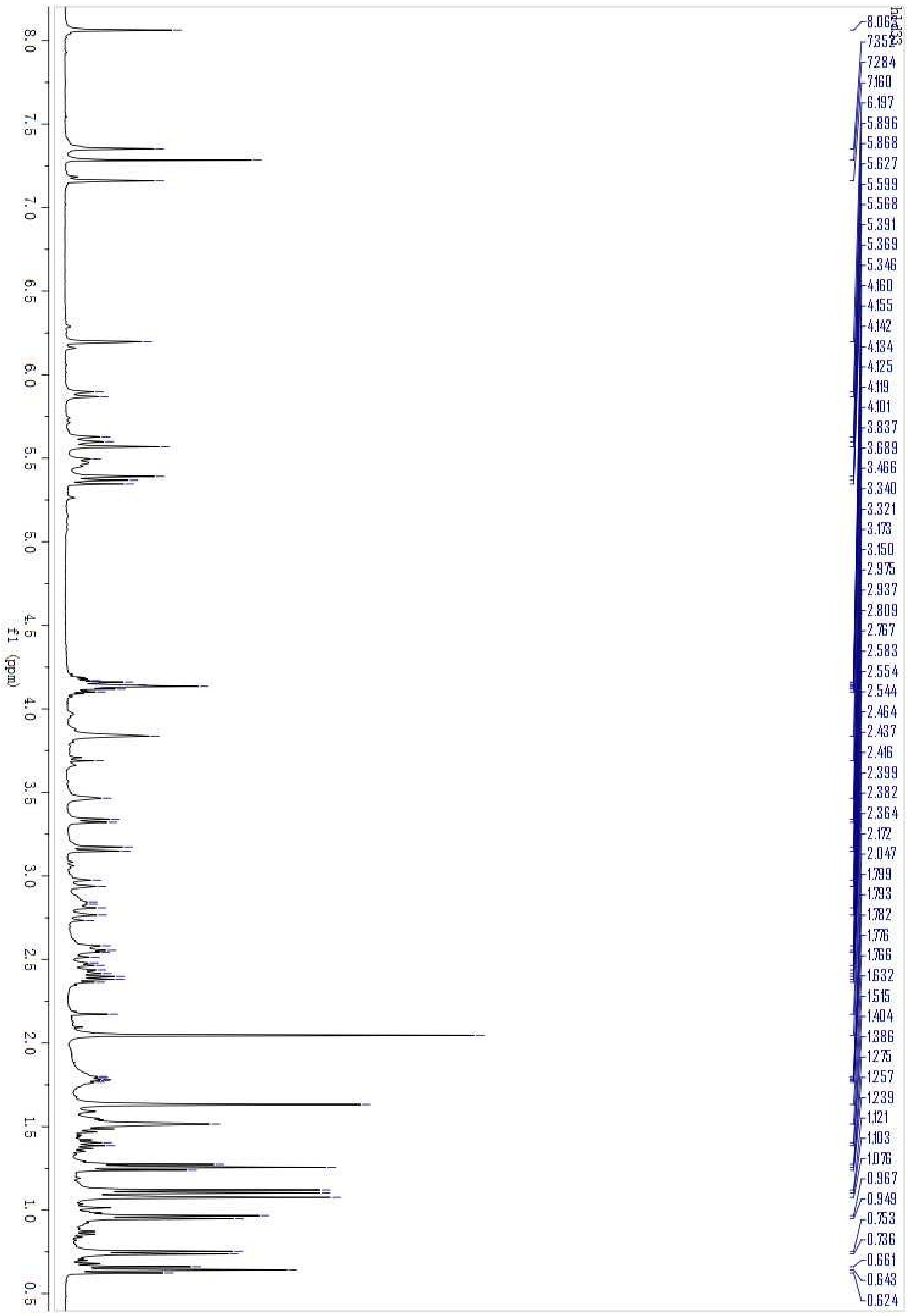

Fig. 20S ^{13}C NMR spectra of 3

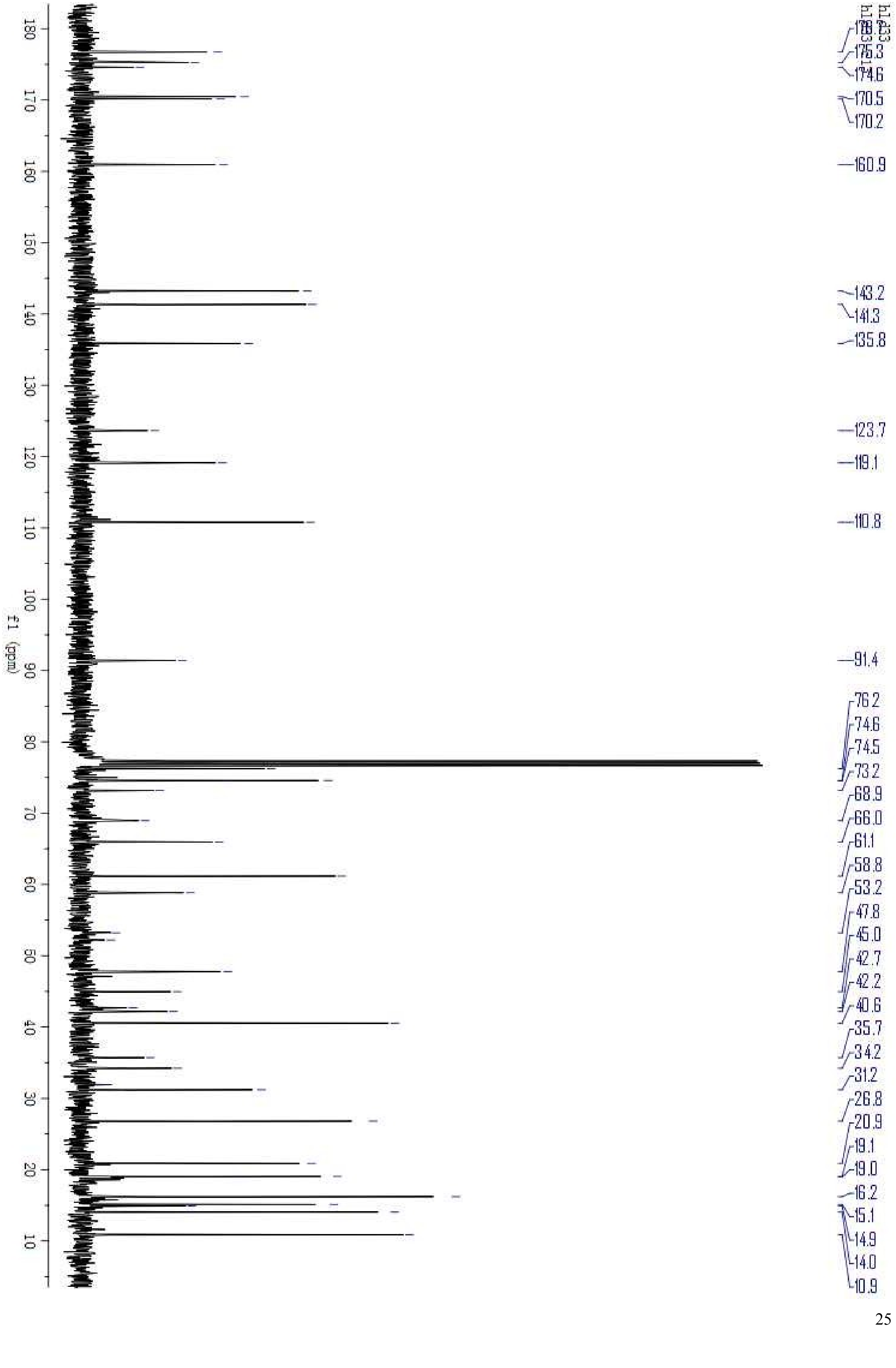

Fig. 21S HSQC spectra of 3

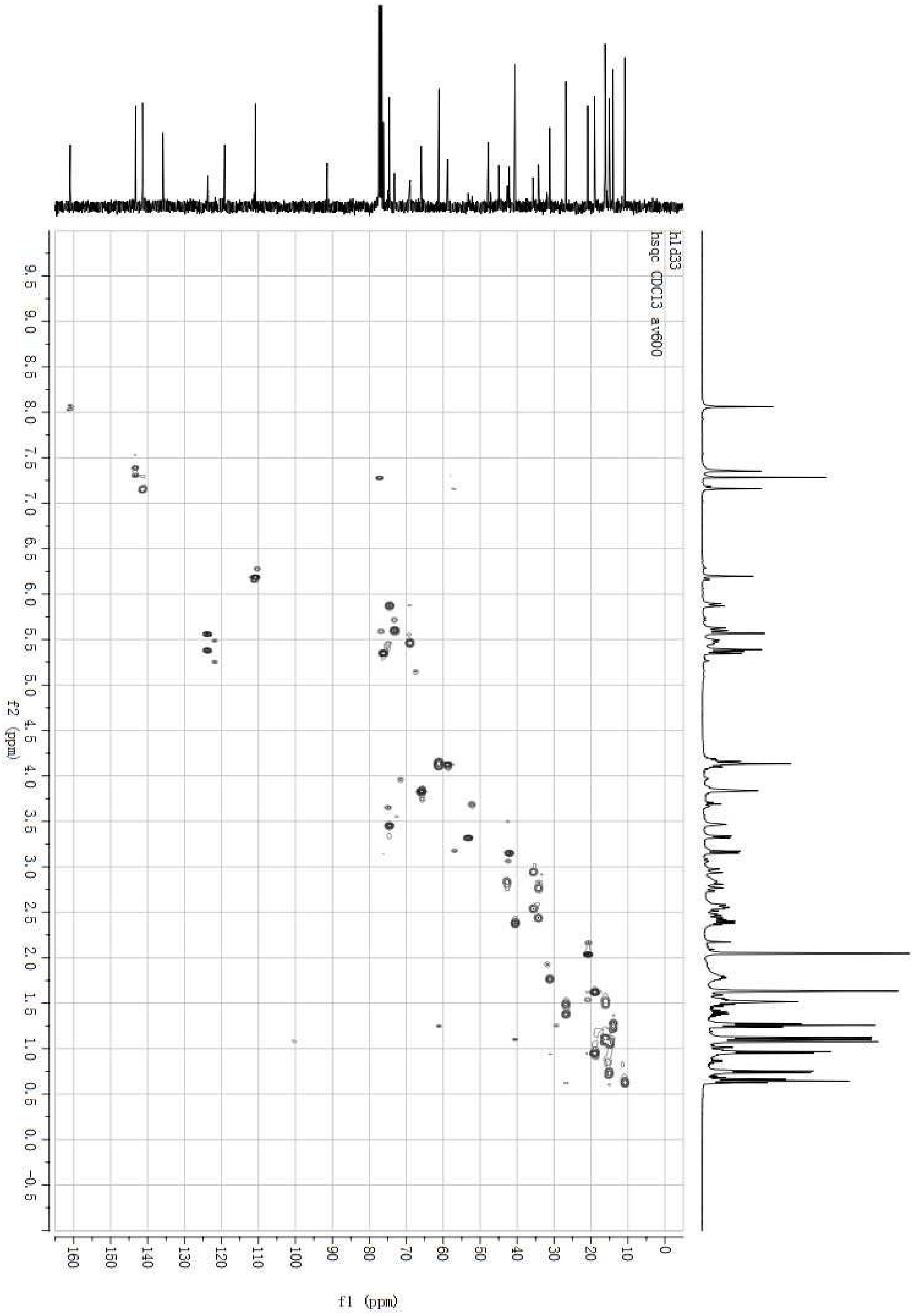

Fig. 22S HMBC spectra of 3

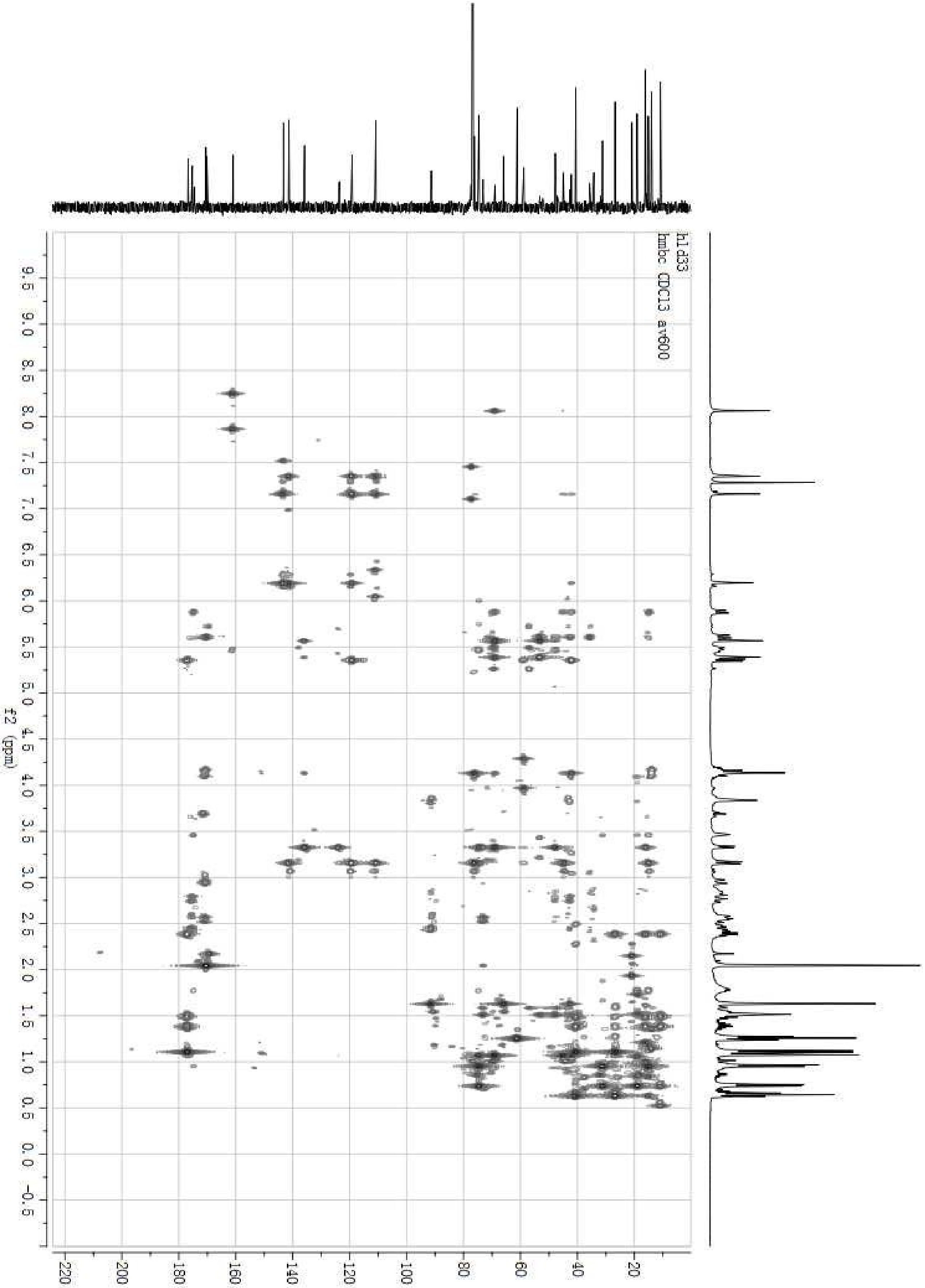

Fig. 23S COSY spectra of 3

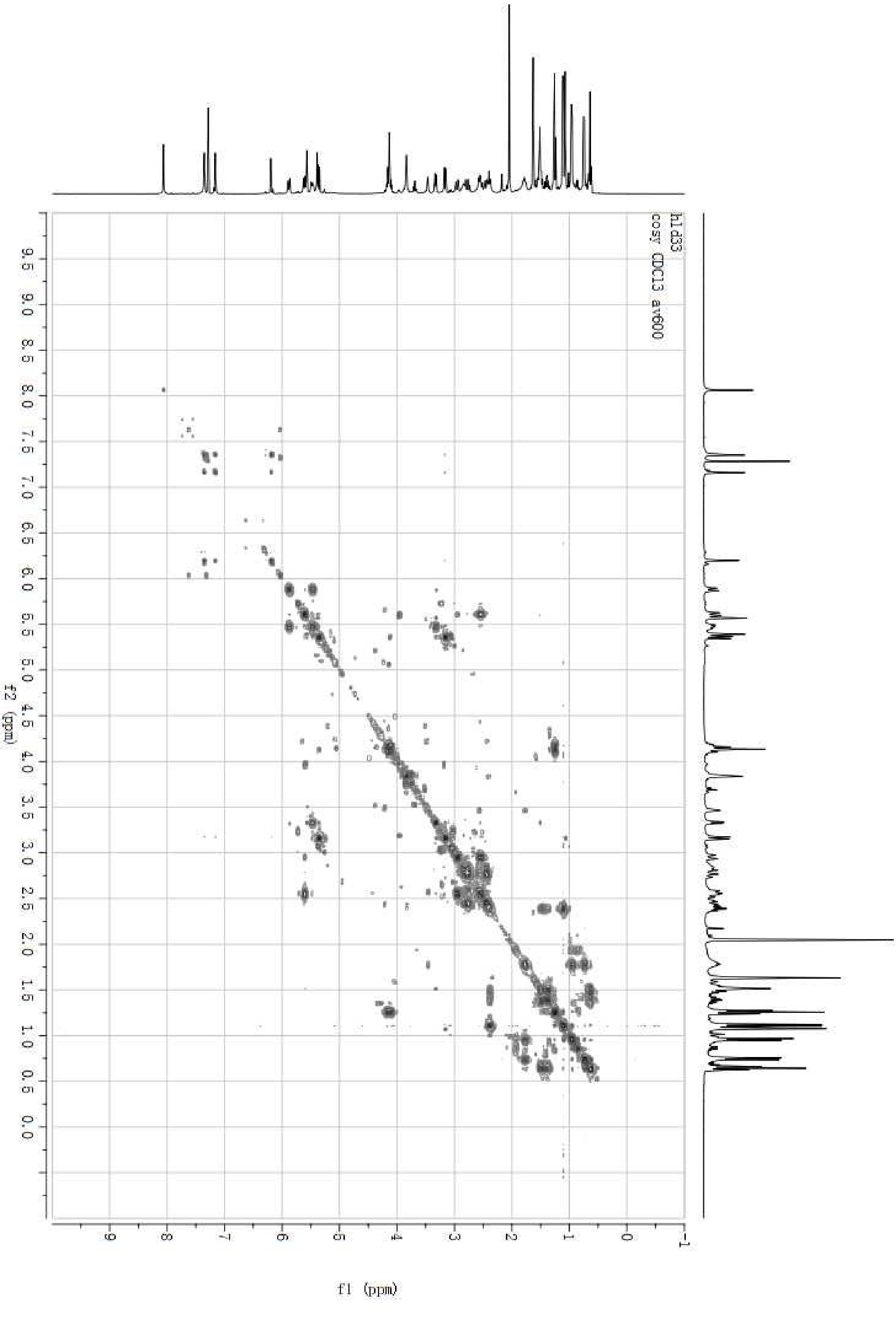

Fig. 24S HRMS spectra of 3

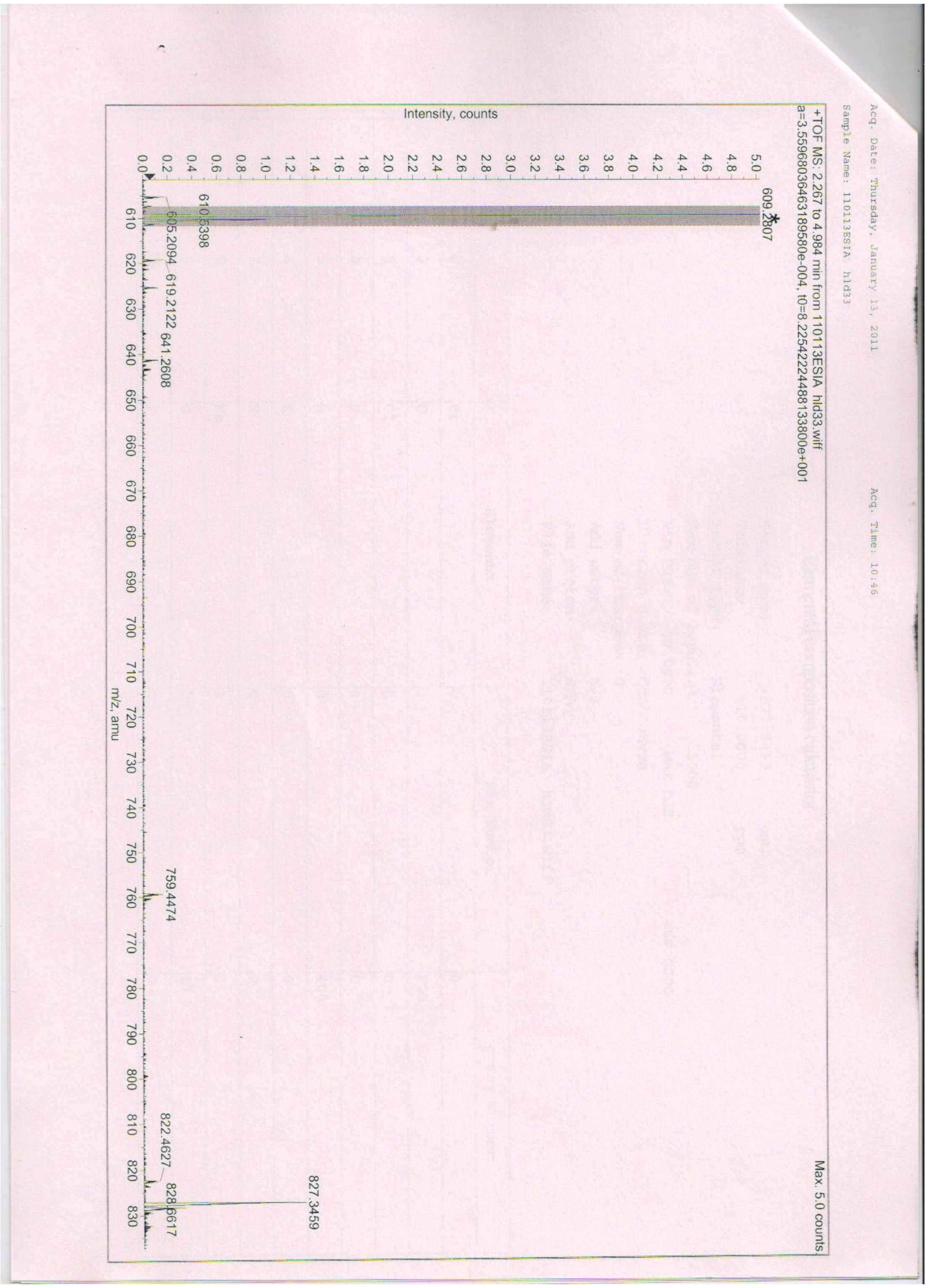

Fig. 25S IR spectra of 3

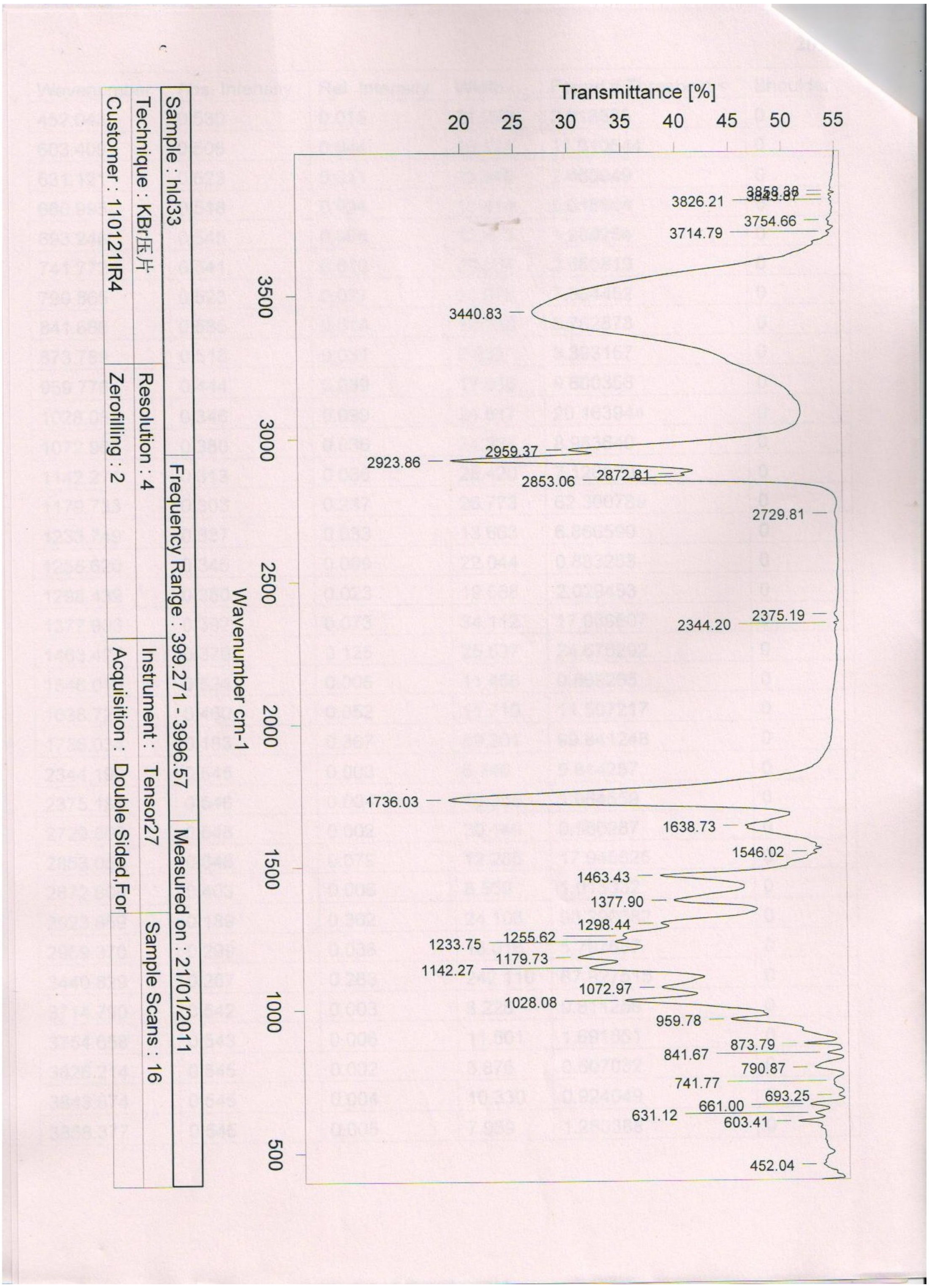

Fig. 26S Optical rotation spectra of 3

Optical rotation measurement

Model : P-1020 (A060460638)

No.	Sample	Mode	Data	Monitor<br>Blank	Temp.<br>Cell<br>Temp Point	Date<br>Comment<br>Sample Name	Light<br>Filter<br>Operator	Cycle Time<br>Integ Time
No.1	15 (1/3)	Sp.Rot	24.6250	0.0197<br>0.0000	11.4<br>50.00<br>Cell	Fri Jan 14 10:19:00 2011<br>0.00160g/mlCHCl3<br>HLD33	Na<br>589nm	2 sec<br>10 sec
No.2	15 (2/3)	Sp.Rot	24.5000	0.0196<br>0.0000	11.4<br>50.00<br>Cell	Fri Jan 14 10:19:13 2011<br>0.00160g/mlCHCl3<br>HLD33	Na<br>589nm	2 sec<br>10 sec
No.3	15 (3/3)	Sp.Rot	23.3750	0.0187<br>0.0000	11.4<br>50.00<br>Cell	Fri Jan 14 10:19:26 2011<br>0.00160g/mlCHCl3<br>HLD33	Na<br>589nm	2 sec<br>10 sec

+24.1667°

Fig. 27S ^{1}H NMR spectra of 4

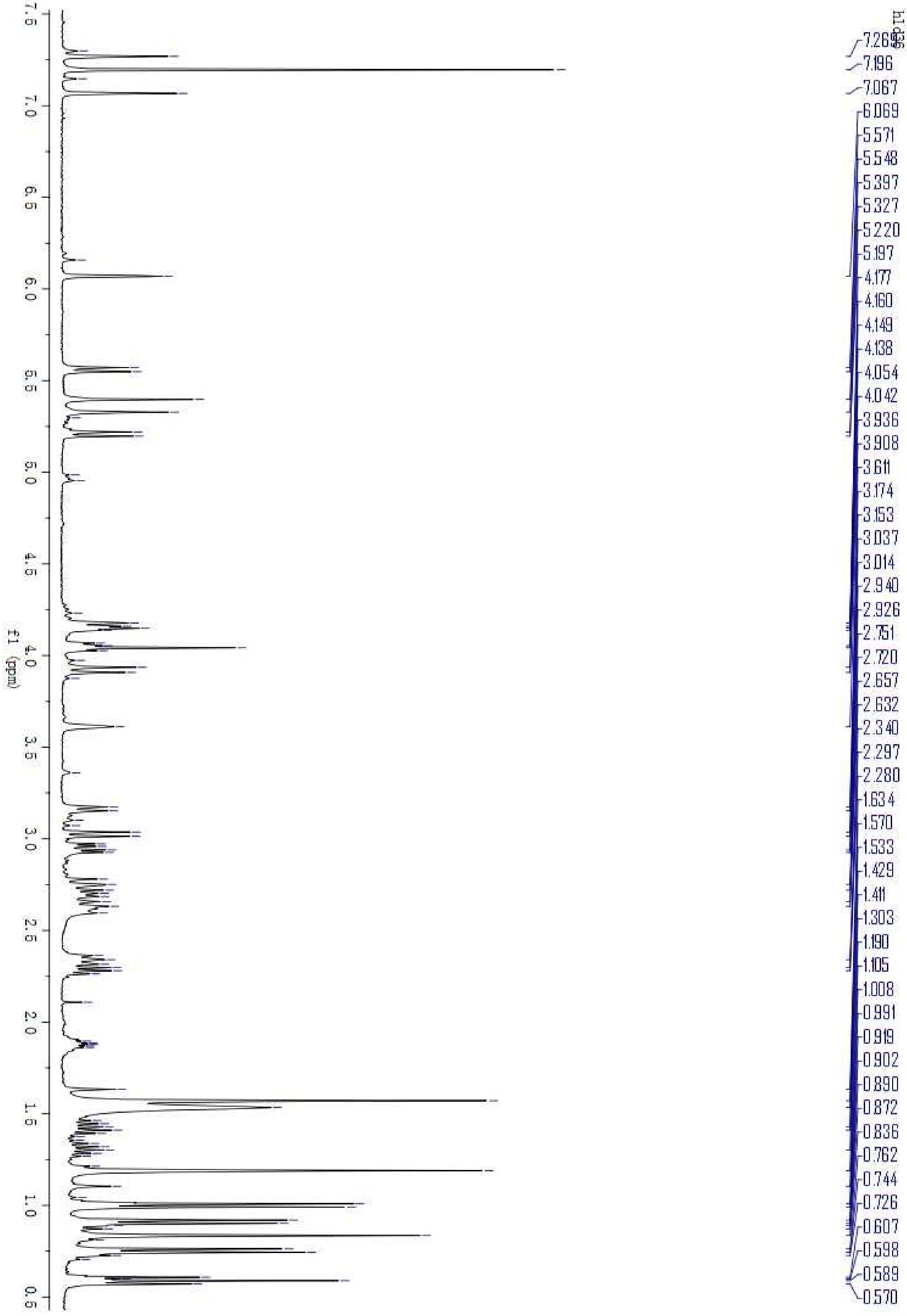

Fig. 28S ^{13}C NMR spectra of 4

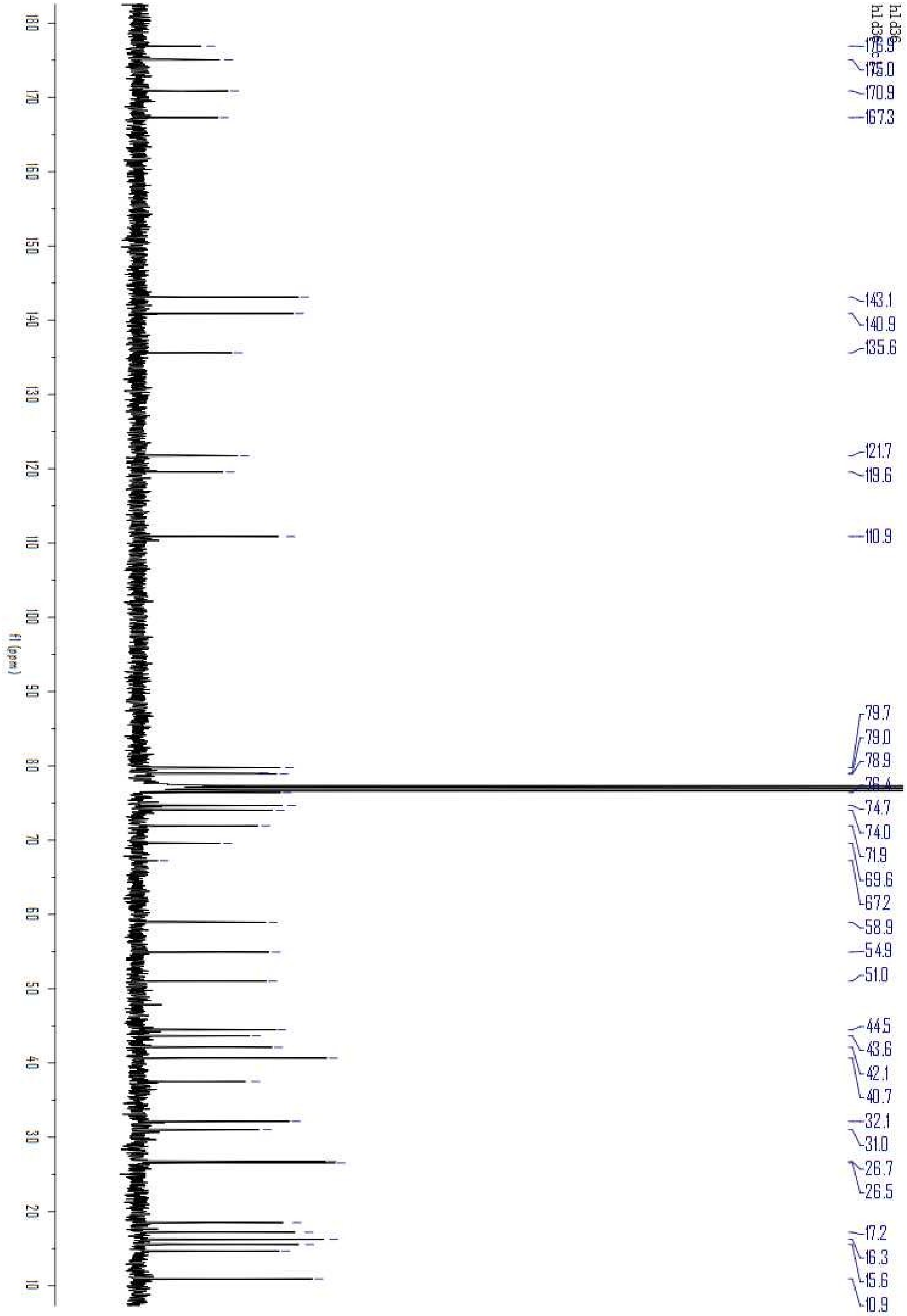

Fig. 29S HSQC spectra of 4

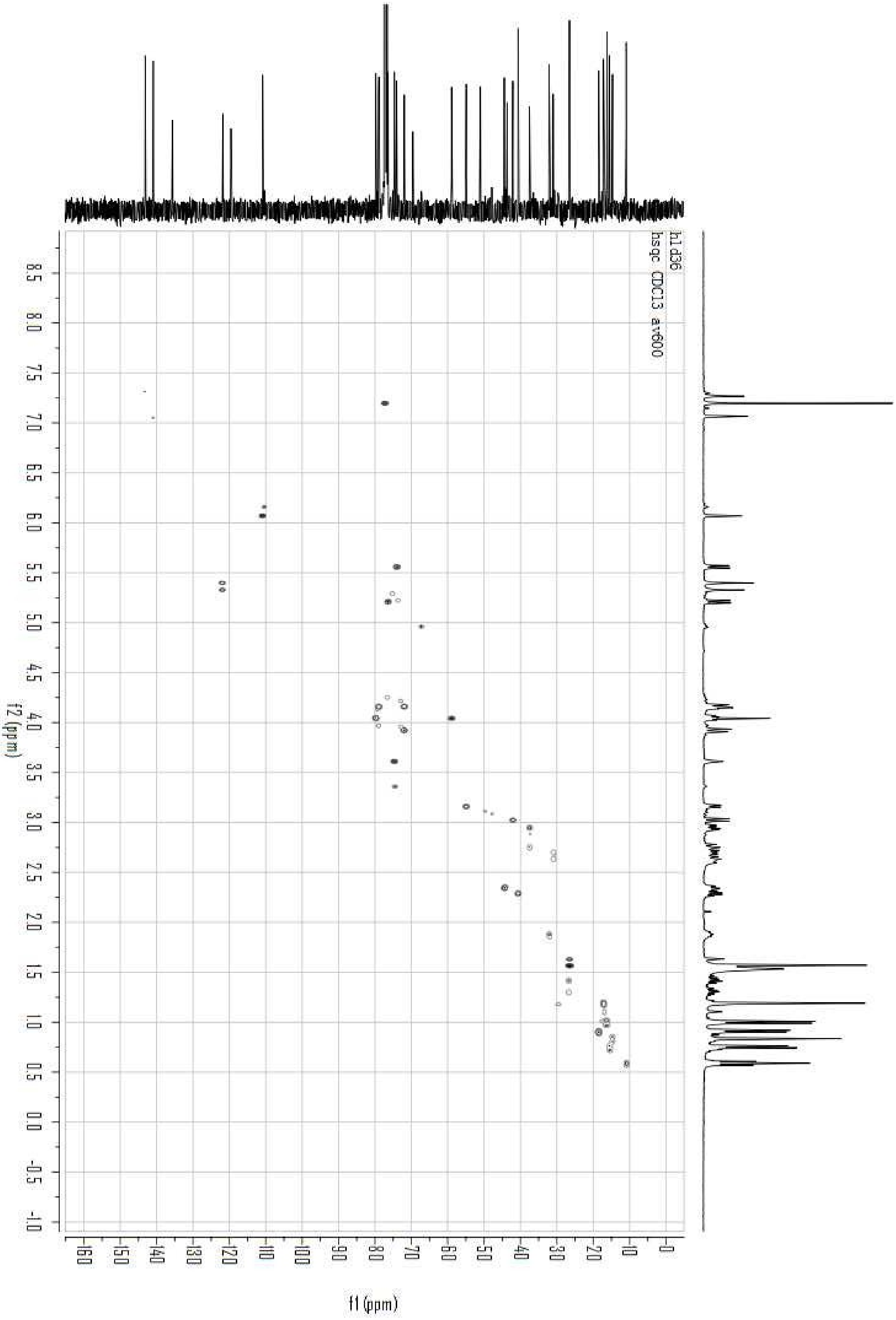

Fig. 30S HMBC spectra of 4

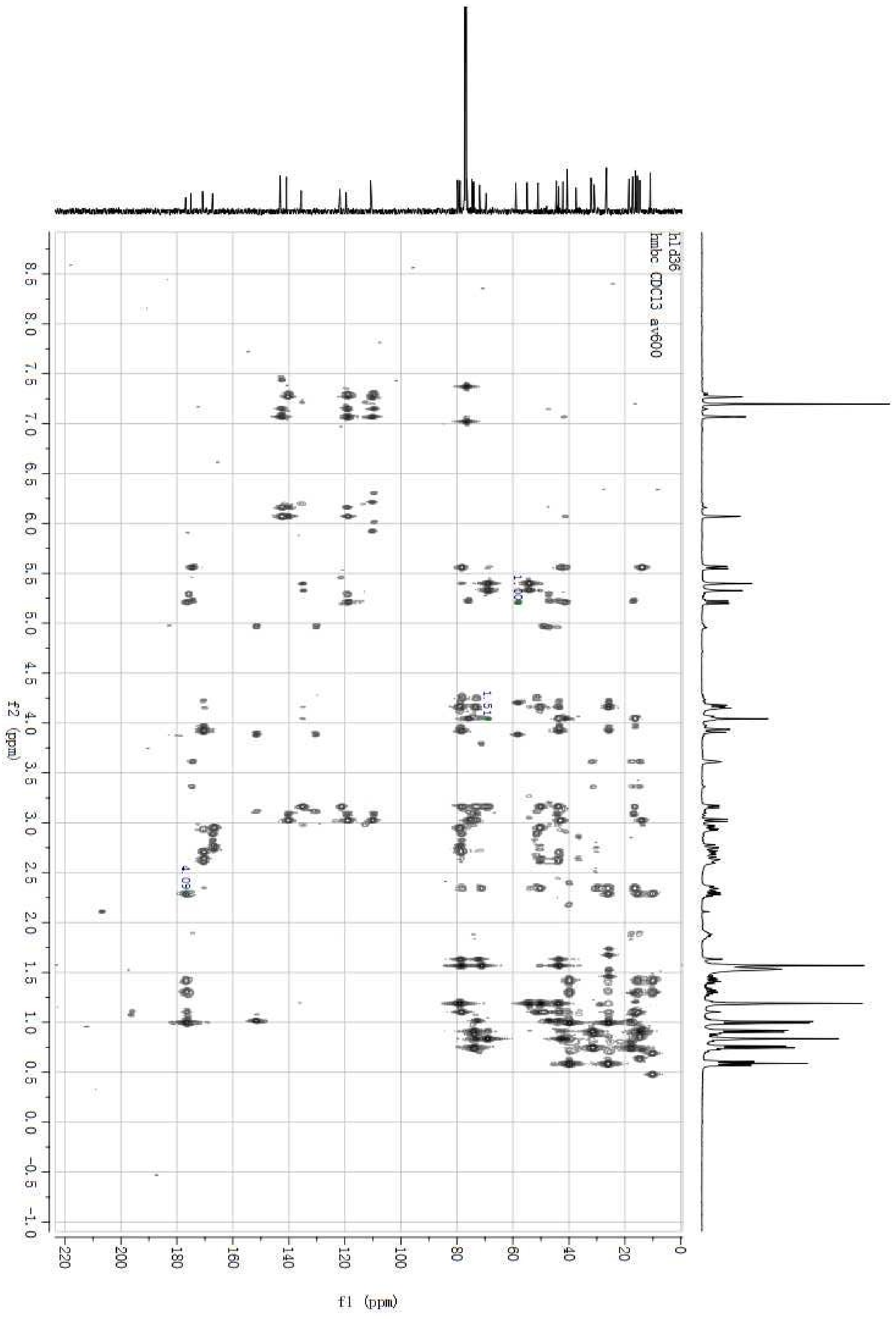

Fig. 31S COSY spectra of 4

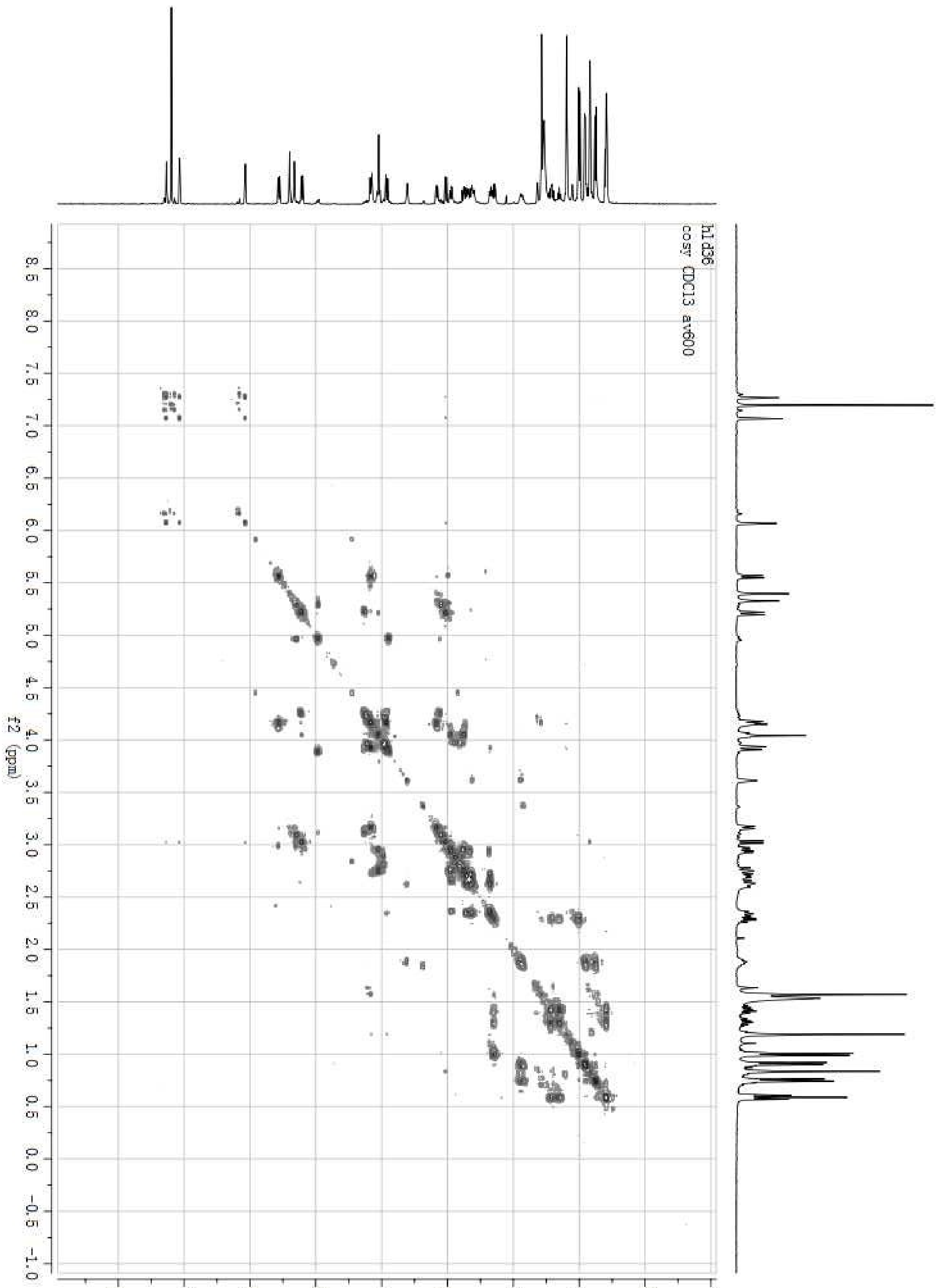

Fig. 32S COSY spectra of 4

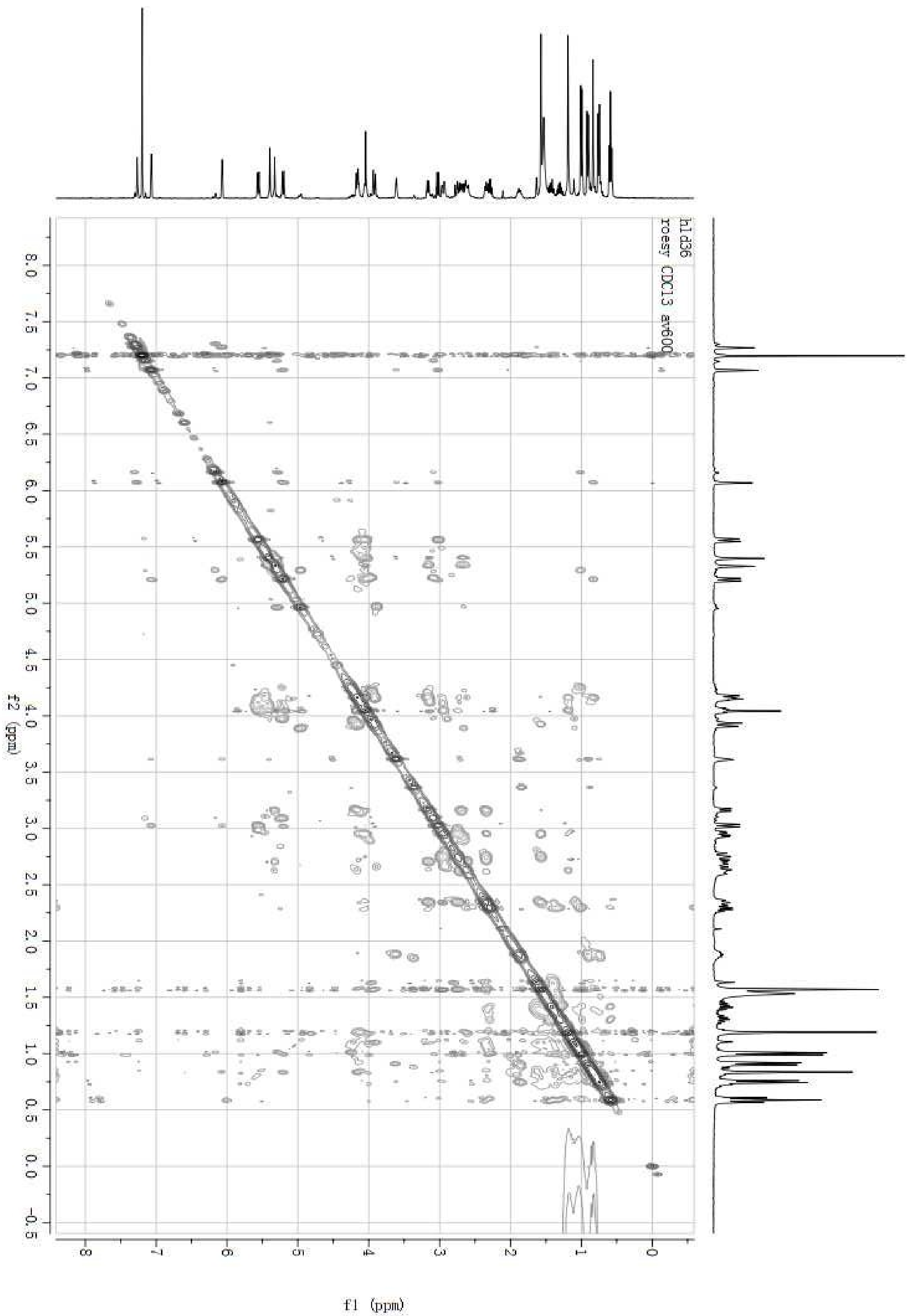

Fig. 33S HRMS spectra of 4

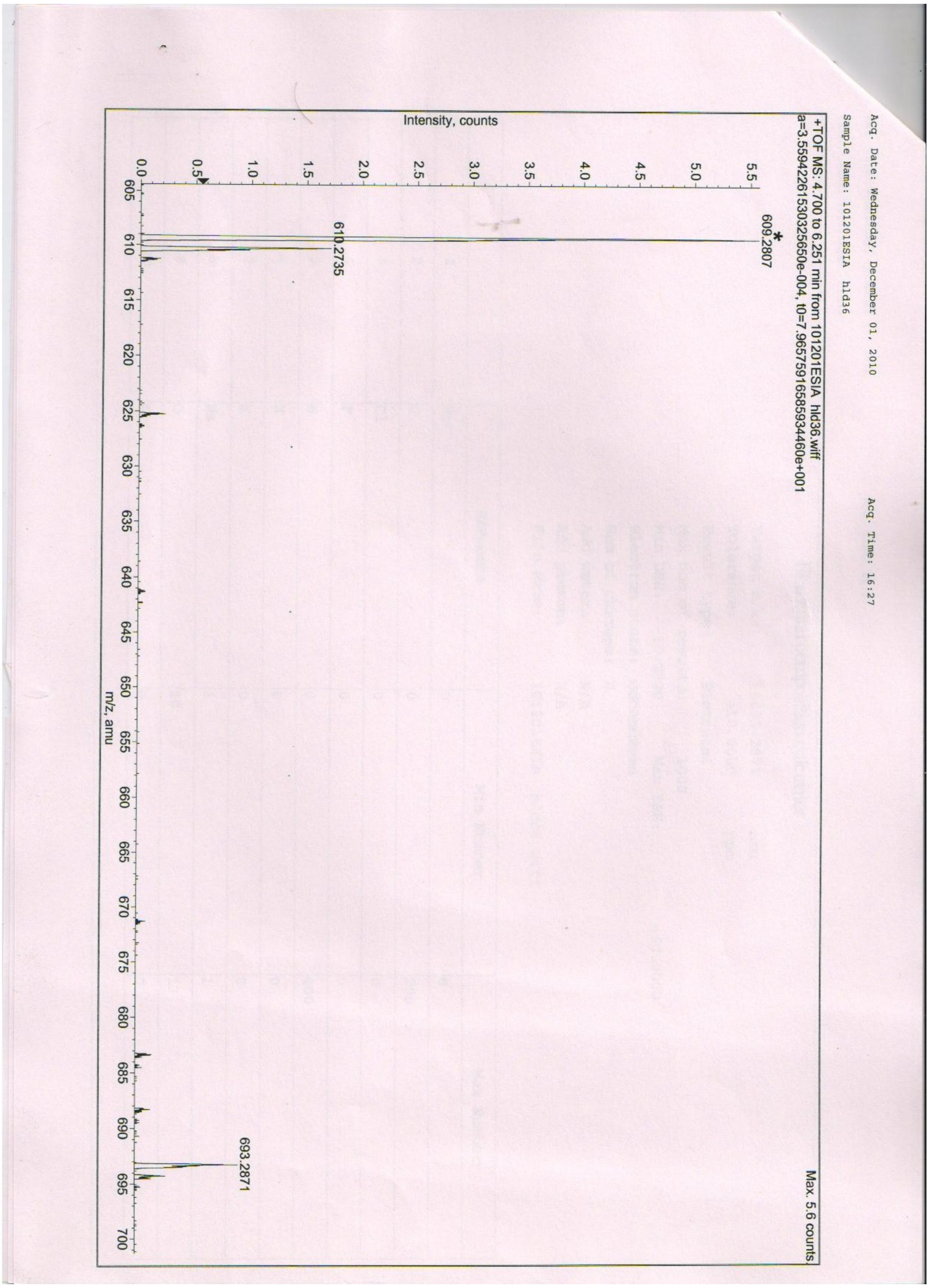

Fig. 34S IR spectra of 4

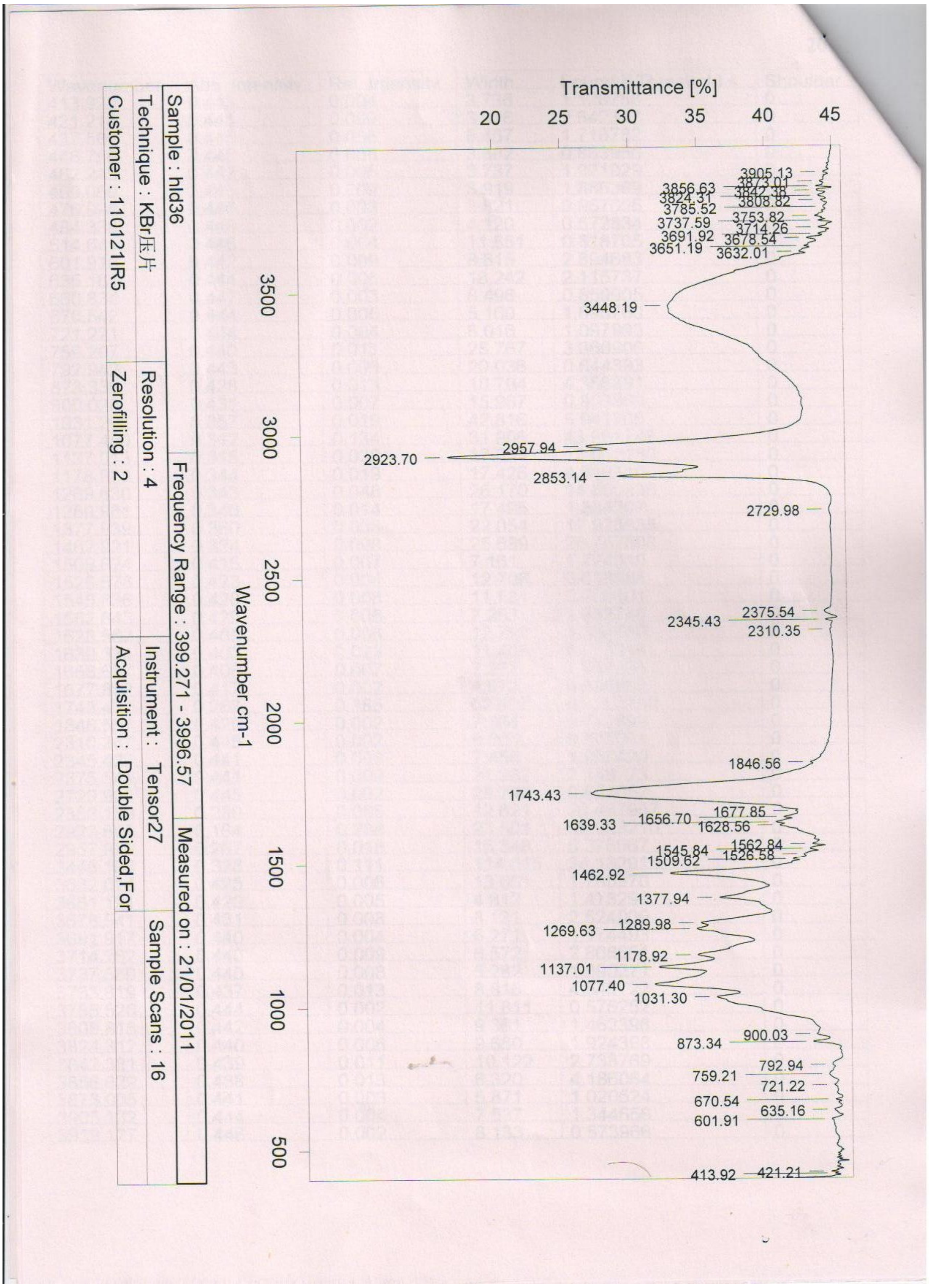

Fig. 35S Optical rotation spectra of 4

Optical rotation measurement

Model : P-1020 (A060460638)

No.	Sample	Mode	Data	Monitor<br>Blank	Temp.<br>Cell<br>Temp Point	Date<br>Comment<br>Sample Name	Light<br>Filter<br>Operator	Cycle Time<br>Integ Time
No.1	12 (1/3)	Sp.Rot	-24.2500	-0.0097<br>0.0000	11.1<br>50.00<br>Cell	Fri Jan 14 10:04:39 2011<br>0.00080g/mlCHCl3<br>HLD36	Na<br>589nm	2 sec<br>10 sec
No.2	12 (2/3)	Sp.Rot	-14.7500	-0.0059<br>0.0000	11.1<br>50.00<br>Cell	Fri Jan 14 10:04:52 2011<br>0.00080g/mlCHCl3<br>HLD36	Na<br>589nm	2 sec<br>10 sec
No.3	12 (3/3)	Sp.Rot	-17.0000	-0.0068<br>0.0000	11.1<br>50.00<br>Cell	Fri Jan 14 10:05:06 2011<br>0.00080g/mlCHCl3<br>HLD36	Na<br>589nm	2 sec<br>10 sec
No.4	13 (1/3)	Sp.Rot	-17.5000	-0.0070<br>0.0000	11.2<br>50.00<br>Cell	Fri Jan 14 10:06:12 2011<br>0.00080g/mlCHCl3<br>HLD36	Na<br>589nm	2 sec<br>10 sec
No.5	13 (2/3)	Sp.Rot	-23.7500	-0.0095<br>0.0000	11.1<br>50.00<br>Cell	Fri Jan 14 10:06:26 2011<br>0.00080g/mlCHCl3<br>HLD36	Na<br>589nm	2 sec<br>10 sec
No.6	13 (3/3)	Sp.Rot	-16.2500	-0.0065<br>0.0000	11.2<br>50.00<br>Cell	Fri Jan 14 10:06:39 2011<br>0.00080g/mlCHCl3<br>HLD36	Na<br>589nm	2 sec<br>10 sec
No.7	14 (1/3)	Sp.Rot	-14.7500	-0.0059<br>0.0000	11.2<br>50.00<br>Cell	Fri Jan 14 10:07:14 2011<br>0.00080g/mlCHCl3<br>HLD36	Na<br>589nm	2 sec<br>10 sec
No.8	14 (2/3)	Sp.Rot	-20.5000	-0.0082<br>0.0000	11.2<br>50.00<br>Cell	Fri Jan 14 10:07:27 2011<br>0.00080g/mlCHCl3<br>HLD36	Na<br>589nm	2 sec<br>10 sec
No.9	14 (3/3)	Sp.Rot	-20.7500	-0.0083<br>0.0000	11.2<br>50.00<br>Cell	Fri Jan 14 10:07:40 2011<br>0.00080g/mlCHCl3<br>HLD36	Na<br>589nm	2 sec<br>10 sec

-18.8334°

Fig. 36S ^{1}H NMR spectra of 5

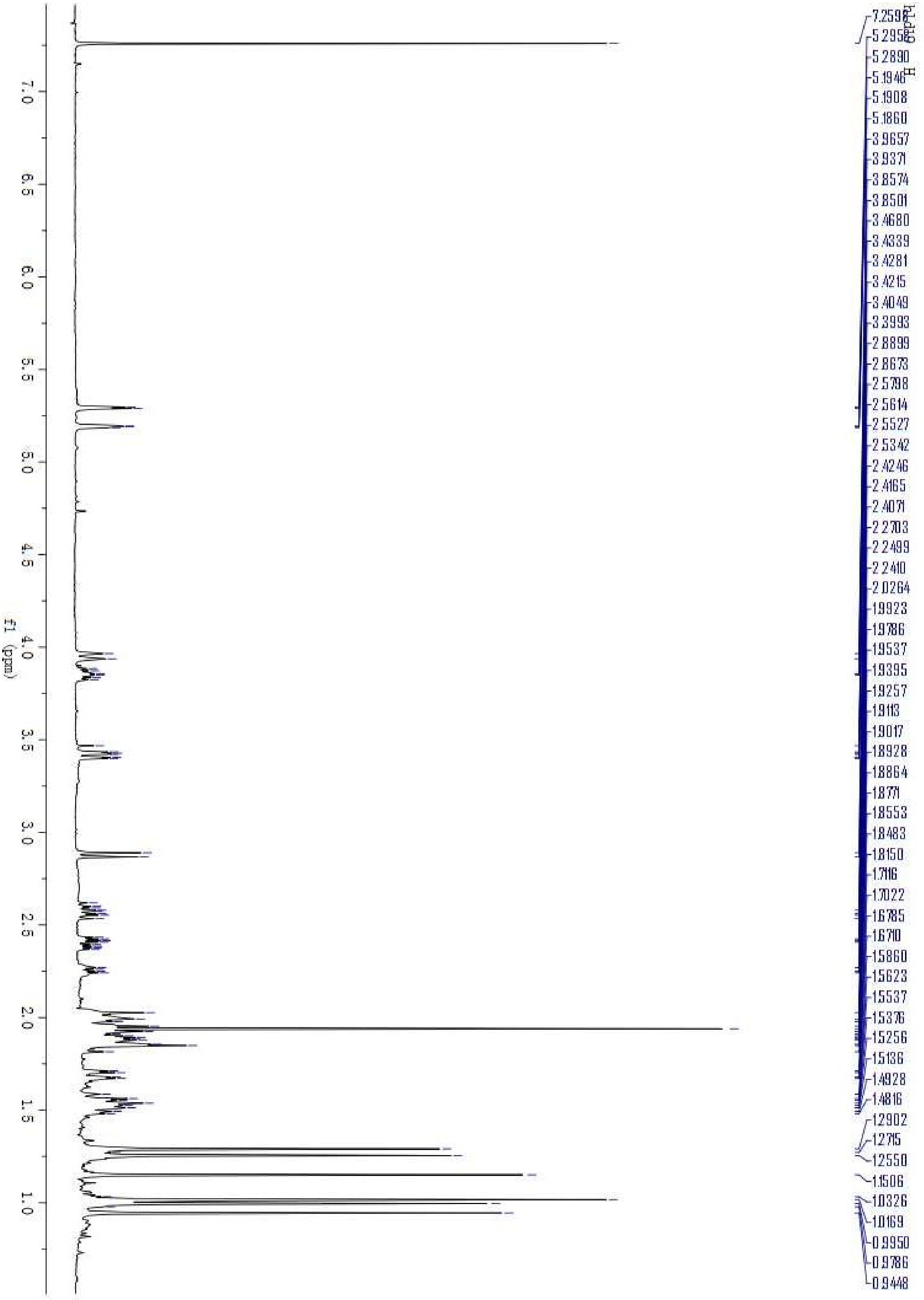

Fig. 37S ^{13}C NMR spectra of 5

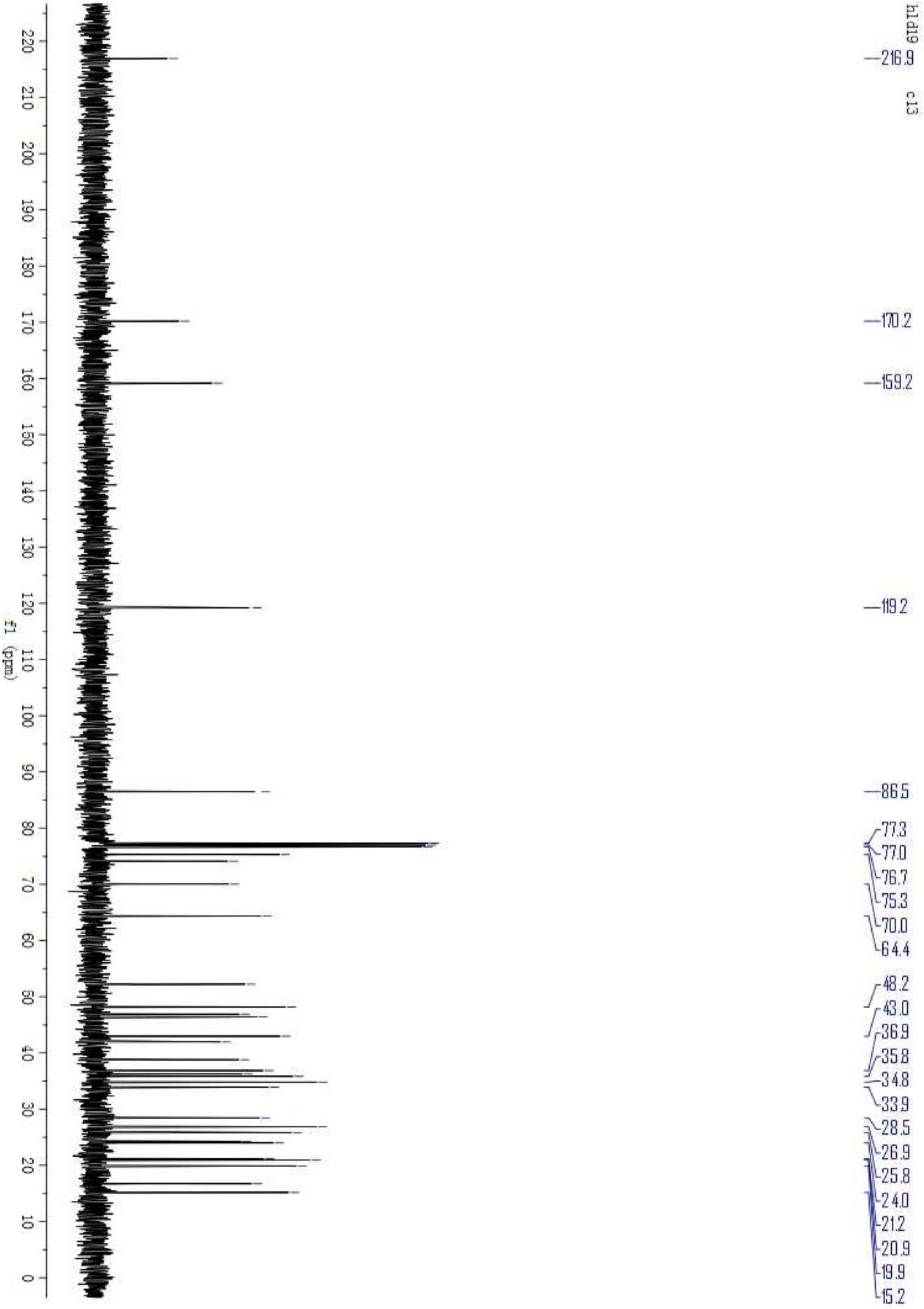

Fig. 38S HSQC spectra of 5

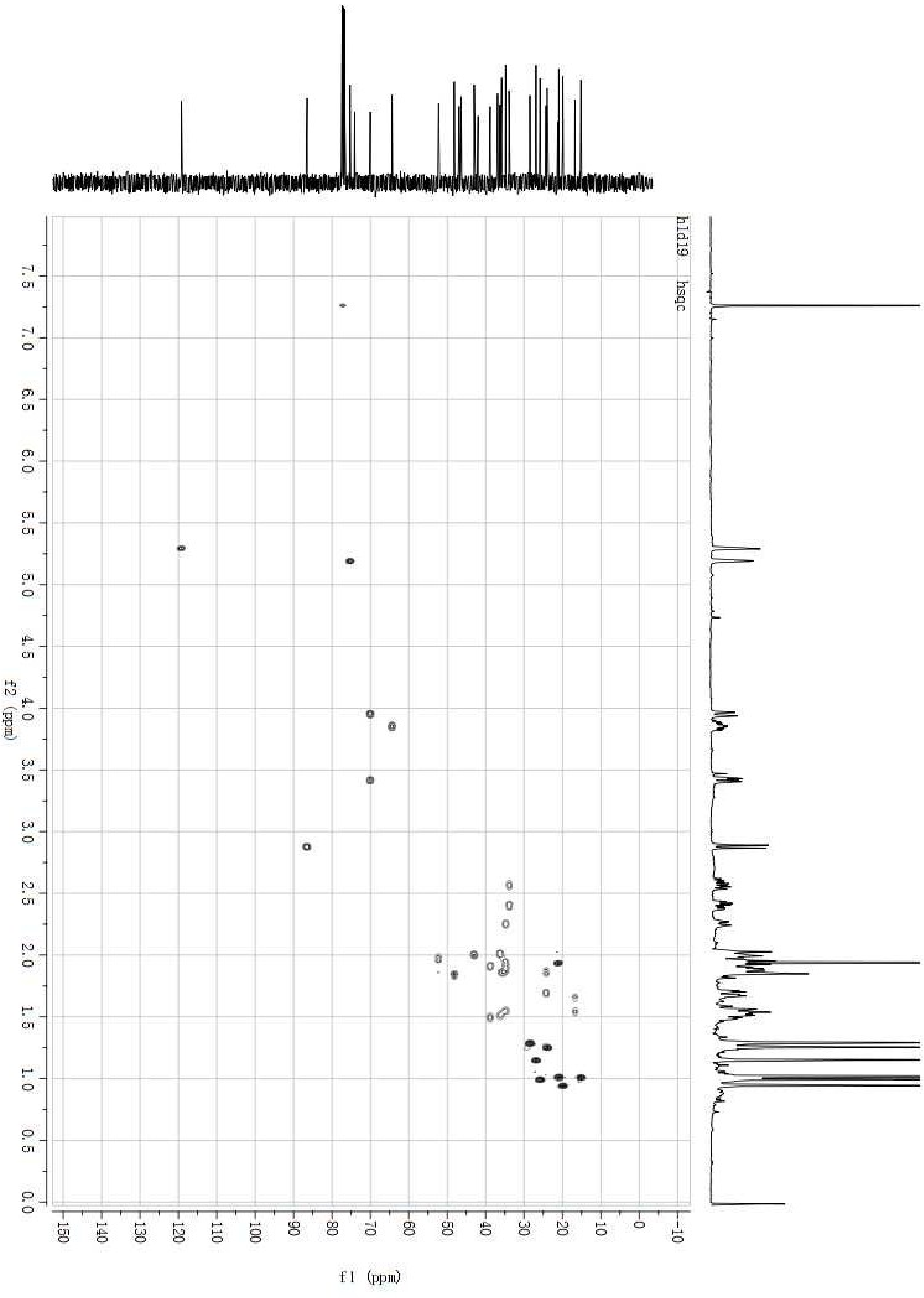

Fig. 39S HMBC spectra of 5

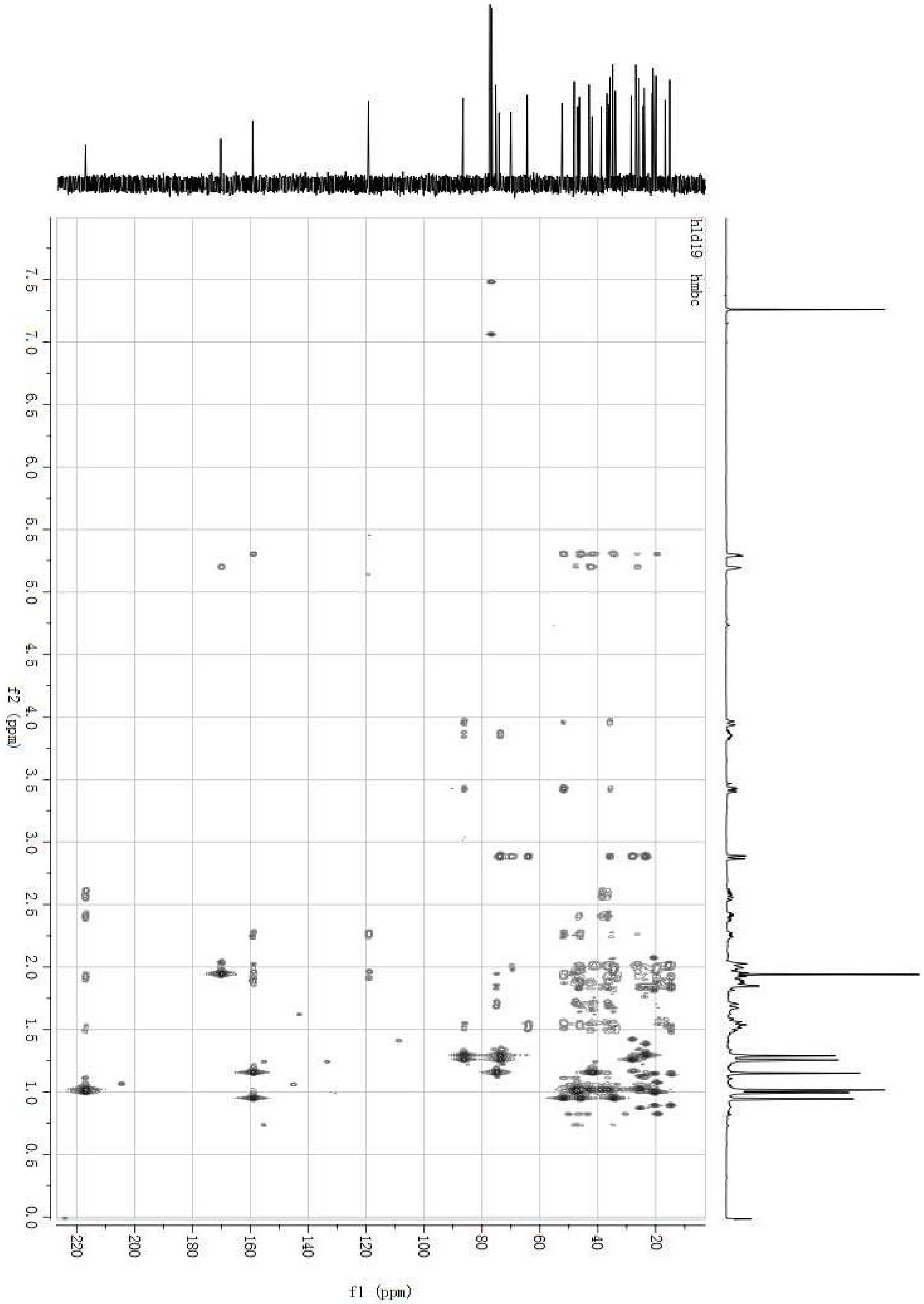

Fig. 40S COSY spectra of 5

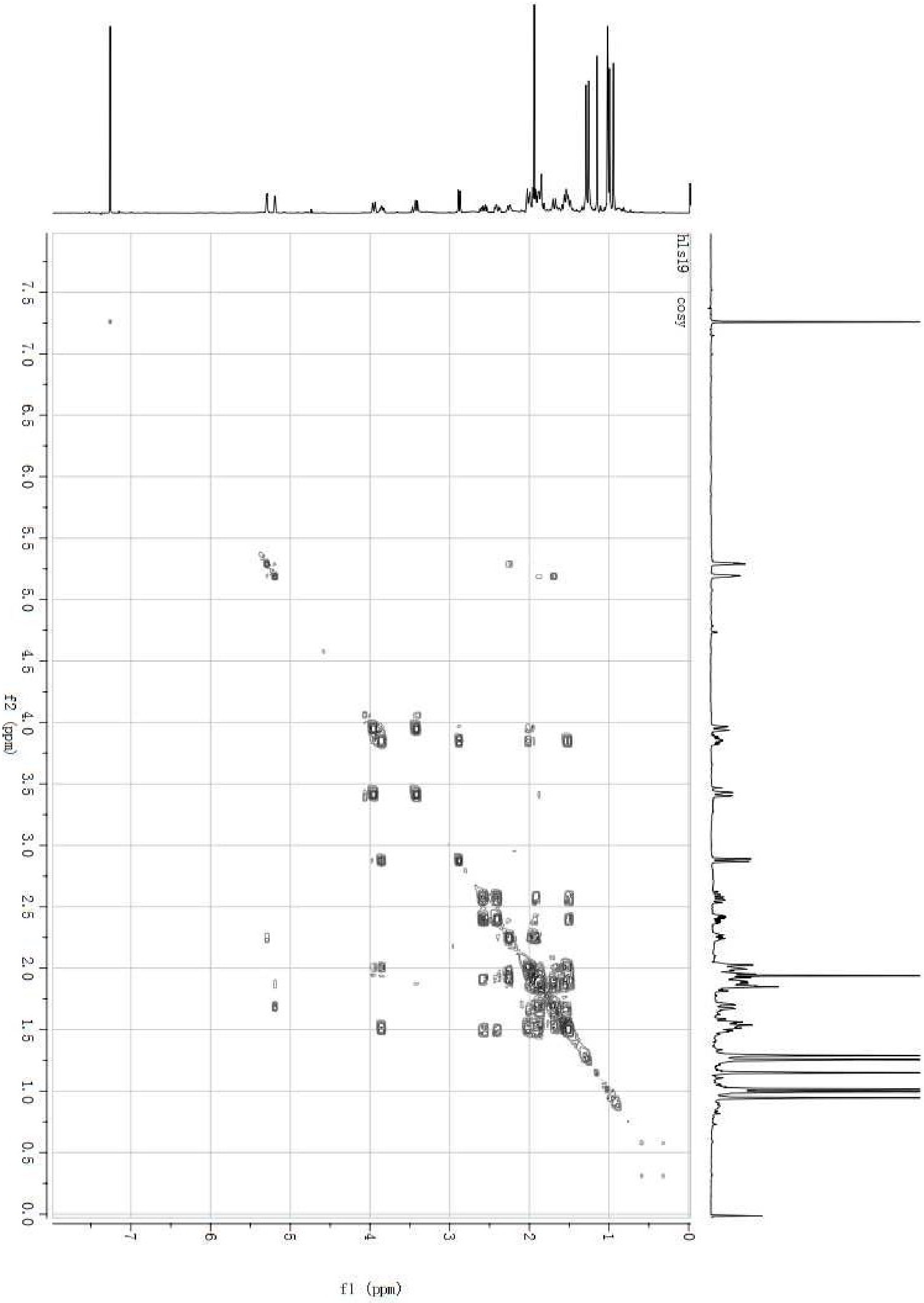

Fig. 41S HRMS spectra of 5

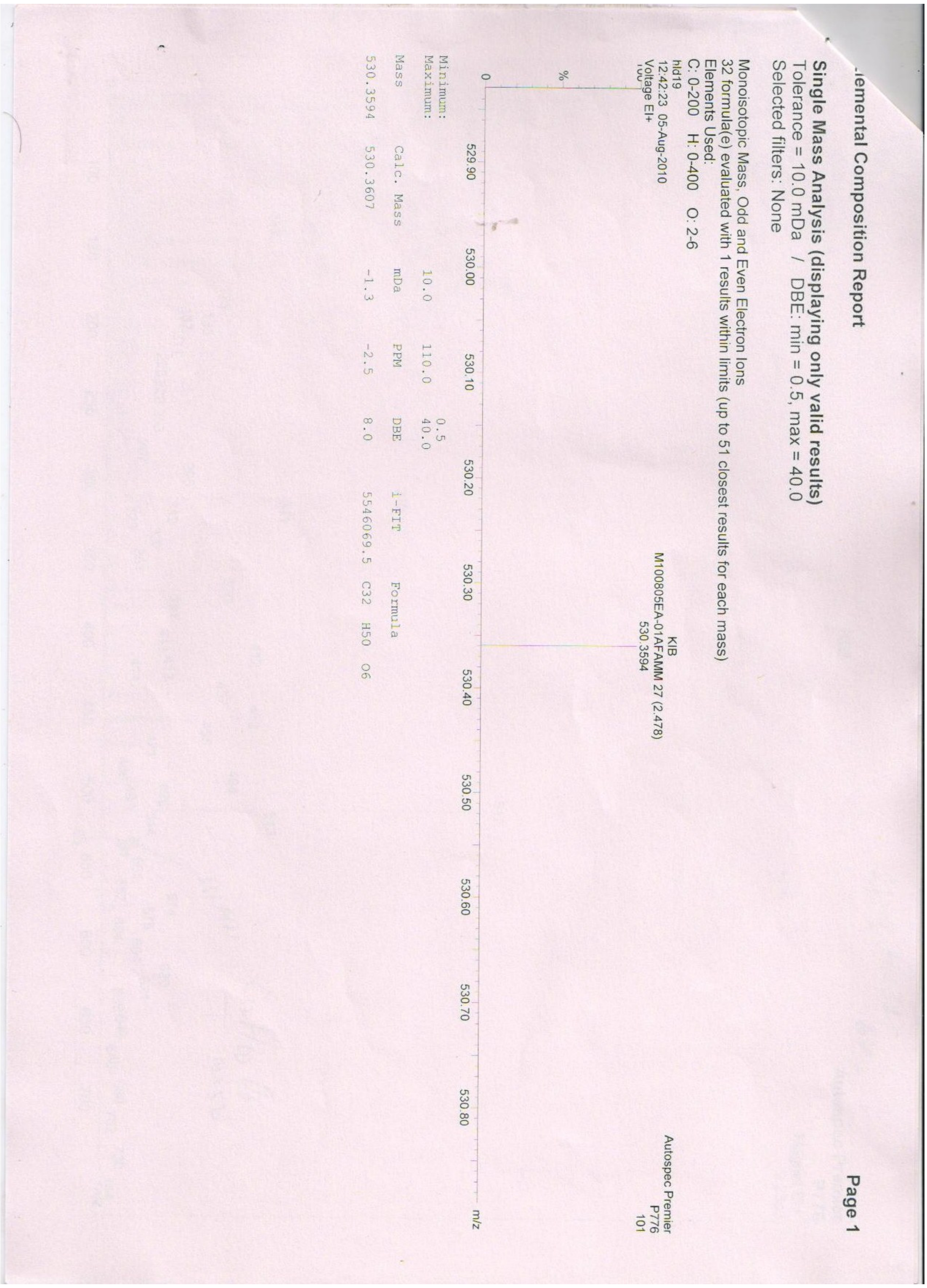

Fig. 42S IR spectra of 5

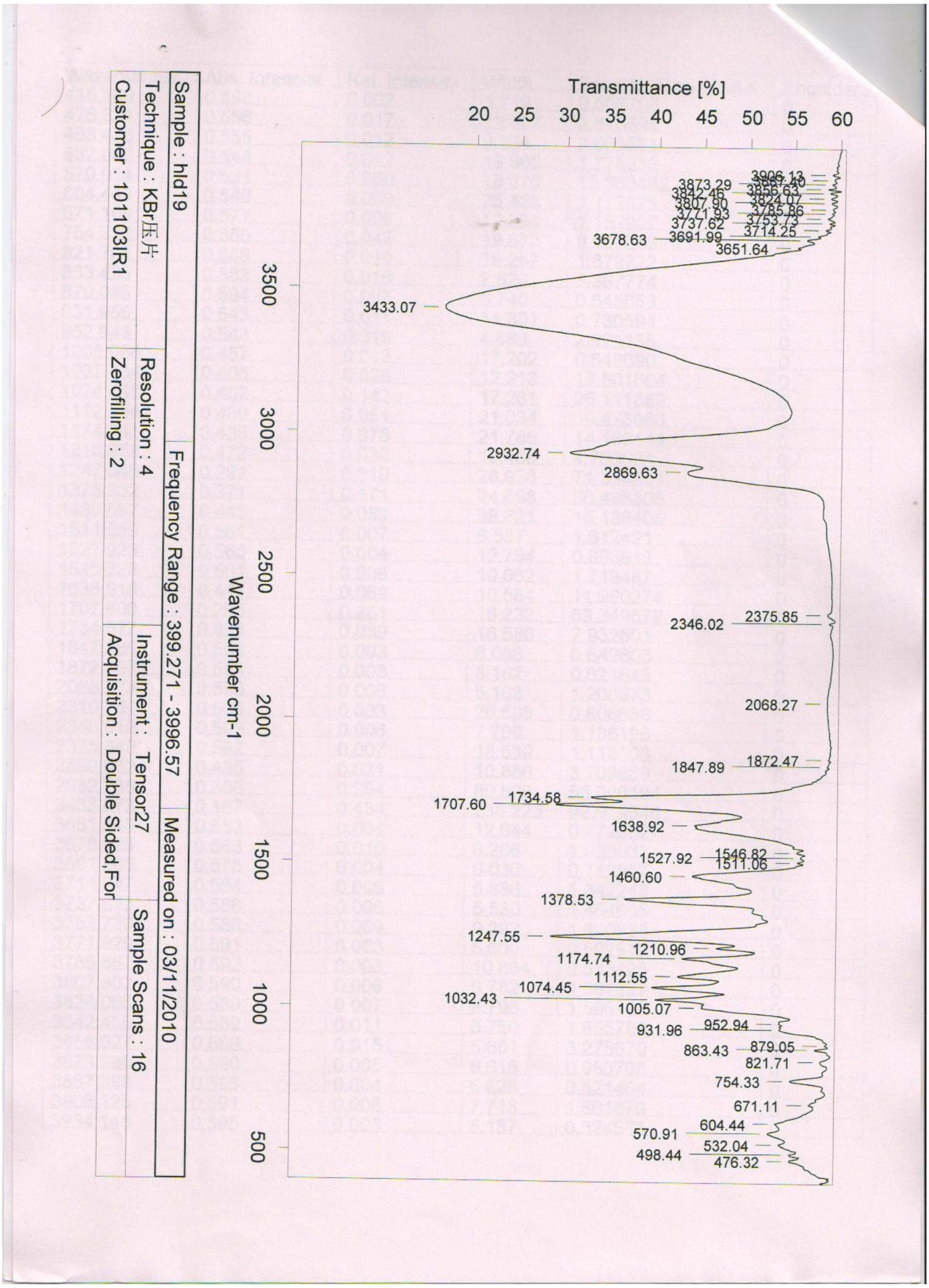

Fig. 43S Optical rotation spectra of 5

Optical rotation measurement

Model : P-1020 (A060460638)

No.	Sample	Mode	Data	Monitor<br>Blank	Temp.<br>Cell<br>Temp Point	Date<br>Comment<br>Sample Name	Light<br>Filter<br>Operator	Cycle Time<br>Integ Time
No.1	9 (1/3)	Sp.Rot	-35.4620	-0.0461<br>0.0000	17.1<br>50.00<br>Cell	Tue Nov 02 13:57:14 2010<br>0.00260g/mlCHCl3<br>HLD19	Na<br>589nm	2 sec<br>10 sec
No.2	9 (2/3)	Sp.Rot	-34.7690	-0.0452<br>0.0000	17.1<br>50.00<br>Cell	Tue Nov 02 13:57:27 2010<br>0.00260g/mlCHCl3<br>HLD19	Na<br>589nm	2 sec<br>10 sec
No.3	9 (3/3)	Sp.Rot	-33.9230	-0.0441<br>0.0000	17.1<br>50.00<br>Cell	Tue Nov 02 13:57:41 2010<br>0.00260g/mlCHCl3<br>HLD19	Na<br>589nm	2 sec<br>10 sec

-34.7179°

Fig. 44S ^{1}H NMR spectra of 6

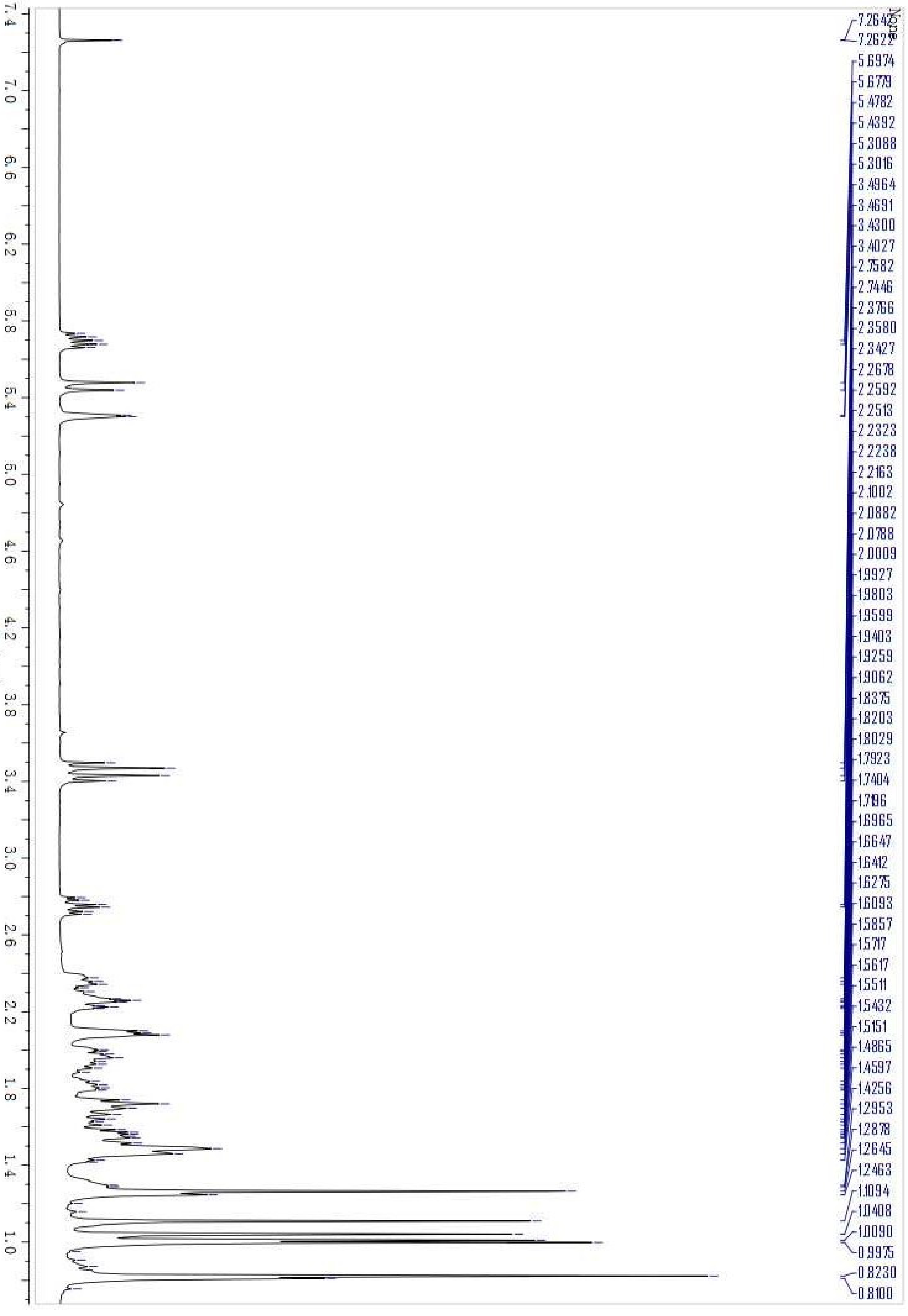

Fig. 45S ^{13}C NMR spectra of 6

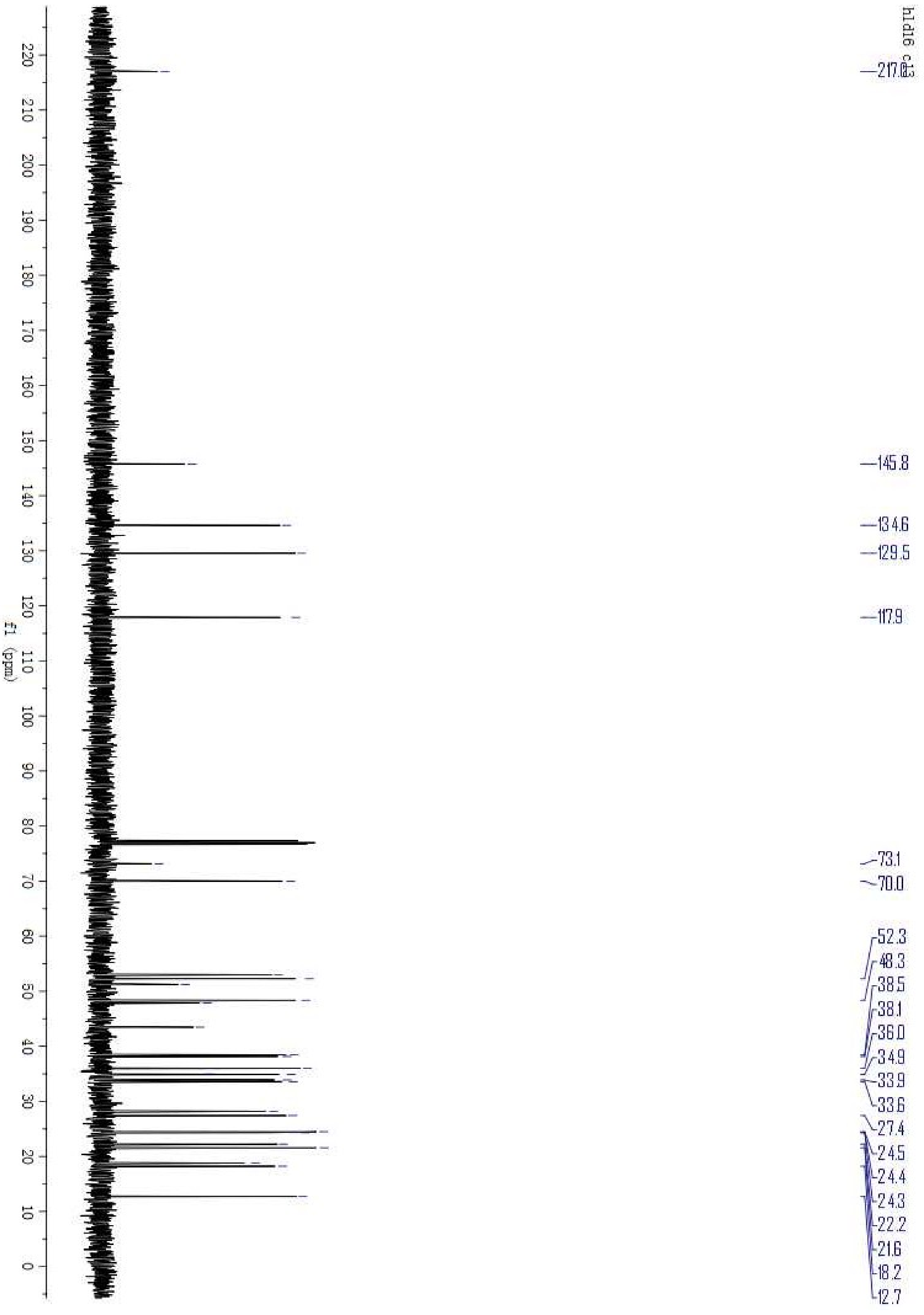

Fig. 46S HSQC spectra of 6

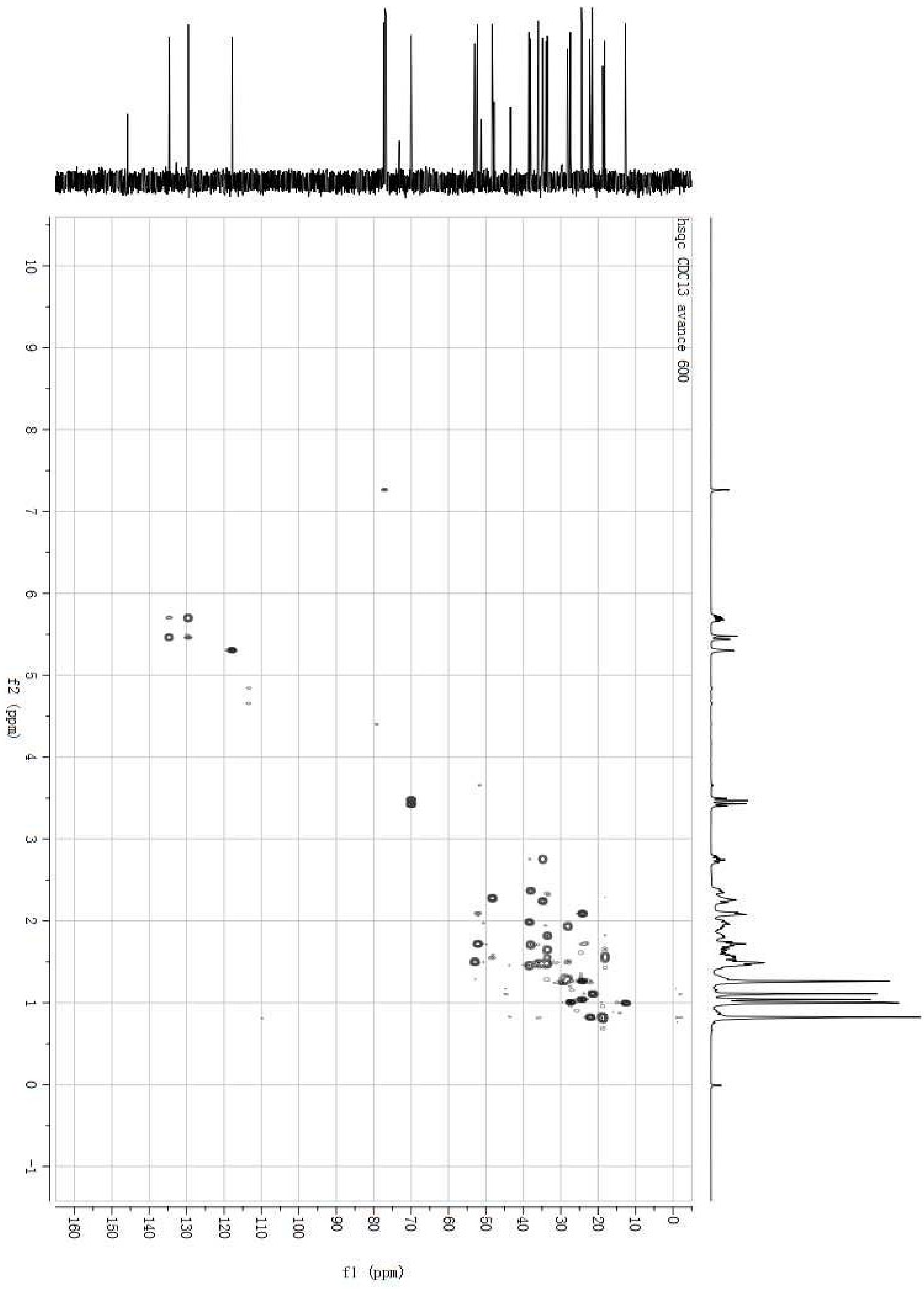

Fig. 47S HMBC spectra of 6

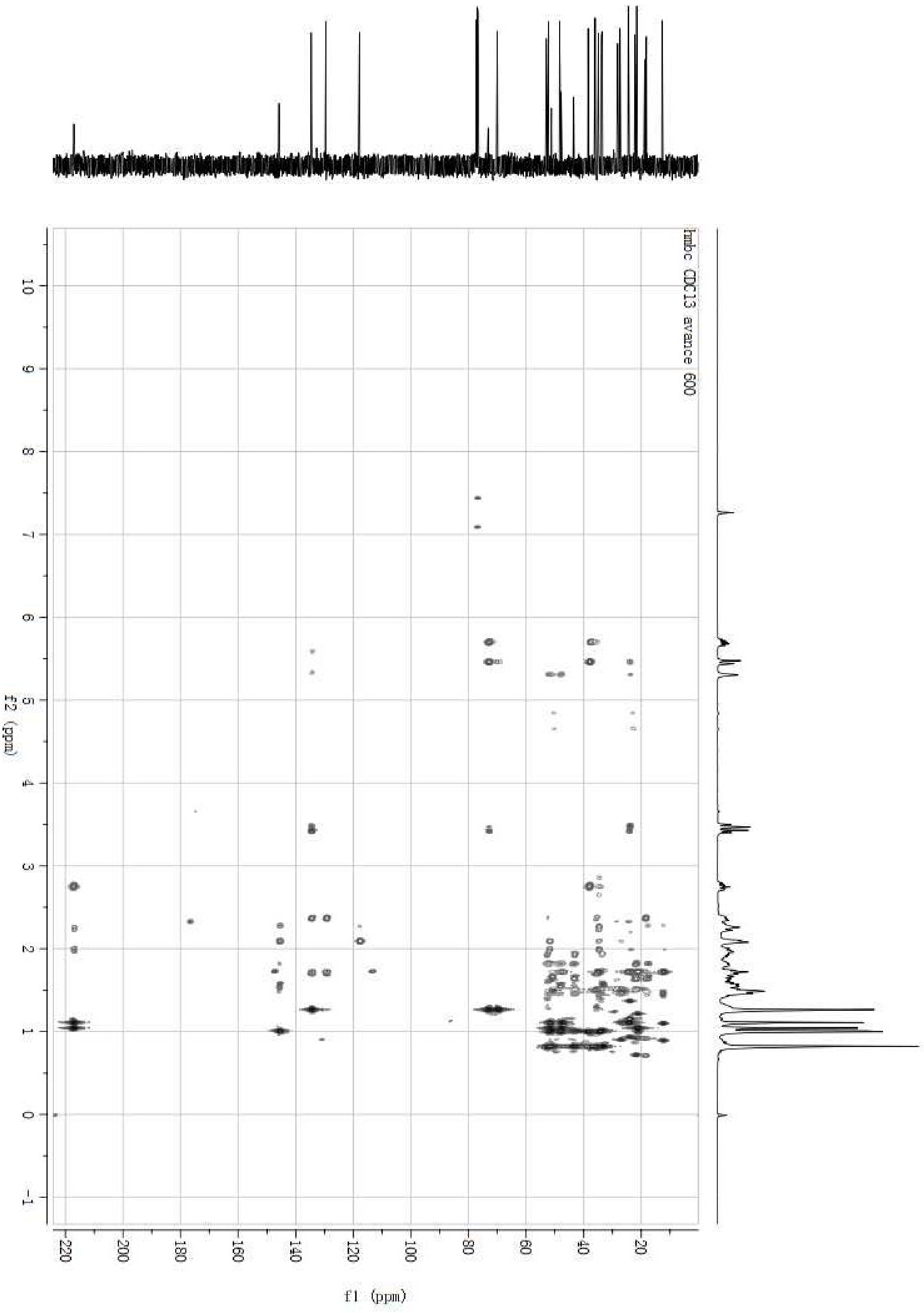

Fig. 48S COSY spectra of 6

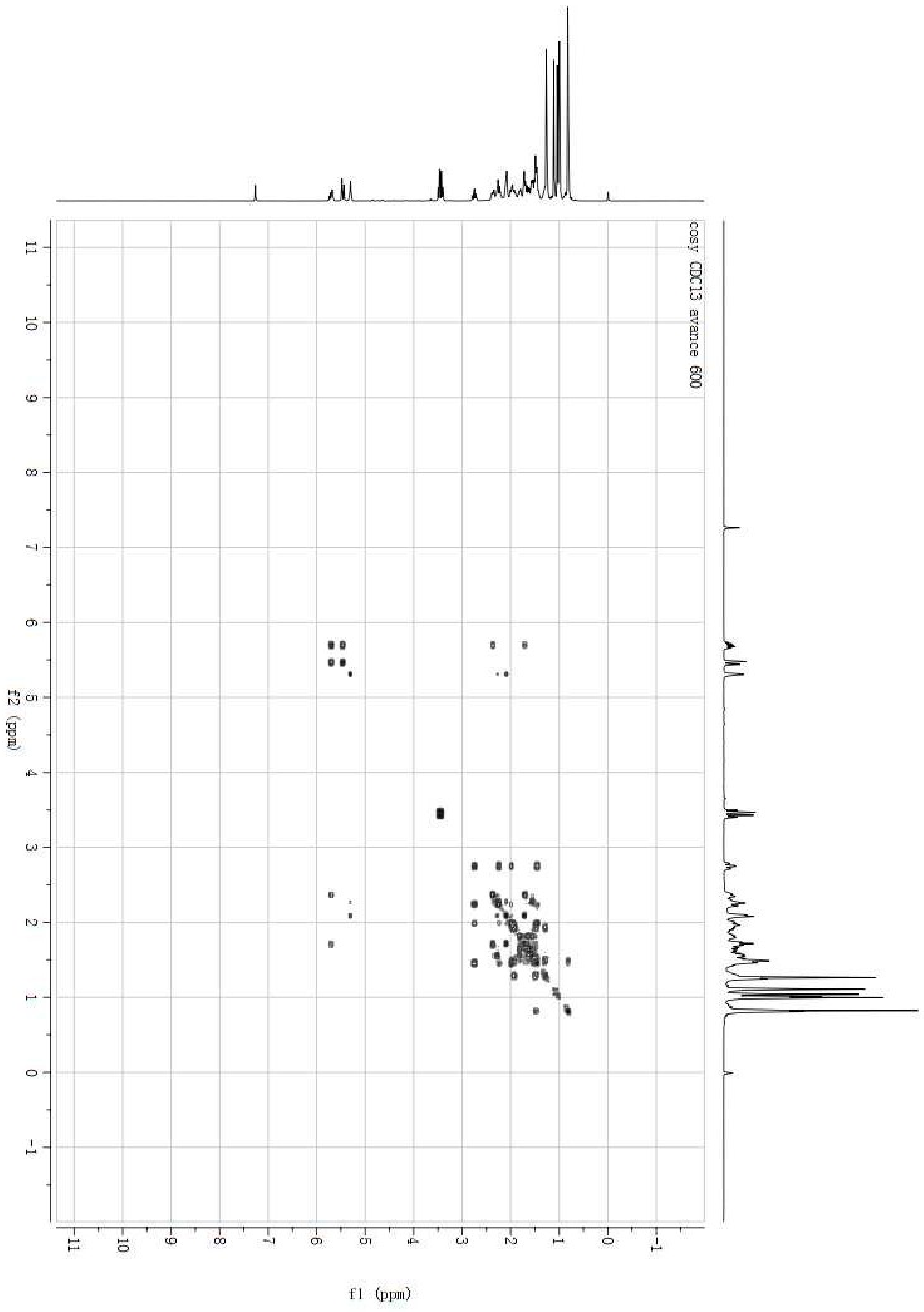

Fig. 49S HRMS spectra of 6

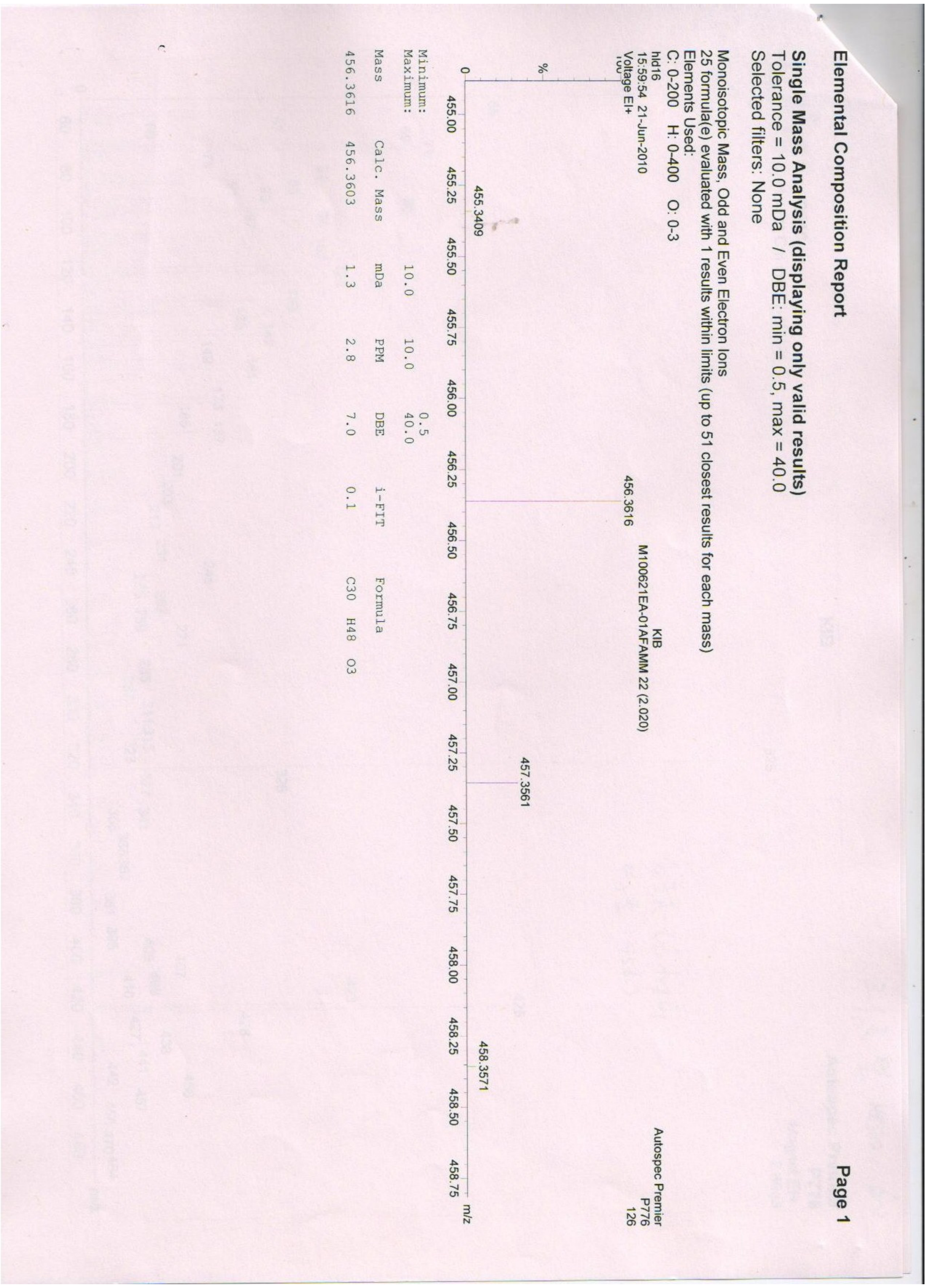

Elemental Composition Report Page 1

Single Mass Analysis (displaying only valid results)
Tolerance = 10.0 mDa / DBE: min = 0.5, max = 40.0
Selected filters: None

Monoisotopic Mass, Odd and Even Electron Ions
25 formula(e) evaluated with 1 results within limits (up to 51 closest results for each mass)
Elements Used:
C: 0-200 H: 0-400 O: 0-3

hld16 KIB
15:59:54 21-Jun-2010 M100621EA-01AFAMM 22 (2.020) Autospec Premier P776 126
Voltage EI+

Minimum:				0.5		
Maximum:		10.0	10.0	40.0		
Mass	Calc. Mass	mDa	PPM	DBE	i-FIT	Formula
456.3616	456.3603	1.3	2.8	7.0	0.1	C30 H48 O3

Fig. 50S IR spectra of 6

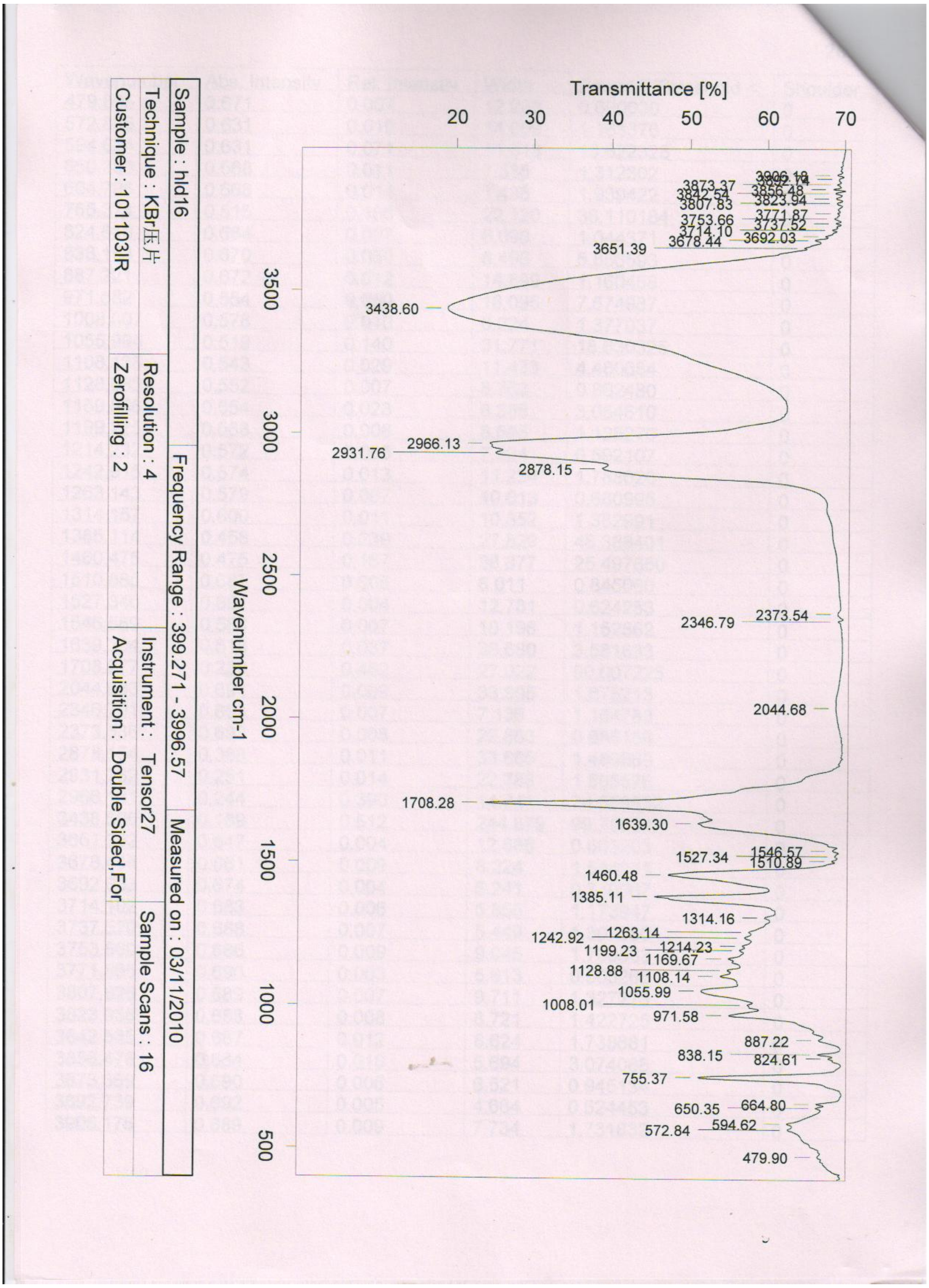

Fig. 51S Optical rotation spectra of 6

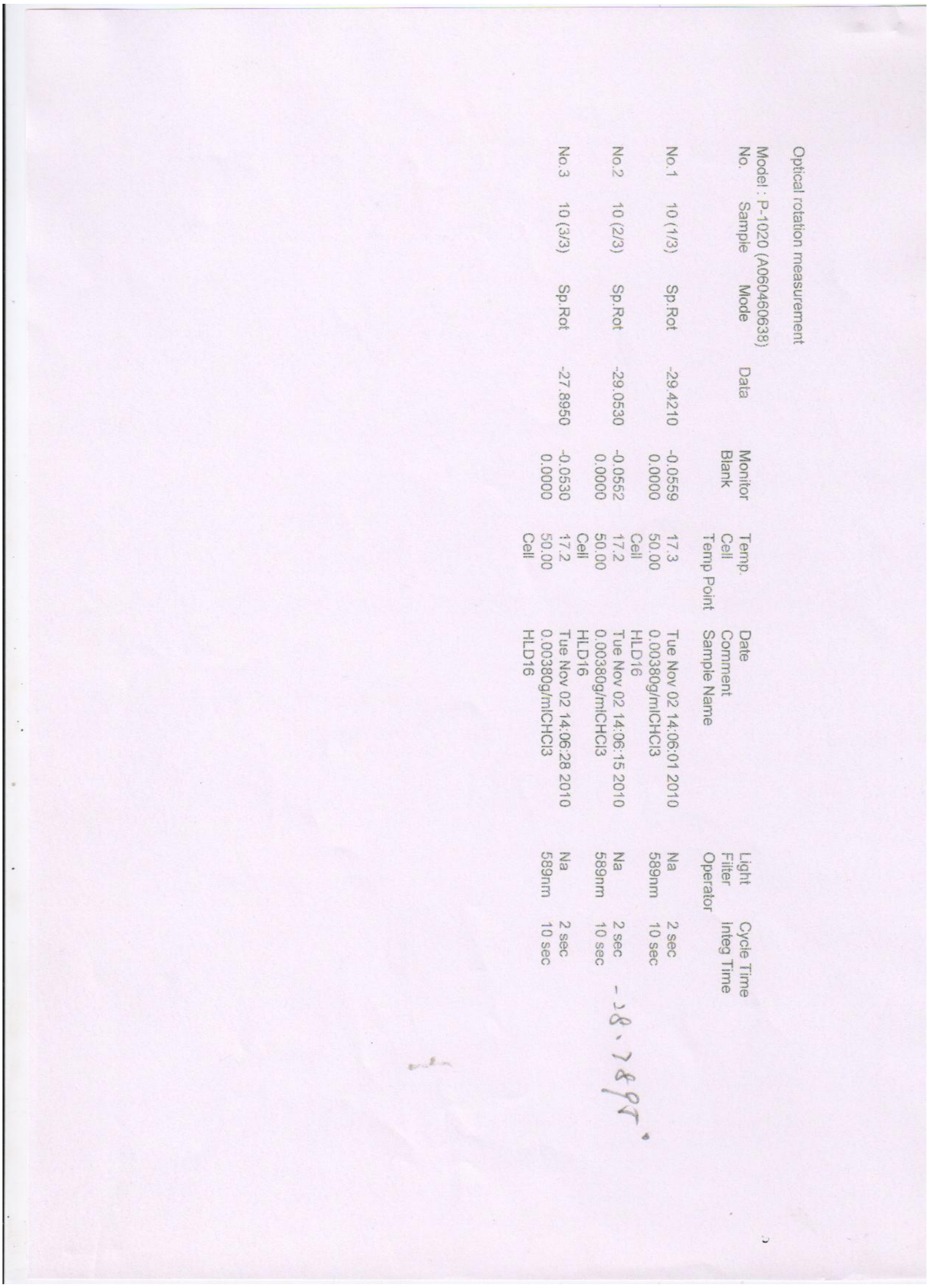

Optical rotation measurement

Model : P-1020 (A060460638)

No.	Sample	Mode	Data	Monitor<br>Blank	Temp.<br>Cell<br>Temp Point	Date<br>Comment<br>Sample Name	Light<br>Filter<br>Operator	Cycle Time<br>Integ Time
No.1	10 (1/3)	Sp.Rot	-29.4210	-0.0559<br>0.0000	17.3<br>50.00<br>Cell	Tue Nov 02 14:06:01 2010<br>0.00380g/mlCHCl3<br>HLD16	Na<br>589nm	2 sec<br>10 sec
No.2	10 (2/3)	Sp.Rot	-29.0530	-0.0552<br>0.0000	17.2<br>50.00<br>Cell	Tue Nov 02 14:06:15 2010<br>0.00380g/mlCHCl3<br>HLD16	Na<br>589nm	2 sec<br>10 sec
No.3	10 (3/3)	Sp.Rot	-27.8950	-0.0530<br>0.0000	17.2<br>50.00<br>Cell	Tue Nov 02 14:06:28 2010<br>0.00380g/mlCHCl3<br>HLD16	Na<br>589nm	2 sec<br>10 sec

Fig. 52S Selected COSY correlations and HMBC correlations of 1-6:

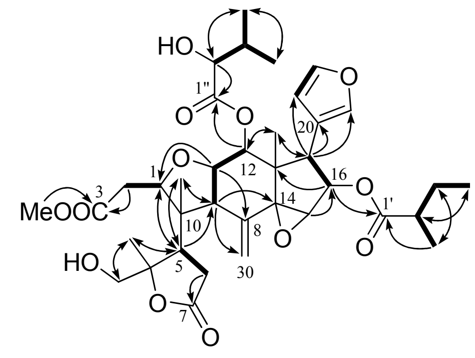

1

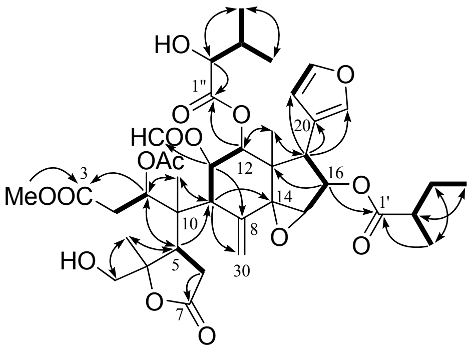

2

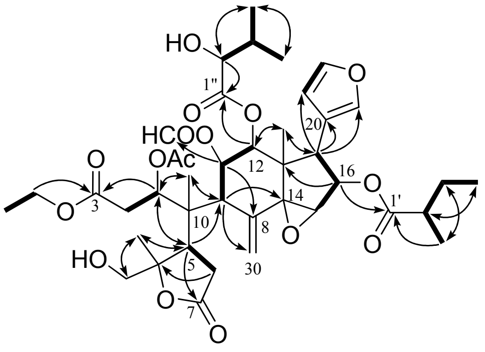

3

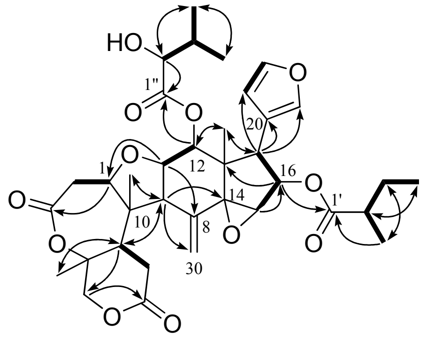

4

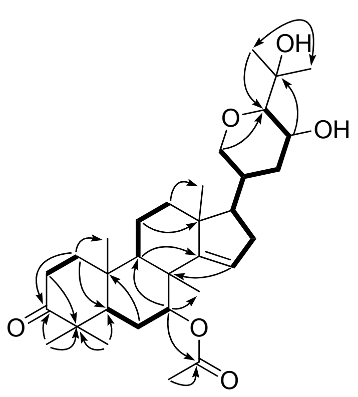

5

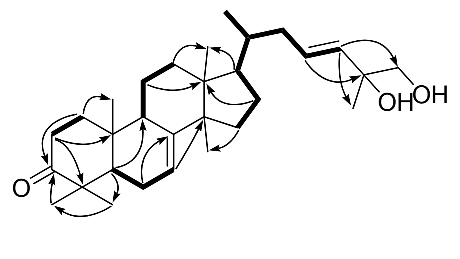

6

━━ ^{1}H-^{1}H COSY

── HMBC
